# Core‐Twisted, Cationic Perylene Diimides; Homochiral Dimerization and Chiroptical Anion Sensing

**DOI:** 10.1002/chem.202501270

**Published:** 2025-05-02

**Authors:** Denis Hartmann, Jamie S. Hillis, Lucy E. Walker, Timothy A. Barendt

**Affiliations:** ^1^ School of Chemistry University of Birmingham Edgbaston Birmingham B15 2TT UK

**Keywords:** chiral anion recognition, chiroptical sensing, dimer, perylene diimide, supramolecular chemistry

## Abstract

Core‐twisted perylene diimides (PDIs) are chiral organic dyes that may be exploited for self‐assembled chiroptical materials or for the enantioselective recognition and sensing of chiral substrates. Discrete self‐assembled dimers and host–guest complexes of core‐twisted perylene diimides are important for furthering our understanding of this supramolecular chemistry, yet they are rare because the twisted perylene core significantly weakens intermolecular π–π interactions with the PDI's π‐surface. To address this challenge, we have installed hydrogen bond donor groups in the PDI's bay positions, which direct the formation of a robust, co‐facial and homochiral intermolecular PDI dimer. The structure of this discrete dimer is distinct from previous aggregates of non‐planar PDIs that utilize the imide position for hydrogen bonding. We also uncover the potential of core‐twisted, dicationic PDIs for the enantioselective recognition and chiroptical sensing of chiral anions and investigate the basis of this response via chiral complementarity in the discrete host–guest complex.

## Graphical abstract

The installation of two 3‐methyl‐1,2,3‐triazolium heterocycles on the aromatic core of a perylene diimide (PDI) generates a twisted and cationic organic dye. As potent hydrogen bond donors, these groups enable the PDI to self‐assemble into discrete and robust homochiral dimers in solution. Furthermore, the enantioselective recognition of chiral anions is exploited for chiroptical sensing.

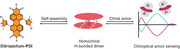

## 1 Introduction

**Figure 1:**
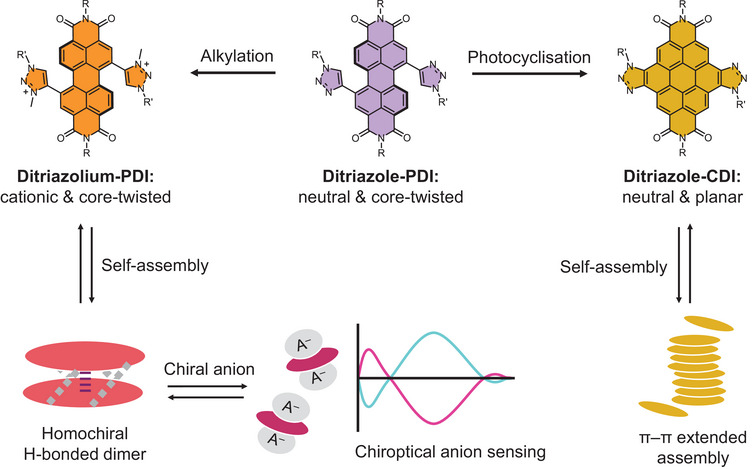
An overview of this work. Rylene diimide self‐assembly is enabled through either dye alkylation (left) or planarization (right). In the former case, the ditriazolium PDI generates a discrete intermolecular dimer which, upon chiral anion (A⁻) binding, undergoes disassembly and affords chiroptical anion sensing via the induction of a circular dichroism spectrum (bottom).

Rylene diimides are organic dye molecules with outstanding photophysical, electrochemical and, in some cases, chiroptical properties.^[^ ^1^, ^2^, ^3^ ^]^ This, in combination with the supramolecular chemistry of these dyes,^[^ ^4^ ^]^ is motivating the current development of functional self‐assembled materials, including for energy storage,^[^ ^5^ ^]^ organic (opto)electronics,^[^ ^6^ ^]^ and chemical sensing.^[^ ^7^ ^]^ The relative arrangement of the rylene diimide dictates interchromophore electronic coupling, which is critical to tuning material properties and optimizing performance towards the target application.^[^ ^8^ ^]^ In this context, discrete assemblies of perylene diimides (PDIs),^[^ ^9^ ^]^ such as dimers,^[^ ^10^, ^11^, ^12^, ^13^, ^14^ ^]^ have significant value since they provide model systems with which to assess structure–property relationships in extended supramolecular materials. However, for planar PDI or π‐extended coronene diimide (CDI) dyes, self‐assembly invariably leads to the formation of extended aggregates, since it is driven by π–π stacking between their equivalent π‐surfaces (**Figure** **1**, right).^[^ ^15^, ^16^ ^]^

The substitution of a PDI's bay positions provides a strategy for tuning its self‐assembly,^[^ ^17^ ^]^ with twisting of the perylene core also introducing intrinsic chirality^[^ ^18^ ^]^ (*M*/*P*) and the potential for chiroptical properties.^[^ ^1^ ^]^ However, as well as hindering co‐facial dye arrangements with H‐type electronic coupling,^[^ ^4^, ^19^ ^]^ PDI core‐twisting is notorious for reducing the strength of self‐assembly by several orders of magnitude.^[^ ^17^ ^]^ To increase self‐affinity, PDIs have been modified to utilize intermolecular hydrogen bonding.^[^ ^4^, ^20^, ^21^, ^22^ ^]^ This includes the preparation of elegant supramolecular fibres from core‐twisted PDIs that, through cooperative π–π stacking and hydrogen bonding, adopt slipped stacked assemblies with J‐type coupling.^[^ ^23^, ^24^, ^25^ ^]^ However, as seen in these systems and many other PDI derivatives,^[^ ^26^, ^27^, ^28^, ^29^ ^]^ hydrogen bonding is typically engineered via the imide positions and so generates extended assemblies. Therefore, without the use of covalent linkages,^[^ ^30^, ^31^ ^]^ discrete intermolecular dimers between core‐twisted PDIs are rare^[^ ^11^, ^12^ ^]^ and unprecedented with hydrogen bonding.

Discrete (supra)molecular organic dyes are also befitting as optical probes in bioimaging^[^ ^32^ ^]^ and chemical sensing.^[^ ^33^, ^34^, ^35^ ^]^ For the latter, PDIs have been widely implemented as a photophysical/electrochemical reporter group for the recognition of metal cations and biomolecules.^[^ ^7^ ^]^ Alternatively, the binding of aromatic substrates at the PDI's π‐surface(s) has been exploited for optical sensing,^[^ ^36^, ^37^, ^38^ ^]^ including the use of core‐twisted PDIs for the enantioselective recognition of cationic^[^ ^39^ ^]^ and neutral^[^ ^40^, ^41^, ^42^ ^]^ aromatic guests. However, despite the importance of anion recognition to synthetic chemistry, biology and the environment,^[^ ^43^ ^]^ PDIs capable of binding and sensing anions^[^ ^44^, ^45^, ^46^ ^]^ are much less prevalent and often rely on undefined extended aggregates.^[^ ^47^, ^48^, ^49^, ^50^ ^]^ Moreover, there are no reports of PDIs capable of the chiroptical sensing of chiral anions.

In this work, we prepared a novel series of rylene diimides to investigate the impact of the dye's structure, including planarity, charge, and core‐substitution pattern, on its (chiral) self‐assembly and (chiral) anion recognition properties (**Figure** **1**). Regioisomerically pure 1,6/1,7‐disubstituted PDIs were targeted with the aim of identifying structure(s) that render the dye's π‐surfaces inequivalent, to direct self‐assembly into discrete dimers. Here, 1,2,3‐triazole bay substituents^[^ ^51^ ^]^ give access to ditriazole PDIs, ditriazolium PDIs and a ditriazole CDI, to examine chromophore planarity and charge (**Figure** **1**), as well as the possibility of exploiting hydrogen bonding using these heterocycles.^[^ ^52^, ^53^, ^54^, ^55^, ^56^ ^]^ The novel 1,7‐ditriazolium PDI is notable for forming a discrete intermolecular dimer (**Figure** **1**, left), with the unique positioning of the triazolium‐based hydrogen bond donors affording a robust, co‐facial and homochiral dimer between two core‐twisted PDIs. The potential anion‐binding properties of these cationic PDIs are also explored, with triazolium–chromophore π‐conjugation affording discrete PDI‐based anion sensors. Moreover, for the first time, we demonstrate that cationic, core‐twisted PDIs can be used for the enantioselective recognition and chiroptical sensing of chiral aromatic anions. This discovery also sheds light on the role of chiral complementarity in directing the formation of homo/heterochiral complexes between non‐planar aromatics.

## 2 Results and Discussion

### 2.1 Synthesis and Characterization

The synthesis of the target PDI and CDI dyes began with the preparation of a dibrominated PDI with solubilizing branched (C5) alkyl chains at the imide positions, according to published procedures.^[^ ^57^ ^]^ The reaction yields both the 1,7‐ and 1,6‐substituted PDI regioisomers, **1a** and **1b** respectively. Since regular silica gel chromatography is unable to separate these regioisomers, we employed high‐performance liquid chromatography using pyrene‐modified silica as our stationary phase, allowing for the chromatographic separation of both **1a** and **1b** regioisomers on a preparative scale (Supporting Information).^[^ ^58^ ^]^

With regioisomerically pure dibrominated PDIs in hand, we proceeded by installing alkyne groups using Sonogashira cross‐coupling conditions. This reaction yielded the desired, trimethylsilyl‐protected dialkyne PDIs in quantitative yield for both regioisomers (**2a,b**). Deprotection with a base, followed by copper‐mediated alkyne‐azide cycloaddition enabled the installation of *n*‐octyl‐alkylated 1,2,3‐triazoles onto the PDI core to give the 1,7‐ and 1,6‐regioisomers **3a** and **3b**, which were characterized by ^1^H and ^13^C NMR spectroscopy and high‐resolution mass spectrometry. Single crystals of 1,7‐ditriazole PDI **3a** were grown that were suitable for x‐ray diffraction (XRD).^[^ ^59^ ^]^ Notably, the crystal structure of **3a** shows a relatively planar perylene core, accommodated by the positioning of the triazole heterocycles above and below the PDI plane. This conformation is contrary to the typical twisted perylene core^[^ ^18^ ^]^ of disubstituted PDIs (e.g., see crystal structure of **4a**) and likely arises from solid‐state effects.^[^ ^60^ ^]^ Indeed, upon density functional theory (DFT) optimization of the crystal structure of **3a** (Supporting Information S5‐1 and S5‐2, B97‐3c,^[^ ^61^ ^]^ def2‐TZVP^[^ ^62^ ^]^), the 1,7‐ditriazole PDI is found to exhibit a twisted perylene core (dihedral angle = 21°). This also leads to the triazole heterocycles being positioned on the same face of the PDI and rotated with respect to the plane of each naphthalene sub‐unit (dihedral angle = 43°).

**Figure 2:**
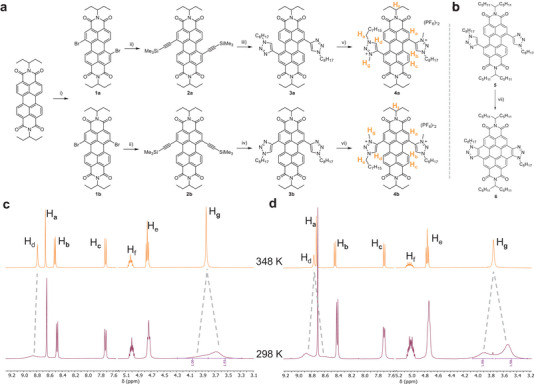
Synthesis of 1,7‐ and 1,6‐ditriazolium PDIs **4a** and **4b** respectively. (a) (i) Br_2_, CHCl_3_, 60 °C, then HPLC. (ii) TMS‐acetylene, Pd(PPh_3_)_2_Cl_2_, CuI, PhMe, NEt_3_. (iii) K_2_CO_3_, MeOH:DCM, then Cu(MeCN)_4_PF_6_, *n*‐octyl azide, DCM. (iv) TBAF, THF, then Cu(MeCN)_4_PF_6_, *n*‐octyl azide, DCM. (v) MeI, 80 °C, 3 days, then NH_4_PF_6_. (vi) MeI, 25 °C, 3 weeks, then NH_4_PF_6_. (b) Synthesis of 1,7‐ditriazole CDI **6**. (vii) 525 nm LED, CHCl_3_. (c) Partial ^1^H NMR spectrum of 1,7‐ditriazolium PDI **4a** at 298 K (purple spectrum) and 348 K (orange spectrum). (d) Partial ^1^H NMR spectrum of 1,6‐ditriazolium PDI **4b** at 298 K (purple spectrum) and 348 K (orange spectrum). All ^1^H NMR spectra were recorded in MeCN‐*d*_3_ (400 MHz).

For comparison to core‐twisted PDIs, we also prepared a planar CDI derivative. To do this, we performed a green‐light catalyzed Mallory photocyclization^[^ ^51^ ^]^ on **3a** to access the target 1,7‐ditriazole CDI (Supporting Information Section 1). However, the desired product was poorly soluble in common organic solvents, which limited its purification and characterization. Therefore, we synthesized a more soluble analogue, 1,7‐ditriazole CDI **6**, which possesses longer branched (C11) alkyl chains at the imide positions (Figure 2b).

To prepare the analogous 3‐methyl‐1,2,3‐triazolium‐based PDIs, we first dissolved 1,7‐ditriazole PDI **3a** in neat methyl iodide and heated it in a pressure tube at 80 °C for three days to give the 1,7‐ditriazolium PDI **4a**, which was fully characterized by ^1^H and ^13^C NMR spectroscopy, high‐resolution mass spectrometry and single crystal XRD.^[^ ^59^ ^]^ These methylation conditions did not yield the desired 1,6‐ditriazolium PDI **4b** and led to degradation. Instead, the 1,6‐regioisomer was alkylated by stirring **3b** in neat methyl iodide at room temperature for three weeks, which, while slow, showed only negligible degradation, and allowed for the isolation of the target **4b** in respectable yield. The resulting iodide salts of ditriazolium PDIs **4a** and **4b** were then exchanged to their hexafluorophosphate salts, the latter being a weakly‐coordinating anion^[^ ^63^ ^]^ to facilitate self‐assembly and anion binding studies (*vide infra*).

The ^1^H NMR spectra of both 1,7‐ and 1,6‐ditriazolium PDIs (**4a** and **4b**) showed marked differences to their parent ditriazole PDI compounds, with the aromatic PDI protons shifting by up to 0.5 ppm and the triazole protons by +1 ppm in the triazolium compounds. Moreover, at room temperature in MeCN‐*d_3_*, both the triazolium H_d_ (C─H) and H_g_ (3‐methyl) signals are broad and are split into two signals in an approximate ratio of 2:1, as most clearly seen in **4b**. Variable temperature ^1^H NMR spectroscopy in MeCN‐*d_3_* revealed that these triazolium proton signals sharpen and coalesce upon heating, giving the expected two singlets for H_d_ and H_g_ (Figure 2c,d, Supporting Information Figures S6‐1 and S6‐2). Coalescence occurs at a similar temperature in both **4a** and **4b** and for both H_d_ and H_g_.

**Figure 3:**
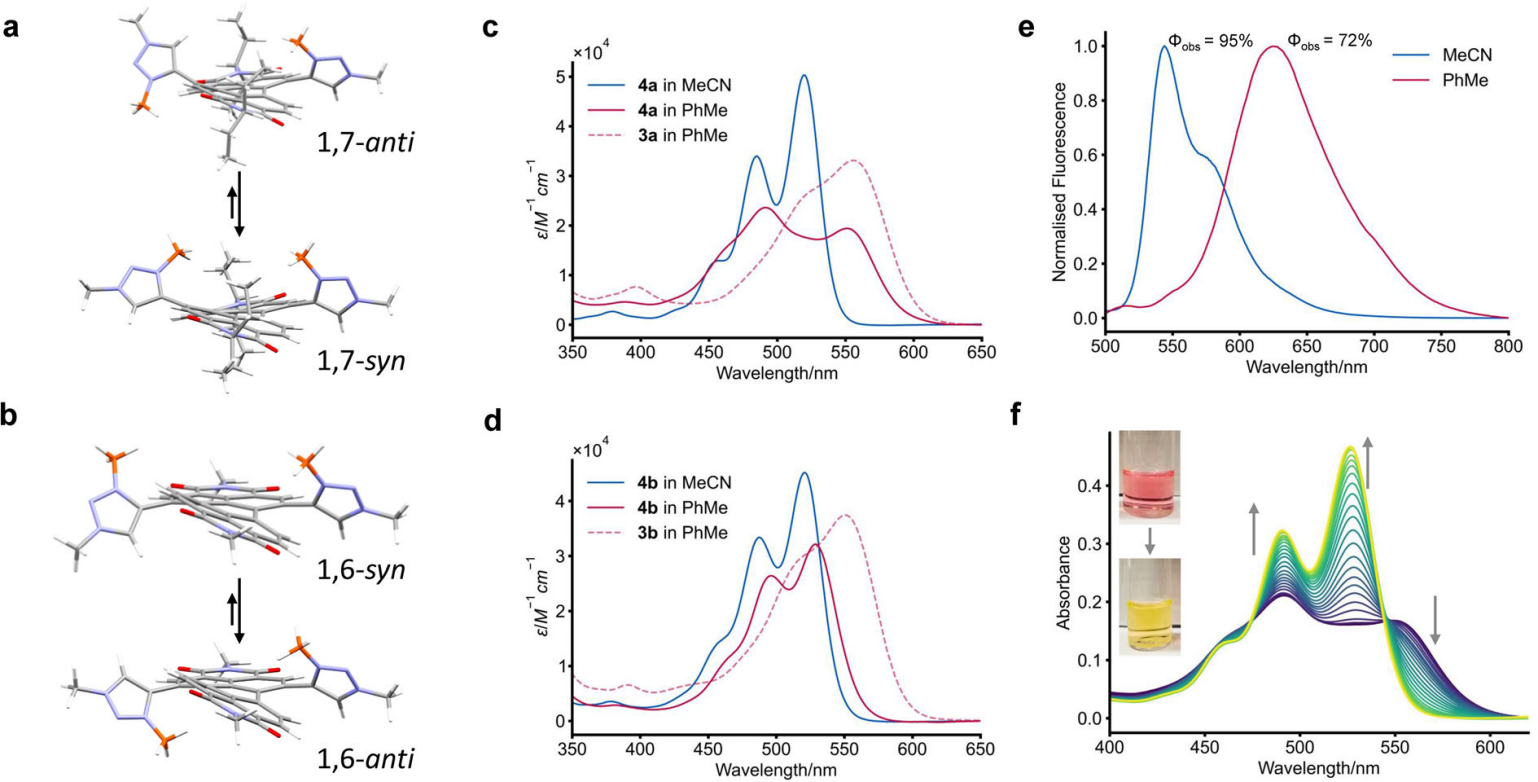
(a) The *syn*‐ and *anti*‐rotamers of 1,7‐ditriazolium PDI **4a** as seen in the crystal structure (C8 chains removed for clarity). (b) The *syn*‐ and *anti*‐rotamers of 1,6‐ditriazolium PDI **4b** as predicted by CREST (two lowest‐energy conformers). For both regioisomers, the triazolium's 3‐methyl groups have been colored orange to highlight the syn/anti rotamers, the energetic ordering of which is predicted by DFT. UV‐vis absorption spectra of **4a** (c) and **4b** (d). (e) Normalized fluorescence emission spectra of **4a**. (f) A MeCN (yellow line)‐into‐PhMe (purple line) solvent‐into‐solvent UV‐vis spectroscopic titration for **4a** showing dimer→monomer disassembly (direction of grey arrows) upon addition of the polar solvent.

The conformational landscape of the 1,7‐ditriazolium PDI **4a** is rather complex, with three possible enantiomeric pairs of conformations (Supporting Information Figure S5‐3). These are related by rotation along the triazolium–PDI bond, generating *syn*/*anti* rotamers, or helical twisting of the perylene core, generating *M*/*P* stereoisomers, both of which are evident from the XRD crystal structure of **4a** (Figure 3a). If the latter is slow on the ^1^H NMR timescale, we would expect three sets of signals for each of the triazolium protons H_d_ and H_g_. However, *syn*/*anti* rotamers will generate two sets of signals, which is consistent with the experimental data. Therefore, from the variable temperature ^1^H NMR spectra, we can estimate the Gibbs free energy barrier for the interconversion of *syn*/*anti* rotamers; Δ*G* ^‡^ = 61 kJ mol⁻^1^ in both **4a** and **4b**. The Δ*G* ^‡^ is expected to be similar in both regioisomers due to the similar environments for the triazolium groups on either side of the perylene core. Notably, the same conformational analysis for **4b** (Supporting Information Figure S5‐4) gives two enantiomeric pairs of conformations and again only *syn*/*anti* rotamers are consistent with the splitting of H_d_ and H_g_ into two sets of signals.

Since we could not obtain single crystals of the 1,6‐ditriazolium PDI suitable for x‐ray diffraction, we performed a conformer search on **4b** using the CREST code^[^ ^64^ ^]^ and the GFN2‐xTB tight‐binding method^[^ ^65^ ^]^ (Supporting Information Figures S5‐5 and S5‐6). In both cases *syn*‐ and *anti*‐rotamers (*M*‐chirality) were predicted by CREST. We then optimized the geometry of the lowest energy *syn*‐ and *anti*‐rotamers using DFT (B97‐3c basis set^[^ ^61^, ^66^ ^]^ and the def2‐TZVP functional^[^ ^62^ ^]^) and calculated their single point energies (Supporting Information Figures S5‐7 and S5‐8). We performed the same procedure for the 1,7‐ditriazolium PDI regioisomer **4a**, which validated our computational methods due to excellent agreement with the structures identified by XRD (Supporting Information Figures S5‐7,S7‐7 and S7‐8).

The *syn*‐rotamer is predicted to be lower in energy for the 1,7 regioisomer **4a**, while the *anti*‐rotamer is favoured by **4b**. This is consistent with the similar ^1^H NMR spectra of **4a** and **4b**; the environments of the triazolium's 3‐methyl groups are similar in both the lowest energy rotamers which leads to the comparable splitting of H_g_ (at room temperature). The regioisomers have a similar energy difference between their *syn*/*anti* rotamers, which is also consistent with their similar population ratios from ^1^H NMR spectroscopy. We finally used DFT to estimate the interconversion barrier between *M*/*P* stereoisomers in **4a**, Δ*G* ^‡^ = 36 kJ mol⁻^1^ (Supporting Information Figures S5‐9 and S5‐10). As expected, this barrier is lower than Δ*G* ^‡^ for *syn*/*anti* interconversion (61 kJ mol⁻^1^) and indicates that *M*/*P* chirality in ditriazolium PDIs is dynamic (at room temperature), providing an opportunity for supramolecular chiral induction (*vide infra*).

Aside from the triazolium H_d_ (C─H) and H_g_ (3‐methyl) signals, the ^1^H NMR spectrum of **4a** is sharp in MeCN‐*d_3_* but becomes uniformly broader in lower polarity solvents such as CDCl_3_ and PhMe‐*d_8_*, indicative of intermolecular aggregation. Therefore, due to the characteristic absorption and emission spectra of PDI aggregates,^[^ ^8^ ^]^ we turned to photophysical techniques to explore the supramolecular self‐assembly of our dyes.

### 2.2 Photophysics

We measured the electronic absorption spectra of the 1,6‐ and 1,7‐ditriazole (**3a**,**b**) and ditriazolium PDIs (**4a**,**b**) in a range of different solvents (Figure 3c,d, Supporting Information Figures S2‐1 and S2‐2). The main PDI absorption band (S_0_→S_1_) of the dicationic PDIs **4a,b** is blue‐shifted compared to the analogous neutral bis‐triazole PDIs **3a,b** (Δ*λ*_max_ up to 27 nm), which has been observed with other chromophores when π‐conjugated to 1,2,3‐triazolium heterocycles.^[^ ^67^, ^68^ ^]^ The blue‐shift shift may in part be explained by a reduction in π‐conjugation due to an increased rotation of the PDI–triazolium bond and/or increased twisting of the perylene core^[^ ^12^ ^]^ upon methylation of the triazole. The former appears most likely since our calculated (and XRD) structures show the PDI–triazolium dihedral angle increases on going from **3a** (43°) to **4a** (58°), while the dihedral angles of the twisted perylene core are similar (21° vs. 19°, Supporting Information Figure S5‐2). The vibrational progression of the main PDI absorption band is also more defined in **4a,b** relative to **3a,b**, which is consistent with the greater rigidity of the ditriazolium PDIs (i.e., their propensity to adopt *syn*/*anti* rotamers).

For both **4a** and **4b**, the ratio of the 0‐0 and 0‐1 vibronic peak intensities (A_0‐0/0‐1_) is consistent with monomeric chromophores in polar protic (e.g., MeOH) and polar aprotic (MeCN) solvents. The neutral ditriazole PDI dyes **3a**,**b** are also monomeric in organic solvents. Interestingly however, in solvents of lower polarity (e.g., PhMe, CHCl_3_), the 1,7‐bistriazolium PDI **4a** undergoes a significant decrease in A_0‐0/0‐1_ (from 1.46 to 0.82), which is evidence for a large increase in H‐type excitonic coupling between dyes and the formation of a co‐facial π‐stack.^[^ ^4^ ^]^ A solvent into solvent titration revealed that the intermolecular assembly in PhMe is readily disrupted by small amounts of MeCN (Figure 3f, Supporting Information Figure S2‐3). The significant change in the absorption spectrum of **4a** ongoing between PhMe and MeCN results in a colour change that is visible to the naked eye (Figure 3f). By contrast, the 1,6‐ditriazolium PDI regioisomer **4b** exhibits no significant spectral change in PhMe or CHCl_3_ and hence no appreciable self‐assembly (Figure 3d). These trends are also reflected in the fluorescence emission spectra of **4a** and **4b**. While monomeric emission is observed for the 1,7‐ and 1,6‐ditriazolium PDIs in MeCN (Supporting Information Figure S2‐4), only the former changes in PhMe (Figure 3e), instead giving a broader and featureless emission profile with a larger Stokes shift (*λ* = 132 vs. 24 nm) and a lower fluorescence quantum yield (*Φ*_obs_ = 95% vs. 72%). This is characteristic of H‐type coupling^[^ ^4^ ^]^ and the formation of an excimer^[^ ^69^ ^]^ between **4a** PDIs in PhMe.

### 2.3 Self‐Assembly

**Figure 4:**
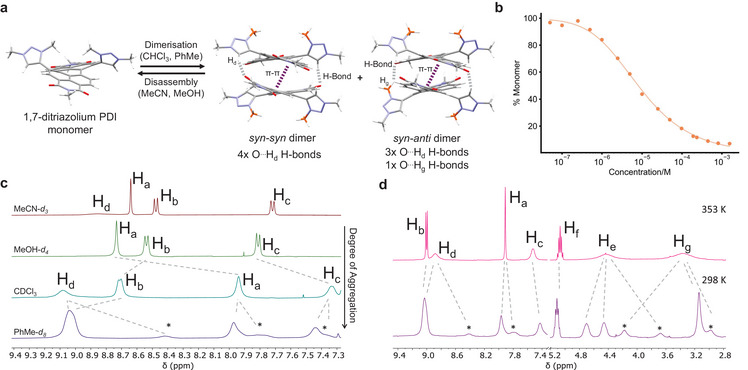
(a) The dimerization of 1,7‐ditriazolium PDI **4a** into *syn–syn* (major) and *syn–anti* (minor) dimers. These are shown here as an XRD and DFT structure respectively, and both with relevant non‐covalent interactions highlighted. C8‐alkyl chains are shown simplified as Me groups. (b) The % monomer of PDI **4a** plotted as a function of its concentration in CHCl_3_, with the curve determined by fitting the concentration‐dependent UV‐vis spectra (A_0‐0_/A_0‐1_) to the dimer model (*K*_dim_). (c) ^1^H NMR spectra of **4a** in various solvents (298 K, 400 MHz), with an increasing degree of dimerization from top to bottom (* = minor *syn–anti* dimer). (d) Variable temperature ^1^H NMR spectra of **4a** in PhMe‐*d*_*8*_ (400 MHz), with the assignment of coalescing peaks from EXSY NMR spectroscopy.

We quantified the strength of the supramolecular assembly formed by **4a**, in fact, a PDI dimer (*vide infra*, Figure 4a), by measuring its absorption spectrum at different concentrations (Supporting Information Figure S3‐1). This study was performed in CHCl_3_ since in PhMe the dimer was too strong to be disrupted upon dilution. Non‐linear fitting of the binding isotherm to the dimer model gave a dimerisation constant of *K*_dim_ = 1 × 10^5^ M⁻^1^ for **4a** (Figure 4b),^[^ ^70^ ^]^ which is significant considering that the assembly is composed of two core‐twisted and dicationic PDIs. The dimer model was used because in the crystal structure of **4a**, the 1,7‐ditriazolium PDIs form a co‐facial and homochiral dimer, held together by aromatic stacking and hydrogen bonding interactions (Figure 4a). Indeed, previous work has shown that core‐twisted PDIs favor the formation of dimers over extended aggregates in solution,^[^ ^17^ ^]^ with the substitution pattern of 1,7‐disubstituted PDIs favoring dimerization at the least sterically hindered face,^[^ ^31^ ^]^ as seen in the crystal structure of the *syn*–*syn* dimer of **4a** (Figure 4a).

We used computational methods to support our analysis of the dimer's structure in solution. A CREST conformer search identified the lowest energy dimer is one that is formed between two *syn*‐rotamers of **4a**. This structure was optimized by DFT with implicit toluene solvation (B97‐3c,^[^ ^61^, ^66^ ^]^ def2‐TZVP^[^ ^62^ ^]^) and was found to be in excellent agreement with the *syn*–*syn* dimer seen in the crystal structure of **4a** (Figure 4a and Supporting Information Figures S7‐9 and 7‐10). Alternative dimer structures containing one or two *anti*‐rotamers were also calculated (Supporting Information Figure S5‐11) since the *anti*‐rotamer of **4a** had been identified by ^1^H NMR spectroscopy in solution (Figure 2c) and in the XRD crystal structure (Figure 3a, Supporting Information Figure S7‐8). The energetic ordering of the three dimer types is predicted to be *syn*–*syn* < *syn*–*anti* << *anti*–*anti*. In the two lowest energy dimers, namely *syn*–*syn* (from XRD and DFT) and *syn*–*anti* (DFT), the PDI chromophores have a co‐facial arrangement and are rotationally displaced (α = 41°), which is consistent with the UV‐vis spectrum of **4a** in PhMe, specifically H‐type excitonic coupling (A_0‐0/0‐1_ = 0.82) with a red‐shifted A_0‐0_ band (Figure 3c).^[^ ^15^ ^]^ Alongside π–π stacking between the homochiral π‐surfaces of the PDIs (d_π–π_ = 3.4 Å), the *syn*–*syn* dimer is knitted together by four intermolecular hydrogen bonds between PDI carbonyl groups and all four polarized triazolium H_d_ protons, d(C═O^…^H_d_─C) = 2.2 Å. In the *syn*–*anti* dimer, the rotation of a triazolium heterocycle means that one of these O^…^H_d_ hydrogen bonds are replaced by a weaker O^…^H_g_ hydrogen bond involving the 3‐methyl group.^[^ ^71^ ^]^ Therefore, alongside poorer shape complementarity, this accounts for the *syn*–*anti* dimer being higher in energy than the *syn*–*syn* dimer. Intermolecular hydrogen bonding also explains why the *anti*‐rotamer of **4a**, and indeed both rotamers of the 1,6‐ditriazolium PDI **4b**, do not self‐assemble into dimers (or extended aggregates) in solution.^[^ ^72^ ^]^ Here, the position of the 3‐methyl triazolium groups means that each PDI face is sterically hindered and can only donate one C─H_d_‐based hydrogen bond, whereas the unhindered face of the *syn*‐rotamer of **4a** can donate two of these stronger hydrogen bonds.

Our structural analysis of the 1,7‐ditriazolium PDI dimer in solution was also supported by ^1^H NMR spectroscopy. Compared to the monomer of **4a** in MeCN‐*d_3_*, ^1^H NMR spectra in CDCl_3_ and PhMe‐*d_8_* revealed a significant broadening and shifting of the triazolium (H_d_) and PDI (H_a‐c_) protons, consistent with intermolecular hydrogen bonding and π–π stacking interactions (Figure 4c). Interestingly, in PhMe‐*d_8_*, the ^1^H NMR signals are all split into two sets of peaks in a 3:1 ratio. Considering the concentration of the ^1^H NMR spectrum (10⁻^3^ M) and the fact that dimerization is stronger in PhMe than CHCl_3_, the monomer population of **4a** is expected to be negligible in PhMe (i.e., << 5%, Figure 3b). Therefore, from our XRD and computational analysis, we assign the two sets of ^1^H NMR signals in PhMe‐*d_8_* to be the major *syn*–*syn* and minor *syn*–*anti* dimer conformations (Figure 4a, Supporting Information Figure S5‐11). This implies that dimerization increases the population of the *syn*‐rotamer of **4a** due to complementary non‐covalent interactions. The lower symmetry of the *syn*–*anti* dimer is consistent with its ^1^H NMR spectrum because the *anti*‐rotamer's H_g_ protons are split into two signals, one of which is shifted to higher ppm (*δ* = 4.2 ppm) due to the O^…^H_g_ intermolecular hydrogen bond involving the 3‐methyl group (Figure 4d). A minor H_d_ signal is also shifted to lower ppm (*δ* = 8.4 ppm), reflecting the loss of an intermolecular hydrogen bond in the *syn*–*anti* dimer.

EXSY NMR spectroscopy revealed that the distinct *syn*–*syn* and *syn*–*anti* dimers of **4a** are in exchange and their interconversion is slow on the NMR timescale at room temperature (Supporting Information Figure S6‐4). The major and minor sets of ^1^H NMR signals coalesce and sharpen upon heating (Figure 3d, Supporting Information Figures S6‐5 and S6‐6), giving Δ*G* ^‡^ = 66 kJ mol⁻^1^ for *syn*/*anti* rotamer interconversion in the dimer. This value is larger than that measured for the monomer, reflecting the stronger hydrogen bond network in the *syn*–*syn* versus *syn*–*anti* dimer. The coalescence of signals is not due to monomer formation since variable temperature UV‐vis spectroscopy showed that significant disassembly does not occur upon heating (Supporting Information Figures S3‐2 and S3‐3). The **4a** variable temperature ^1^H NMR spectra are also notable for the coalescence of two major H_e_ signals (i.e., from a *syn*–*syn* dimer), at a different temperature to those arising from *syn*/*anti* rotamers. These H_e_ signals are from the first methylene group of the triazolium's *n*‐octyl chain and they undergo diastereotopic splitting upon dimerization of **4a** due to the PDI dimer's (homo)chirality. Therefore, the coalescence of H_e_ enables us to estimate Δ*G* ^‡^ for the interconversion between the dimer's *MM/PP* stereoisomers, Δ*G* ^‡^ = 69 kJ mol⁻^1^. Intuitively, Δ*G* ^‡^ for the dimer of **4a** is effectively double that of the single 1,7‐ditriazolium PDI monomer (Δ*G* ^‡^ = 36 kJ mol⁻^1^).

Further evidence for the non‐covalent interactions that govern the **4a** monomer‐dimer equilibrium came from quantifying the degree of self‐assembly in a range of solvents (by UV‐vis spectroscopy) and examining its correlation with different solvent parameters (Supporting Information Figures S3‐4 and S3‐7). Alongside a correlation with solvent polarity parameters (e.g., Catalán SdP),^[^ ^73^ ^]^ we also found a correlation with the Kamlet–Taft scale of hydrogen‐bond accepting ability^[^ ^73^ ^]^ (Supporting Information Figure S3‐5), which supports the existence of intermolecular hydrogen bonding, as well as π–π stacking, in the 1,7‐ditriazolium PDI dimer. Polar solvents such as MeCN and MeOH typically result in the π–π (solvophobic‐driven) aggregation of PDIs,^[^ ^74^ ^]^ yet, for **4a**, their hydrogen bond‐accepting/‐donating properties yield monomers and restrict dimerization. Meanwhile, chlorinated solvents are amongst the best solvents for PDIs,^[^ ^75^ ^]^ yet **4a** readily dimerizes in CHCl_3_ since this solvent is less effective at disrupting intermolecular hydrogen bonding. The neutral 1,7‐ditriazole PDI **3a** does not dimerize in CHCl_3_ (or PhMe) because the neutral 1,2,3‐triazole is a weaker (C─H_d_) hydrogen bond donor than the cationic 3‐methyl‐1,2,3‐triazolium heterocycle (Supporting Information Figures S3‐8 and S3‐9).^[^ ^52^ ^]^

Finally, we used UV‐vis spectroscopy to investigate the self‐assembly of the 1,7‐ditriazole CDI **6**, envisaging that this planar dye might be more amenable to intermolecular π–π stacking despite photocyclization of the 1,2,3‐triazole now preventing H_d_‐based hydrogen bonding. Indeed, upon increasing the concentration of CDI **6** in cyclohexane, we observe a broadening of the absorption spectrum and a change in the relative vibronic peak intensities, indicative of self‐assembly (Supporting Information Figure S3‐10). The binding isotherm was fitted to the isodesmic model (Supporting Information Figure S3‐11) giving K_a_ = 4 × 10^5^ M⁻^1^,^[^ ^70^ ^]^ which is notable considering it is of the same order of magnitude as *K*_dim_ for the dicationic and core‐twisted PDI **4a**. The isodesmic model was chosen because both π‐surfaces of the CDI are equivalent, enabling the uniform growth of an extended supramolecular assembly.^[^ ^76^ ^]^

### 2.4 (Chir)optical Anion Sensing

3‐Methyl‐1,2,3‐triazolium‐based compounds are well‐known for anion recognition^[^ ^52^, ^54^, ^55^ ^]^ and so, upon π‐conjugation with a (chiral) PDI chromophore, we envisaged that our 1,7‐ and 1,6‐ditriazolium PDIs **4a** and **4b** may act as (chir)optical anion sensors. Initial binding studies were performed in MeCN, the solvent in which both PDI regioisomers are monomeric. UV‐vis spectroscopic titrations revealed chloride binding to PDIs **4a** and **4b** (as their hexafluorophosphate salts) by the evolution of the S_0_→S_1_ PDI absorption band giving rise to isosbestic points (Supporting Information Figures S4‐1 and S4‐2).^[^ ^38^ ^]^ Association constants were obtained by non‐linear curve fitting (**Table** **1**), with the best fit achieved with a 1:2 host‐guest stoichiometric model,^[^ ^77^ ^]^ which is sensible considering the PDI host is dicationic.^[^ ^78^, ^79^, ^80^ ^]^ Notably, the binding of the first chloride anion is stronger in the 1,6‐regioisomer **4b** relative to the 1,7 analogue (**4a**), which we tentatively ascribe to the different geometry of the C─H_d_ hydrogen bond‐donating groups, which are conjugated to the same naphthalene sub‐unit of the PDI in **4b**. For **4a** and **4b**, chloride binding is anti‐cooperative (i.e., *K*_1_ >> *K*_2_), which is expected if both triazolium groups partake in binding the first anion and so a conformational change must occur to accommodate the second anion.^[^ ^81^ ^]^ Chloride binding was also detected by fluorescence spectroscopy, with the halide causing a significant quenching of the PDI emission (Supporting Information Figure S4‐3).

**Table 1:** Anion association constants (*K*) determined by UV‐vis spectroscopic titrations and fitted to a 1:2 host–guest binding model in the region where the largest spectral change occurs. All fitting errors are <5%. All anions were titrated as their tetrabutylammonium salts and PDI host compounds used as their hexafluorophosphate salts.

Index	Compound	Anion	Solvent	*K*_1_	*K*_2_
1	**4a**	Chloride	MeCN	1100	60
2	**4b**	Chloride	MeCN	3000	90
3	**4a**	Chloride	PhMe	77,000	97
4	**4a**	(−)‐BINOL‐phosphate	PhMe	46,000	2800
5	**4a**	Δ‐TRISPHAT	PhMe	48,000	260
6	**4a**	(+)‐10‐camphorsulfonate	PhMe	290,000	8000

**Figure 5:**
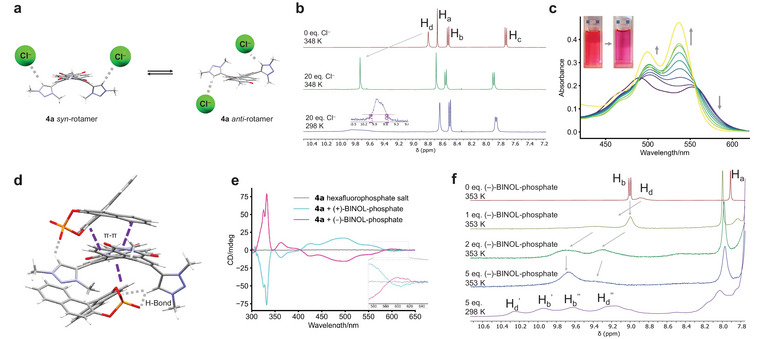
Anion binding of 1,7‐ditriazolium PDI **4a**. (a) Proposed modes of chloride binding by *syn/anti* rotamers of PDI **4a**. (b) Variable temperature ^1^H NMR spectra of **4a** before and after the addition of 20 equivalents of chloride in MeCN‐*d*_3_ (400 MHz). (c) UV‐vis spectroscopic titration between host **4a** and chloride guest (PhMe, 10 µM). Arrows indicate anion binding is concomitant with dimer→monomer disassembly (purple→yellow lines). (d) Calculated structure (CREST and DFT) of the homochiral PDI **4a**–BINOL‐phosphate complex (chirality = *M*−). (e) CD spectra of **4a** before (hexafluorophosphate salt) and after the addition of (+/−)‐BINOL‐phosphate in PhMe. (f) Variable temperature ^1^H NMR spectra of **4a** upon its titration with up to five equivalents of (−)‐BINOL‐phosphate in PhMe‐*d*_*8*_ (400 MHz).

Chloride binding by **4a** was further investigated by ^1^H NMR spectroscopy in MeCN‐*d_3_*, with spectra recorded at room and elevated temperatures (Figure 5b, Supporting Information Figures S6‐9 and S6‐10). At 75 °C, the host exhibits a single set of sharp signals and, following titration with up to 20 equivalents of guest (generating > 95% of the 1:2 host–guest complex), the triazolium C–H_d_ protons are shifted to higher ppm (Δ*δ* = 0.9 ppm), which is consistent with their hydrogen bonding to chloride. At room temperature, ^1^H NMR spectroscopy reveals that both triazolium protons H_d_ and H_g_ are split into two sets of signals, indicating that the chloride complex of **4a** also adopts *syn*‐ and *anti*‐rotamers (Figure 5a) and in the same 2:1 ratio as the hexafluorophosphate salt.

We also investigated how the dimer of **4a** responds to the addition of chloride by performing a UV‐vis spectroscopic titration in PhMe (Figure 5c, Supporting Information Figure S4‐4). The change in the PDI absorption spectrum is more drastic than in MeCN, giving rise to a colour change that is visible to the naked eye (Figure 5c). Specifically, chloride binding increases the vibronic ratio (A_0‐0/0‐1_) of the S_0_→S_1_ band, leading to the complete disassembly of the **4a** dimer into monomers (Supporting Information Figure S4‐5). This is because, analogous to solvents such as MeCN and MeOH, chloride competes with 1,7‐ditriazolium PDI self‐assembly for intermolecular hydrogen bonding. Interestingly, despite the enthalpic penalty associated with disrupting the intermolecular dimer, chloride binding is over 30 times stronger in PhMe than MeCN, most likely due to the favorable entropy change arising from disassembly and the lower polarity of PhMe.

Having established chloride sensing by **4a**, we hypothesized that the dynamic *M*/*P* chirality of a 1,7‐ditriazolium PDI might be exploited for the (chir)optical sensing of chiral anions, via a mechanism in which the chiral guest induces an enantiomeric excess in the racemic PDI host. Considering the non‐covalent interactions in the **4a** dimer, we chose to examine the binding of three distinct chiral anions (Table 1), capable of either hydrogen bonding (i.e. (+)‐10‐camphorsulfonate), π–π stacking interactions (Δ‐TRISPHAT) or both ((+/−)‐BINOL‐phosphate). UV‐vis spectroscopic titrations showed that these chiral anions also trigger dimer disassembly and that they are bound with *K*_1_ values at least of the same order of magnitude as chloride in PhMe (Table 1, Supporting Information Figures S4‐6 to S4‐10). Moreover, circular dichroism (CD) spectroscopic titrations revealed that both the aromatic chiral anions (Δ‐TRISPHAT and (+/−)‐BINOL‐phosphate) induce a CD spectrum from the PDI chromophore in **4a**, i.e., in the PDI‐only region of the spectrum (*λ* > 300 nm).^[^ ^82^, ^83^ ^]^ This occurs because the enantiopure anion (e.g., (−)‐BINOL‐phosphate) binds preferentially to one of the *M*/*P* enantiomers of PDI **4a**, to favor the formation of one diastereomeric complex (e.g., chirality = *M*−), over the other (*P*−). As expected, the enantiomers of the (+/−)‐BINOL‐phosphate anion give rise to mirror image CD spectra from the PDI chromophore (Figure 5e).

We used CREST to identify the lowest energy structure of the **4a**–(−)‐BINOL‐phosphate complex and optimized this by DFT (Figure 5d, Supporting Information Figure S5‐12). By predicting the CD spectrum of this calculated structure (Supporting Information Figure S5‐13), the PDI chirality is assigned as *P* and *M* with (+)‐ and (−)‐BINOL‐phosphate respectively. This demonstrates that the 1,7‐ditriazolium PDI favors the formation of homochiral (*P+*/*M*−) over heterochiral (*P−*/*M*+) non‐covalent complexes with this chiral anion, which is the opposite diastereoselectivity to that we observed in the synthesis of a covalent PDI–BINOL macrocycle under kinetic control.^[^ ^84^ ^]^

Notably, the (+)‐10‐camphorsulfonate binds stronger than the other two chiral anions (Table 1), yet the absence of a CD spectrum with the **4a**–(+)‐10‐camphorsulfonate complex (Supporting Information Figure S4‐7) points to the importance of aromatic interactions (e.g., π–π) between the PDI host and anion guest in affording *enantio*selective recognition. Of the two aromatic chiral anions, the strongest CD spectrum is induced with (+/−)‐BINOL‐phosphate which, in contrast to Δ‐TRISPHAT, is capable of both hydrogen bonding and π–π interactions, as seen in the calculated structure of the complex (Figure 5d, Supporting Information Figure S5‐12). The formation of π–π interactions is aided by the complementary shape of the helical π‐surfaces of the host and guest, i.e., between the twisted perylene and binaphthol aromatic groups respectively (Figure 5d). Furthermore, out of all the anions tested, (+/−)‐BINOL‐phosphate binding is unique in eliciting new absorptions at higher wavelengths (*λ* > 575 nm),^[^ ^38^ ^]^ indicative of charge transfer between the π‐electron rich anion guest and the π‐electron deficient PDI host. This charge transfer band is also evident from the change in the sign of the CD spectrum at *λ* = 600 nm (Figure 5e).

Finally, we used (variable temperature) ^1^H NMR spectroscopy to investigate (−)‐BINOL‐phosphate binding by **4a** in PhMe (Figure 5f, Supporting Information Figures S6‐10 and S6‐11). Here, up to five equivalents of the chiral anion were titrated into a solution of **4a** to generate > 90% of the 1:2 host–guest complex (Figure 5f). The elevated temperature spectra show that (−)‐BINOL‐phosphate binding causes the triazolium signal H_d_ to move to higher ppm (Δ*δ* = 0.79 ppm), consistent with hydrogen bonding to the anion. As well as some broadening, the PDI signals (H_a–c_) also undergo significant shifting (Δ*δ* = 0.33 ppm), more so than with chloride binding (Δ*δ* = 0.17 ppm), consistent with new π–π interactions between the PDI host and aromatic guest. Upon cooling to room temperature, the ^1^H NMR spectrum of the (−)‐BINOL‐phosphate complex of **4a** becomes increasingly complex, with the signal containing two overlapping triazolium H_d_ and PDI H_b_ proton peaks splitting into multiple peaks. The more acidic H_d_ protons readily undergo hydrogen–deuterium exchange (Supporting Information), which enabled us to assign this region of the spectrum. Unlike chloride (Figure 2b), (−)‐BINOL‐phosphate causes the PDI protons H_b_ to be split into two signals due to the formation of the two homochiral (*M*−) and heterochiral (*P*−) complexes.^[^ ^85^ ^]^ The relative integration of these peaks allows us to estimate a 20% diastereomeric excess for the homochiral complex (Supporting Information Figure S6‐13). This value is of the order of magnitude expected from the molar extinction of the CD spectrum of **4a** (Figure 5e) upon comparison with related enantiopure PDIs.^[^ ^31^, ^84^ ^]^

## 3 Summary and Conclusions

This work describes the preparation of a family of novel PDI and CDI dyes to investigate the impact of chromophore planarity, charge, regioisomerism, and chirality on the dye's self‐assembly and anion recognition properties. We find that the methylation of neutral 1,7‐ or 1,6‐ditriazole PDIs (**3a,b**) to generate cationic ditriazolium PDIs (**4a,b**) leads to pronounced *syn*/*anti* rotational isomerism, as well as *M*/*P* stereoisomerism of core‐twisted PDIs. While both rotamers and stereoisomers are dynamic at room temperature, the former has a higher interconversion barrier (Δ*G* ^‡^ = 61 vs. 36 kJ mol⁻^1^), with the 1,7‐ditriazolium PDI (**4a**) favoring the *syn*‐rotamer and the 1,6‐regioisomer (**4b**) the *anti*‐rotamer.

Unlike many PDI derivatives which are insoluble or aggregate in polar/protic solvents,^[^ ^75^ ^]^ our ditriazolium PDIs are monomeric in MeCN, MeOH and H_2_O. Only the 1,7 regioisomer (**4a**) undergoes self‐assembly and only in less polar solvents, forming stable dimers in CHCl_3_ or PhMe (*K*_dim_ ≥ 10^5^). This behavior is significant considering the greater electrostatic repulsion between the cationic dyes and that, relative to planar chromophores (e.g., CDI **6**, K_a_ = 10^5^), core‐twisting often weakens PDI aggregation. Instead, methylation facilitates **4a** self‐assembly because it increases the strength of intermolecular hydrogen bonding involving the more acidic triazolium proton, H_d_. As such, *K*_dim_ is an order of magnitude larger than previous core‐twisted PDI dimers that use only π–π interactions.^[^ ^11^, ^12^ ^]^ Importantly, dimerization in solution requires at least one of the PDI monomers to be a *syn*‐rotamer of the 1,7 regioisomer, since this is the only combination that can donate two H_d_‐based hydrogen bonds from the same unhindered face of the perylene core. This complementarity means that *syn*–*syn* dimers are stronger than *syn*–*anti* analogues. The dimers of **4a** favour homochiral (*MM*/*PP*) and co‐facial stacking of the PDI's π‐surfaces, leading to H‐type excitonic coupling. This complements the slipped‐stacked (J‐type) assemblies of core‐twisted PDIs that use the imide positions for intermolecular hydrogen bonding^[^ ^19^ ^]^ and favor heterochiral over homochiral π–π self‐assembly.^[^ ^23^ ^]^ The barrier to stereoisomer interconversion (Δ*G* ^‡^) in the PDI dimer is double that of the **4a** monomer, indicating that self‐assembly may provide a route to core‐twisted PDIs with persistent chirality.

Due to their hydrogen bonding properties, the ditriazolium PDIs **4a,b** are receptors and (chir)optical sensors for chloride and (chiral) oxoanions. Colorimetric anion sensing is best exhibited by the **4a** dimer due to its distinct photophysics relative to the monomer. Here, anion binding is concomitant with dimer disassembly since the guest outcompetes the self‐recognition of the host, a mechanism that underpins recent aggregate‐based anion receptor^[^ ^86^, ^87^, ^88^ ^]^ and sensor^[^ ^89^, ^90^ ^]^ designs. Moreover, **4a** affords chiroptical sensing of chiral aromatic anions, since preferential binding to one *M*/*P* enantiomer triggers **4a** deracemization, thereby inducing a CD signal from the PDI chromophore. Enantioselective recognition (de ∼ 20%) was best seen with (+/−)‐BINOL‐phosphate due to chiral (i.e., homochiral > heterochiral) and electronic (π‐acceptor–π‐donor) complementarity in the host–guest complex. This primes the development of PDI‐based chiroptical sensors for other important substrates, while the non‐covalent imprinting of (homo)chirality provides a promising tool to template the stereoselective synthesis of chiral materials.

## Supporting Information

Synthetic procedures, characterization data, additional spectra, titration data, single crystal diffraction data, DFT optimized structures, and experimental and computational methods can be found in the Supporting Information.

Deposition Numbers https://www.ccdc.cam.ac.uk/services/structures?id = https://doi.org/10.1002/chem.202501270 2 429 095 (for **3a**), 2 429 097 (for **4a**), 2 429 096 (for (−)‐BINOL‐phosphate)) and 2 429 098 (for (+)‐BINOL‐phosphate) contain the supplementary crystallographic data for this paper. These data are provided free of charge by the joint Cambridge Crystallographic Data Centre and Fachinformationszentrum Karlsruhe http://www.ccdc.cam.ac.uk/structures Access Structures service.

## Conflict of Interests

The authors declare no conflicts of interest.

## Supporting information



Supporting Information

Supporting Information

## Data Availability

The data that support the findings of this study are available in the supplementary material of this article.
