# Supplementary material for: Core‐Twisted, Cationic Perylene Diimides; Homochiral Dimerization and Chiroptical Anion Sensing
Source: Chemistry. 2025 May 2;31(31):e202501270. doi: 10.1002/chem.202501270 (PMC12133647; doi:10.1002/chem.202501270)
Supplement: Supplementary file 1 — Supporting Information [file CHEM-31-e202501270-s001.docx]

**Supporting Information**

**Core-Twisted, Cationic Perylene Diimides; Homochiral Dimerisation and Chiroptical Anion Sensing**

Denis Hartmann,^1^ Jamie S. Hillis,^1^ Lucy E. Walker,^1^ Timothy A. Barendt^1,*^

^1^School of Chemistry, University of Birmingham, Edgbaston, Birmingham B15 2TT, United Kingdom

**Contents:**

[1) Synthesis 2](#_Toc195522371)

[2) Photophysical Properties 17](#_Toc195522372)

[3) Aggregation Behaviour 21](#_Toc195522373)

[4) Anion Binding 28](#_Toc195522374)

[5) Computational Chemistry 34](#_Toc195522375)

[6) NMR Spectra 46](#_Toc195522376)

[7) Single Crystal X-Ray Diffraction 87](#_Toc195522377)

[8) Mass Spectra 96](#_Toc195522378)

[9) References 108](#_Toc195522379)

# Synthesis

### General Synthesis

Reagents were purchased from commercial sources (Merck, Acros Organics, Fluorochem and Alfa Aesar) and used without further purification. Solvents were used as supplied (analytical/HPLC-grade from Fisher or Sigma-Aldrich). If required, solvents were dried over molecular sieves (3 Å) overnight before use and stored under N_2_ atmosphere. Petroleum ether (PE) over a boiling point range of 40–60 °C was used. Eluent mixtures are reported in volume:volume or %vol. Column chromatography was carried out using Merck Silica Gel 60 Å, 230-400 mesh, 40-63 µm particle size. TLC was carried out on Merck silica gel 60 F254 Al plates. Preparative TLC was performed using 20 × 20 cm plates with 1 cm silica thickness. NMR spectroscopy measurements were recorded using a Bruker AVIII 300, AVIII 400, AV Neo 400 or AV Neo 500 instrument and peaks were referenced to the residual solvent peak. Electrospray Ionisation Mass Spectrometry (ESI-MS) measurements were carried out on a **Waters Xevo G2-XS TOF Mass Spectrometer.**

### 1,7- and 1,6-Br_2_-C5-PDI 1a/b

Di-brominated-C5-PDI was prepared according to literature procedures.^[1]^ The resulting isomers were subsequently separated by preparative HPLC on a COSMOSIL Buckyprep column (28 mm x 250 mm) using a gradient of 25-100% DCM/hexane over 18 minutes, followed by an isocratic step of 100% DCM for 2 minutes, followed by isocratic re-equilibration with 25% DCM/hexane, with a flowrate of 10 mL/min throughout. This yielded the desired, regiopure 1,7- and 1,6-dibromo-PDIs in a ~4:1 ratio.

1,7-Isomer (**1a**): **^1^H-NMR** (400 MHz, CDCl_3_) δ/ppm 9.49 (d, *J* = 8.2 Hz, 2H), 8.91 (s, 2H), 8.69 (d, *J* = 8.1 Hz, 2H), 5.05 (tt, *J* = 9.7, 5.8 Hz, 2H), 2.41 – 2.11 (m, 4H), 2.08 – 1.80 (m, 4H), 0.92 (t, *J* = 7.4 Hz, 12H).

1,6-Isomer (**1b**): **^1^H-NMR** (400 MHz, CDCl_3_) δ/ppm 9.50 (d, *J* = 8.1 Hz, 2H), 8.91 (s, 2H), 8.70 (d, *J* = 8.1 Hz, 2H), 5.05 (dtt, *J* = 21.4, 9.7, 5.8 Hz, 2H), 2.25 (dddt, *J* = 21.3, 14.9, 9.7, 7.5 Hz, 4H), 2.08 – 1.83 (m, 4H), 0.92 (dt, *J* = 8.5, 7.4 Hz, 12H).

**HRMS** (ESI^+^) of mixture found 687.0533, [M+H]^+^ needs 687.0489.

Data in accordance with the literature.^[1]^


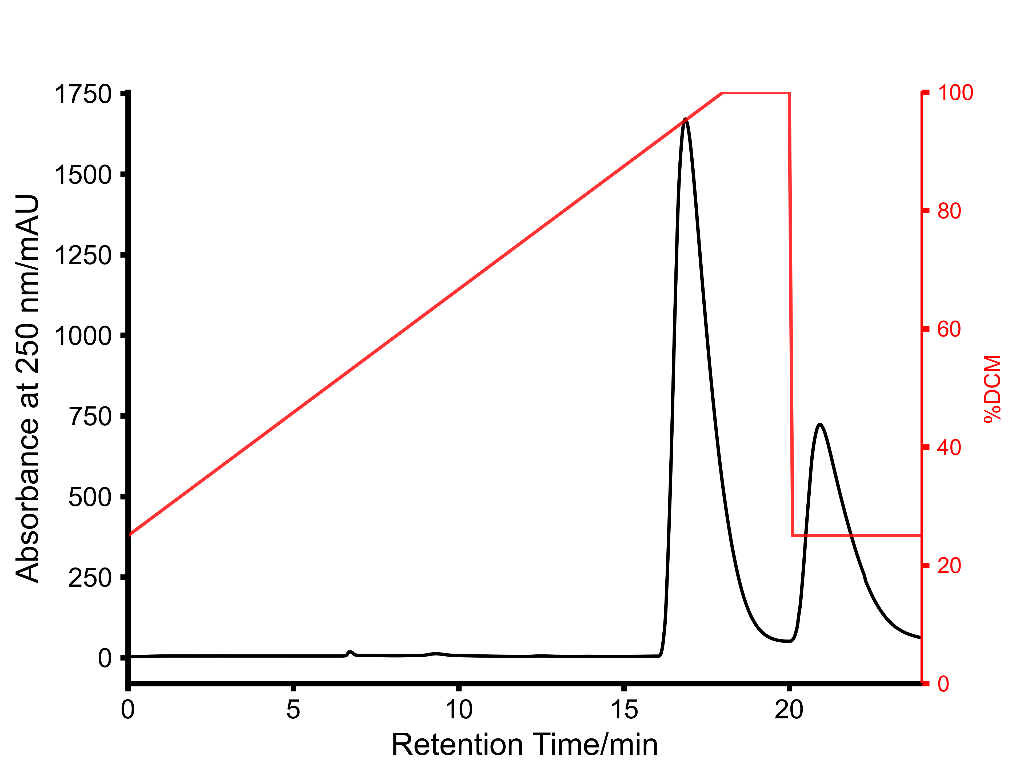


**Supplementary Figure 1‑1**: HPLC trace of 1,7- and 1,6-dibromo-C5-PDI separation. The 1,7-isomer **1a** elutes first.

### 1,7-di(Trimethylsilylacetylene)-C5-PDI 2a

Following a literature procedure:^[2]^ To a round-bottom flask under nitrogen atmosphere was added 1,7-dibromo-C5-PDI **1a** (400 mg, 0.581 mmol), Pd(PPh_3_)_2_Cl_2_ (20 mg,
5 mol%), CuI (10 mg, 10 mol%) and 10 mL of a 1:1 Mixture of NEt_3_:PhMe. The reaction was subsequently degassed by sparging with N_2_ for 30 minutes. Then, trimethylsilylacetylene (0.5 mL, 3.5 mmol, 6 eq.) was added and the reaction stirred at room temperature for 48 hours. The solvent was then removed *in vacuo* and the resulting residue purified by flash column chromatography (SiO_2_, 4:1 PE:CH_2_Cl_2_) to yield the desired compound as a purple solid (420 mg, 0.580 mmol, 99%).

**^1^H NMR** (400 MHz, CDCl_3_) δ/ppm 10.22 (d, *J* = 8.3 Hz, 2H), 8.82 (s, 2H), 8.65 (d,
*J* = 8.3 Hz, 2H), 5.13 – 5.01 (m, 2H), 2.36 – 2.20 (m, 4H), 2.01 – 1.86 (m, 4H), 0.92 (t, *J* = 7.4 Hz, 12H), 0.39 (s, 18H).

**HRMS** (ESI^+^) found 723.3110, [M+H]^+^ requires 723.3069.

Data in accordance with previous reports.^[2]^

### 1,6-di(Trimethylsilylacetylene)-C5-PDI 2b

Following a literature procedure:^[2]^ To a round bottom flask under Nitrogen atmosphere was added 1,6-dibromo-C5-PDI **1b** (500 mg, 726.4 µmol), Pd(PPh_3_)Cl_2_ (25.5 mg, 36.32 µmol, 5 mol%) and CuI (14 mg, 72.6 µmol, 10 mol%). To this was added 9 mL each of PhMe and NEt_3_ and the mixture degassed by bubbling with N_2_ for 30 minutes. To this is then added trimethylsilylacetylene (620 µL, 4.36 mmol, 6 eq.) and the reaction left stirring for 48 hours. The solvent was subsequently removed in vacuo and the resulting residue purified by flash column chromatography (SiO_2_, 3:2
PE_40-60_:CH_2_Cl_2_) to yield a pinkish-red solid as the desired compound (521 mg,
720.6 µmol, 99%).

**^1^H-NMR** (400 MHz, CDCl_3_) δ/ppm 10.18 (d, *J* = 8.2 Hz, 2H, **b**), 8.79 (s, 2H, **a**), 8.64 (d, *J* = 8.2 Hz, 2H, **c**), 5.07 (dddd, *J* = 16.7, 15.4, 9.6, 5.8 Hz, 2H, **d**), 2.37 – 2.18 (m, 4H, **e’**), 2.03 – 1.85 (m, 4H, **e’’**), 0.93 (dt, *J* = 10.3, 7.4 Hz, 12H, **f**), 0.39 (s, 18H, **g**).

**^1^H-NMR** (400 MHz, CDCl_3_) δ/ppm 164.55, 139.00, 135.22, 133.75, 130.60, 128.79, 128.23, 127.85, 127.72, 127.52, 124.12, 122.31, 120.77, 106.27 & 106.14, 58.28 & 58.10, 25.49, 25.35, 11.75, 11.67, 0.00.

**HRMS** (ESI^+^) found 723.3110, [M+H]^+^ requires 723.3069.

### 1,7-di(Octyltriazole)-C5-PDI 3a

To a round-bottom flask eqipped with a stirrer bar was added 1,7-di(trimethylsilylacetylene)-C5-PDI **2a** (125 mg, 173 µmol). To this was then added CH_2_Cl_2_ (25 mL) and MeOH (5 mL) followed by TBAF (1 M in THF, 0.2 mL). The reaction was left stirring until TLC showed complete conversion to the di-deprotected species. The resulting solution was diluted with more CH_2_Cl_2_ (25 mL), washed with H_2_O (2 x 50 mL), brine (2x 50 mL), the organic phase dried with MgSO_4_ and reduced *in vacuo*. This residue was then dissolved in CH_2_Cl_2_ (20 mL). To this was then added *n*-octyl-azide (113 mg, 0.727 mmol, 4 eq.) and the solution degassed by bubbling with N_2_ for 20 minutes. Then, Cu(MeCN)_4_PF_6_ (13 mg, 20 mol%) and TBTA (18 mg,
20 mol%) were added, the reaction bubbled with N_2_ for a further 10 minutes and left stirring overnight. The resulting dark purple solution was reduced *in vacuo* and the residue purified by flash column chromatography (SiO_2_, 2-4% acetone/CH_2_Cl_2_) to yield a purple solid as the desired product (118 mg, 133 µmol, 77%).

**^1^H NMR** (400 MHz, CDCl_3_) δ/ppm 8.68 (s, 2H, **a**), 8.23 (d, J = 8.1 Hz, 2H, **b**), 7.93 (d, J = 8.1 Hz, 2H, **c**), 7.75 (s, 2H, **d**), 5.03 (tt, 2H, **e**), 4.48 (t, J = 7.2 Hz, 4H, **h**), 2.23 (m, 4H, **f’**), 2.00 (t, J = 7.2 Hz, 4H, **i**), 1.95 – 1.83 (m, 4H, **f’’**), 1.45 – 1.21 (m, 22H, **j**), 0.88 (m, 18H_,_ **g** & **k**).

**^13^C NMR** (101 MHz, CDCl_3_) δ/ppm 164.04, 147.82, 135.10, 134.61, 133.44, 129.85, 129.11, 129.09, 129.07, 128.43, 122.72, 121.64, 57.63, 50.83, 31.73, 30.37, 29.70, 29.06, 28.93, 26.50, 24.99, 22.59, 14.08, 11.25.

**HRMS** (ESI^+^) found 889.5129, [M+H]^+^ requires 889.5124.

### 1,6-bis(Octyltriazole)-C5-PDI 3b

To a round-bottom flask eqipped with a stirrer bar was added 1,6-di(trimethylsilylacetylene)-C5-PDI **2b** (226 mg, 312.6 µmol). To this was then added CH_2_Cl_2_ (25 mL) and MeOH (5 mL) following by TBAF (1 M in THF, 0.3 mL, 10 eq.). The reaction was left stirring until TLC showed complete conversion to the di-deprotected species. The resulting solution was diluted with more CH_2_Cl_2_ (25 mL), washed with H_2_O (2x 50 mL), brine (2x 50 mL), the organic phase dried with MgSO_4_ and reduced *in vacuo*. This residue was then dissolved in CH_2_Cl_2_ (30 mL). To this was then added *n*-octyl-azide (970 mg, 6.25 mmol, 20 eq.) and the solution degassed by bubbling with N_2_ for 20 minutes. Then, Cu(MeCN)_4_PF_6_ (23.3 mg, 62.51 µmol, 0.2 eq.) and TBTA (33.2 mg, 62.51 µmol, 0.2 eq) were added, the reaction bubbled with N_2_ for a further 10 minutes, the flask stoppered and left stirring overnight. The resulting dark purple solution was reduced *in vacuo* and the residue purified by flash column chromatography (SiO_2_,2% acetone/CH_2_Cl_2_) to yield a purple solid as the desired product (227 mg, 255 µmol, 82%).

**^1^H-NMR** (400 MHz, CDCl_3_) δ/ppm 8.64 (s, 2H, **a**), 8.19 (d, J = 8.1 Hz, 2H, **c**), 7.86 (d, J = 8.1 Hz, 2H, **b**), 7.68 (s, 2H, **d**), 5.03 (dtt, J = 19.6, 9.7, 5.8 Hz, 2H, **e**), 4.48 (t, J = 7.2 Hz, 4H, **f**), 2.23 (dddd, J = 17.4, 13.9, 9.8, 7.3 Hz, 4H, **g’**), 2.05 – 1.95 (m, 4H, **h**), 1.89 (dddd, J = 16.8, 13.6, 7.5, 5.9 Hz, 4H, **g’’**), 1.47 – 1.22 (m, 20H, **i**), 0.95 – 0.82 (m, 18H, **j** & **k**).

**^13^C-NMR** (101 MHz, CDCl_3_) δ/ppm 164.11, 148.15, 135.74, 134.27, 133.70, 130.12, 129.72, 129.37, 128.86, 128.73, 128.68, 128.41, 122.70, 121.58, 57.90, 57.62, 50.94, 31.86, 30.50, 29.17, 29.04, 26.61, 25.12, 25.10, 22.71, 14.19, 11.37.

**HRMS** (ESI^+^) found 889.5129, [M+H]^+^ requires 889.5124.

### 1,7-di(Octyl-methyltriazolium)-C5-PDI Iodide S1

1,7-di(Octyltriazole)-C5-PDI **3a** (86.5 mg, 0.097 mmol) was dissolved in neat iodomethane (7 mL) in an oven-dried pressure tube (rated to 10 bar). The reaction was heated at 80 °C for three days until the reaction showed completion as monitored by TLC (5 % MeOH in CHCl_3_). The reaction mixture was cooled to room temperature and the MeI was removed under reduced pressure. The crude reaction was purified by preparative TLC (2 % MeOH in CHCl_3_) over three runs to obtain a dark red solid
(89.8 mg, 0.076 mmol, 79%).

**^1^H NMR** (300 MHz, MeCN-*d*_3_) δ/ppm 9.05 (s, 2H), 8.63 (s, 2H), 8.55 (d, *J* = 8.1 Hz 2H), 7.80 (d, *J* = 8.1 Hz, 2H), 4.99 (m, 2H), 4.78 (s, 4H), 3.74 (s, 8H), 2.31 – 2.18 (m, 2H), 1.89 (m, 3H), 1.40 (m, 24H), 1.00 – 0.82 (m, 18H).

**^13^C NMR** (101 MHz, MeCN-*d*_3_) δ/ppm 164.30, 143.47, 135.67, 133.35, 131.87, 130.56, 128.90, 124.76, 120.72, 58.96, 56.08, 55.33, 46.82, 39.69, 32.52, 29.79, 29.62, 29.48, 26.80, 25.70, 23.36, 14.40, 11.73, 8.92.

**HRMS** (ESI^−^) m/z calculated [M-I^-^] 459.2760, found 459.2746.

**HRMS** (ESI^−^) m/z calculated [I^-^] 126.9045, found 126.9042.

### 1,7-di(Octyl-methyltriazolium)-C5-PDI PF_6_ 4a

di-triazolium PDI **S1** (39.9 mg, 34 µmol) was dissolved in CHCl_3_ (5 mL). To this, a
0.1 M solution of NH_4_PF_6_ (5 mL) was added and the solution was stirred vigorously for
5 minutes. The organic layer was removed and added to another 0.1 M solution of NH_4_PF_6_ (5 mL). This process was repeated for a total of six washes. The organic layer was then removed and washed with H_2_O (2 x 5 mL) and dried over Mg_2_SO_4_ to give a bright red solid (40 mg, 33.1 µmol, 97%)

**^1^H NMR** (400 MHz, 348 K, MeCN-*d*_3_) δ/ppm 8.78 (s, 2H, **d**), 8.66 (s, 2H, **a**), 8.51 (d, *J* = 8.1 Hz, 2H, **b**), 7.72 (d, *J* = 8.0 Hz, 2H, **c**), 5.02 (tt, *J* = 9.3, 5.8 Hz, 2H, **f**), 4.76 (t, *J* = 7.2 Hz, 4H, **e**), 3.84 (s, 6H, **g**), 2.37 – 2.12 (m, 6H, **h**’ & **j**), 2.04 – 1.89 (m, 2H, **h’’**)*, 1.65 – 1.26 (m, 15H, **j**), 1.05 – 0.76 (m, 18H, **i** & **k**).

**^13^C NMR** (101 MHz, 298 K, MeCN-*d*_3_) δ/ppm 164.40, 143.91, 135.79, 133.73, 133.21, 131.54, 130.81, 129.91, 129.06, 125.20, 120.80, 58.86, 55.74, 39.10, 32.53, 30.36, 29.81, 29.60, 26.80, 25.75, 23.38, 14.42, 11.59.

**^19^F NMR** (377 MHz, MeCN-*d*_3_) δ/ppm -72.97 (d, J = 706.8 Hz).

**HRMS** (ESI^–^) m/z calculated [M-2PF_6_^-^] 459.2760, found 459.2751.

**HRMS** (ESI^–^) m/z calculated [PF_6_^-^] 144.9642, found 144.9642

*Signal should read as at least 4 protons, but peak is obstructed by solvent and therefore cannot be accurately integrated.

**HRMS** (ESI^+^) found 459.2770, [M]^2+^ requires 459.2755.

### 1,6-di(Octylmethyltriazolium)-C5-PDI PF_6_ 4b

To a flame-dried flask equipped with a stirrer bar under nitrogen atmosphere,
1,6-di(octyltriazole)-C5-PDI **3b** (50 mg, 56.23 µmol) was dissolved in neat iodomethane (3 mL), and the reaction protected from light. The reaction mixture was left stirring for 3 weeks, after which it has turned a deep purple colour. The solvent was removed and the residue dissolved in 25 mL of CH_2_Cl_2_. The solution was washed with a saturated solution of NH_4_PF_6_ (5x 20 mL), after which the solution turned from dark purple to a burnt orange colour. The organic phase was then dried (MgSO_4_), filtered and reduced *in vacuo*. The resulting residue was purified by preparative thin layer chromatography (SiO_2_, 2% MeOH/CH_2_Cl_2_) to yield the desired compound as a dark orange to red solid (35 mg, 28.95 µmol, 51%).

**^1^H NMR** (400 MHz, 348 K, MeCN-*d_3_*) δ/ppm 8.76 (s, 2H, **d**), 8.72 (s, 2H, **a**), 8.44 (d, *J* = 8.1 Hz, 2H, **c**), 7.70 (d, *J* = 8.1 Hz, 2H, **b**), 5.02 (dtt, *J* = 18.6, 9.4, 5.8 Hz, 2H, **e**), 4.75 (t, *J* = 7.2 Hz, 4H, **g**), 3.75 (s, 6H, **f**), 2.31 – 2.13 (m, 8H, **h’** & **g**), 1.97 (m, 4H, **h’’**), 1.61 – 1.27 (m, 24H, **i**), 1.02 – 0.87 (m, 18H, **j** & **k**).

**^13^C NMR** (101 MHz, 298 K, MeCN-*d_3_*) δ/ppm 164.28, 143.83, 137.11, 132.27, 131.78, 129.94, 129.84, 129.46, 128.08, 125.83, 124.40, 121.18, 59.02, 58.58, 55.68, 40.96, 38.96, 32.46, 30.30, 29.75, 29.53, 26.72, 25.71, 25.64, 23.32, 14.35, 11.55, 11.52.

**HRMS** (ESI^+^) found 459.2795, [M]^2+^ requires 459.2755.

### 1,7-di(Octyltriazole)-C5-CDI S2

To a scintillation vial equipped with a stirrer bar was added 1,7-di(octyltriazole)-C5-PDI **3a** (28.5 mg, 32 µmol) and dissolved in 20 mL of CHCl_3_. The vial was then placed into a Photocube reactor (ThalesNano), stirred at 1000 RPM and illuminated for
4 hours with 525 nm LEDs. The resulting greenish solution was reduced *in vacuo* and the residue purified by column chromatography (SiO_2_, CH_2_Cl_2_) to yield a poorly soluble orange solid as the desired compound (19.2 mg, 22 µmol, 68%).

**^1^H NMR** (400 MHz, TCE-*d_2_,* 298 K) δ/ppm 10.71 (s, 2H, **a**), 10.17 (s, 2H, **b**), 5.61 (t, *J* = 7.1 Hz, 4H, **d**), 5.33 (tt, *J* = 9.2, 6.1 Hz, 2H, **c**), 2.55 – 2.40 (m, 8H, **d** & **e’**), 2.19 (dt, *J* = 13.9, 6.9 Hz, 2H, **e’’**), 1.71 (p, *J* = 7.5 Hz, 4H, **g**), 1.53 (p, *J* = 7.0 Hz, 4H, **h**), 1.43 – 1.26 (m, 16H, **i**), 1.11 (t, *J* = 7.4 Hz, 12H), 0.88 (m, 6H, **j**).

**^13^C NMR** (400 MHz, TCE-*d_2_*, 373 K) δ/ppm 164.17, 164.00, 142.05, 128.48, 125.79, 123.60, 123.48, 123.42, 123.33, 123.00, 122.85, 122.45, 121.78, 117.65, 58.70, 51.75, 31.44, 29.55, 28.85, 28.80, 26.60, 25.24, 22.27, 13.67, 11.40. Note: ^13^C spectrum had to be recorded at 373 K due to poor solubility.

**HRMS** (ESI^+^) found 885.4751, [M+H]^+^ requires 885.4811.

### 1,7-Br_2_ -C11-PDI S3

Prepared according to literature procedures.^[3]^ The isomers were subsequently separated by preparative HPLC on a COSMOSIL Buckyprep column (28 mm x
250 mm) using a gradient of 25-60% DCM/hexane over 20 minutes followed by isocratic re-equilibration with 25% DCM/hexane for 2 minutes, with a flowrate of
10 mL/min throughout. This yielded the desired, regiopure 1,7- and 1,6-dibromo-PDIs in a ~4:1 ratio.


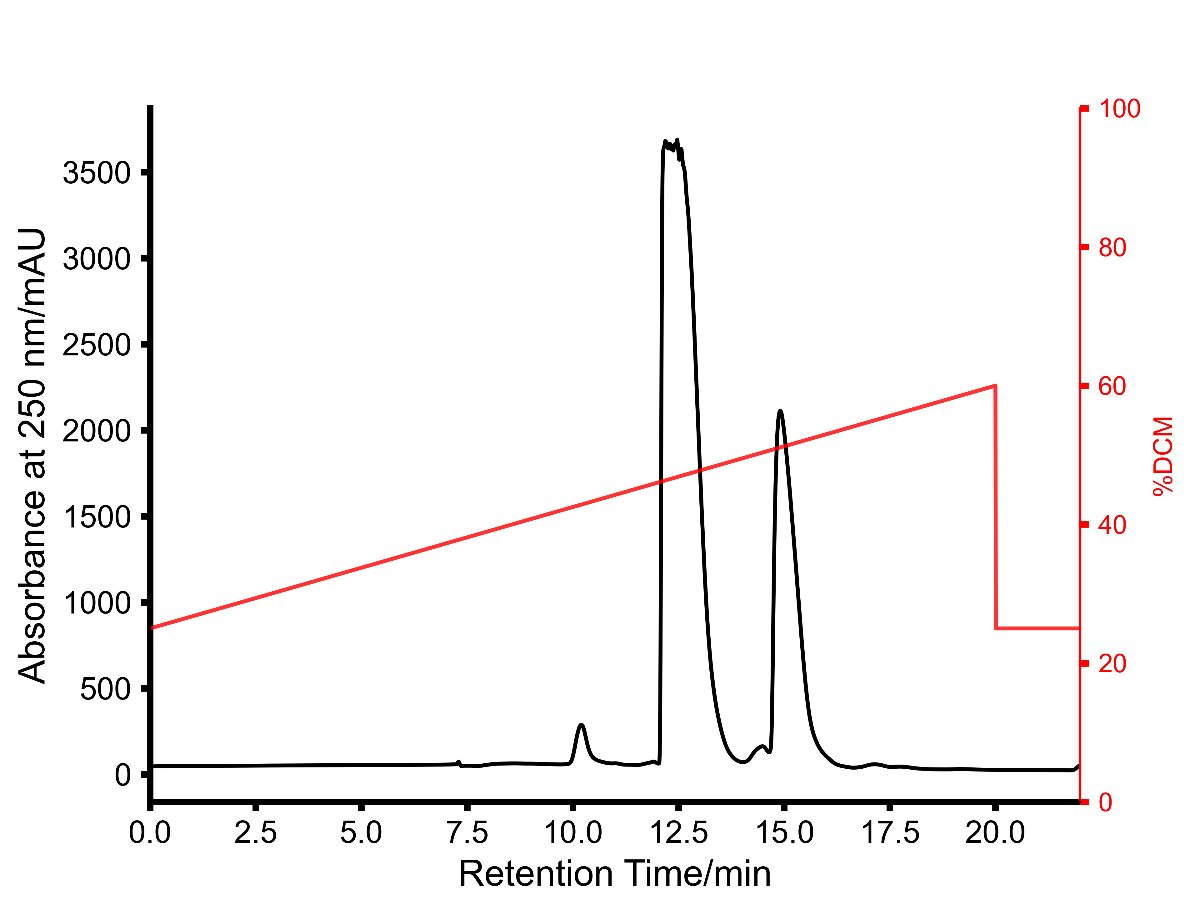


**Supplementary Figure 1‑2:** Separation of 1,7- and 1,6-regioisomers of dibromo-C11-PDI. The 1,7-isomer elutes first at ~12.5 min.

**^1^H NMR** (300 MHz, CDCl_3_) δ/ppm 9.49 (d, *J* = 8.2 Hz, 2H), 8.91 (s, 2H), 8.69 (s, 2H), 5.25 – 5.09 (m, 2H), 2.23 (dt, *J* = 13.4, 9.3 Hz, 4H), 1.91 – 1.74 (m, 1H), 1.39 – 1.17 (m, 27H), 0.89 – 0.78 (m, 12H).

**MS** (ESI^+^) found 855.23 [M+H]^+^.

Data in accordance with literature.^[2]^

### 1,7-di(Trimethylsilylacetylene)-C11-PDI S4

The title compound was prepared using regiopure 1,7-dibromo-C11-PDI following a literature procedure.^[3]^ In a round-bottom flask equipped with a stirrer bar was added 1,7-dibromo-C11-PDI (500 mg, 0.584 mmol), Pd(PPh_3_)_2_Cl_2_ (20.5 mg, 5 mol%), CuI (11 mg, 10 mol%) and 10 mL of a 1:1 mixture of NEt_3_:PhMe. The reaction was subsequently degassed by sparging with N_2_ for 30 minutes. Then, trimethylsilylacetylene (0.5 mL, 3.5 mmol, 6 eq.) was added and the reaction stirred at room temperature for 48 hours. The solvent was then removed *in vacuo* and the resulting residue purified by flash column chromatography (SiO_2_, 4:1 PE:CH_2_Cl_2_) to yield the desired compound as a purple solid (520 mg, 0.584 mmol, quant.).

**^1^H-NMR** (300 MHz, CDCl_3_) δ/ppm 10.23 (d, *J* = 8.3 Hz, 2H), 8.83 (s, 2H), 8.69 – 8.61 (m, 2H), 5.19 (tt, *J* = 9.9, 5.5 Hz, 2H), 2.26 (dt, *J* = 13.7, 9.4 Hz, 4H), 1.91 – 1.78 (m, 4H), 1.35 – 1.20 (m, 34H), 0.83 (q, *J* = 5.0 Hz, 15H), 0.39 (s, 18H).

**LRMS** (ESI^+^) found 891.48 [M+H]^+^.

Data in accordance with the literature.^[3]^

### 1,7-di(Octyltriazole)-C11-PDI 5

To a round-bottom flask equipped with a stirrer bar was added 1,7-di(trimethylsilylacetylene)-C11-PDI **S4** (212 mg, 0.238 mmol) and dissolved in 30 mL of a 2:1 mixture of CH_2_Cl_2_:MeOH. To this was added K_2_CO_3_ (118 mg, 0.857 mmol,
3.6 eq.) and the mixture stirred for 15 minutes, after which TLC (2:1 PE_40-60_:CH_2_Cl_2_) indicated full deprotection. The solution was diluted with 20 mL of CH_2_Cl_2_, and washed with H_2_O (2x 25 mL) and brine (1x 50 mL), dried (MgSO_4_), filtered and reduced *in vacuo*. The crude, deprotected PDI was used straight in the next step without further purification.

To the deprotected PDI was then added *n*-octyl azide (184 mg, 5 eq.) and TBTA
(25 mg, 20 mol%), followed by 20 mL of CH_2_Cl_2_. The reaction mixture was degassed by sparging with N_2_. Then, Cu(MeCN)_4_PF_6_ (18 mg, 20 mol%) was added and the mixture left stirring for 48 hours. The solvent was subsequently removed and the residue purified by flash column chromatography (SiO_2_, 2% acetone/CH_2_Cl_2_) to yield a purple solid as the desired compound (169 mg, 0.160 mmol, 57%).

**^1^H NMR** (400 MHz, CDCl_3_) δ/ppm 8.67 (d, *J* = 16.0 Hz, 2H, **b**), 8.22 (d, *J* = 13.3 Hz, 2H, **a**), 7.93 (d, *J* = 8.1 Hz, 2H, **c**), 7.76 (s, 2H, **d**), 5.15 (s, 2H, **e**), 4.49 (t, *J* = 7.2 Hz, 4H, **f**), 2.22 (dtd, *J* = 14.3, 9.8, 4.0 Hz, 4H, **h’**), 2.00 (p, *J* = 7.6 Hz, 4H, **g**), 1.90 – 1.73 (m, 4H, **h’’**), 1.46 – 1.14 (m, 44H, **i** & **j**), 0.93 – 0.78 (m, 18H, **k** & **l**).

**^13^C NMR** (101 MHz, CDCl_3_) δ/ppm 164.60, 163.48, 147.98, 135.58, 134.74, 133.56, 130.37, 129.61, 129.20, 128.53, 123.22, 122.50, 121.76, 54.78, 50.95, 32.41, 31.85, 30.49, 29.17, 29.04, 26.66, 26.62, 22.71, 22.67, 14.19, 14.16.

**HRMS** (ESI^+^) found 1057.7051, [M+H]^+^ requires 1057.7002.

### 1,7-di(Octyltriazole)-C11-CDI 6

To a scintillation vial equipped with a stirrer bar was added 1,7-di(octyltriazole)-C11-PDI **5** (58 mg, 65.23 µmol) and dissolved in 20 mL of CHCl_3_. The vial was then placed into a Photocube reactor (ThalesNano), stirred at 1000 RPM and illuminated for
8 hours with 525 nm LEDs. The resulting greenish solution was reduced *in vacuo* and the residue purified by column chromatography (SiO_2_, CH_2_Cl_2_) to yield an orange solid as the desired compound (22 mg, 24.86 µmol, 38%).

**^1^H NMR** (400 MHz, CDCl_3_) δ/ppm 10.40 (s, 2H, **a**), 9.84 (s, 2H, **b**), 5.54 (t, *J* = 7.4 Hz, 4H, **d**), 5.43 (s, 2H, **c**), 2.51 (dtt, *J* = 14.3, 9.1, 4.2 Hz, 4H, **f’**), 2.40 (p, *J* = 7.4 Hz, 4H, **e**), 2.25 – 2.12 (m, 4H, **f’’**), 1.70 (p, *J* = 7.2 Hz, 4H, **h**), 1.63 – 1.19 (m, 40H, **g** & **i**), 0.96 – 0.79 (m, 18H, **j** & **k**).

**^13^C NMR** (126 MHz, CDCl_3_) δ/ppm 164.57, 163.68, 142.08, 128.33, 125.83, 123.62, 123.36, 123.30, 123.16, 122.11, 121.48, 117.52, 55.79, 52.01, 32.62, 32.04, 31.88, 29.78, 29.25, 27.13, 26.89, 22.80, 22.74, 14.25, 14.22.

**HRMS** (ESI^+^) found 1053.6737, [M+H]^+^ requires 1053.6689.

### Tetrabutylammonium BINOL-phosphate 7a/b

To a round-bottom flask equipped with a stirrer bar under nitrogen atmosphere was added (+)- or (−)-BINOL-phosphoric acid (300 mg, 0.86 mmol). The solid was suspended in 1:1 MeCN:H_2_O (10 mL) and stirred vigorously. Ag_2_O is then added slowly and portionwise until all of the BINOL-phosphoric acid has dissolved and no more Ag_2_O is dissolving. The resulting suspension was then added dropwise *via* syringe filter (0.45 µm) to a stirring solution of tetrabutylammonium chloride (240 mg, 0.86 mmol, 1 eq.) in MeOH (15 mL). The solution became turbid upon addition and was left stirring for 1 hour. The resulting suspension was then diluted with CH_2_Cl_2_
(50 mL) and the organic layer washed with water (3x 50 mL), dried (MgSO_4_) and reduced *in vacuo* to yield the desired compounds as white solids.

**Tetrabutylammonium (+)-BINOL-phosphate 7a**

Yield: 338 mg, 0.57 mmol, 66%.

**^1^H NMR** (300 MHz, CDCl_3_) δ/ppm 7.87 (dd, *J* = 8.6, 7.0 Hz, 4H), 7.55 (dd, *J* = 8.8, 1.0 Hz, 2H), 7.35 (ddd, *J* = 9.3, 8.2, 2.0 Hz, 4H), 7.19 (ddd, *J* = 8.5, 6.9, 1.3 Hz, 2H), 3.16 – 2.94 (m, 8H), 1.44 (p, *J* = 7.2 Hz, 8H), 1.27 (h, *J* = 7.2 Hz, 8H), 0.89 (t, *J* = 7.2 Hz, 12H).

NMR data in accordance with the literature.^[4]^

**Tetrabutylammonium (−)-BINOL-phosphate 7b**

Yield: 497 mg, 0.84, 93%.

**^1^H NMR** (300 MHz, CDCl_3_) δ/ppm 7.87 (dd, *J* = 8.5, 7.0 Hz, 4H), 7.54 (dd, *J* = 8.8, 1.0 Hz, 2H), 7.42 – 7.29 (m, 4H), 7.19 (ddd, *J* = 8.5, 6.9, 1.3 Hz, 2H), 3.00 (tt, *J* = 9.5, 4.9 Hz, 8H), 1.40 (dq, *J* = 9.5, 7.0 Hz, 8H), 1.33 – 1.13 (m, 8H), 0.87 (t, *J* = 7.2 Hz, 12H).

NMR data in accordance with the literature.^[4]^

# Photophysical Properties

### General

UV-Visible absorbance spectra were recorded on a Shimadzu UV-3600i Plus Spectrometer with a wavelength accuracy of ± 0.2 nm in the visible and UV region, a baseline flatness of ± 0.002 nm (200 – 3000 nm) and a noise level of <0.00008 Abs (900 nm) or a Cary50 UV-vis spectrometer.

Fluorescence spectra were recorded on a Cary Eclipse fluorescence spectrometer and smoothed using a Savitzky-Golay filter.

Circular Dichroism measurements were taken on a JASCO J-1500 CD Spectrophotometer with a wavelength accuracy ± 0.2 nm (250 to 500 nm), ± 0.5 nm (500 to 800 nm), a photometric accuracy of ± 0.01 Abs and a CD root mean square noise < 0.007 mdeg (500 nm). Recorded traces were then baseline corrected and smoothed using the provided software.

Quantum yields (Φ_obs_) were recorded on an Edinburgh Instruments FLS1000 Photoluminescence Spectrometer equipped with an integrating sphere and obtained using direct methods. Samples were prepared at ~0.1 OD at the excitation wavelength. Spectra in MeCN and PhMe were recorded with an excitation bandwidth of 6 nm, an emission bandwidth of 0.55 nm with 0.5 nm steps and a dwell time of 0.5 s. Quantum yields were calculated using the Fluoracle software, and errors of the calculated yields are ±2%.


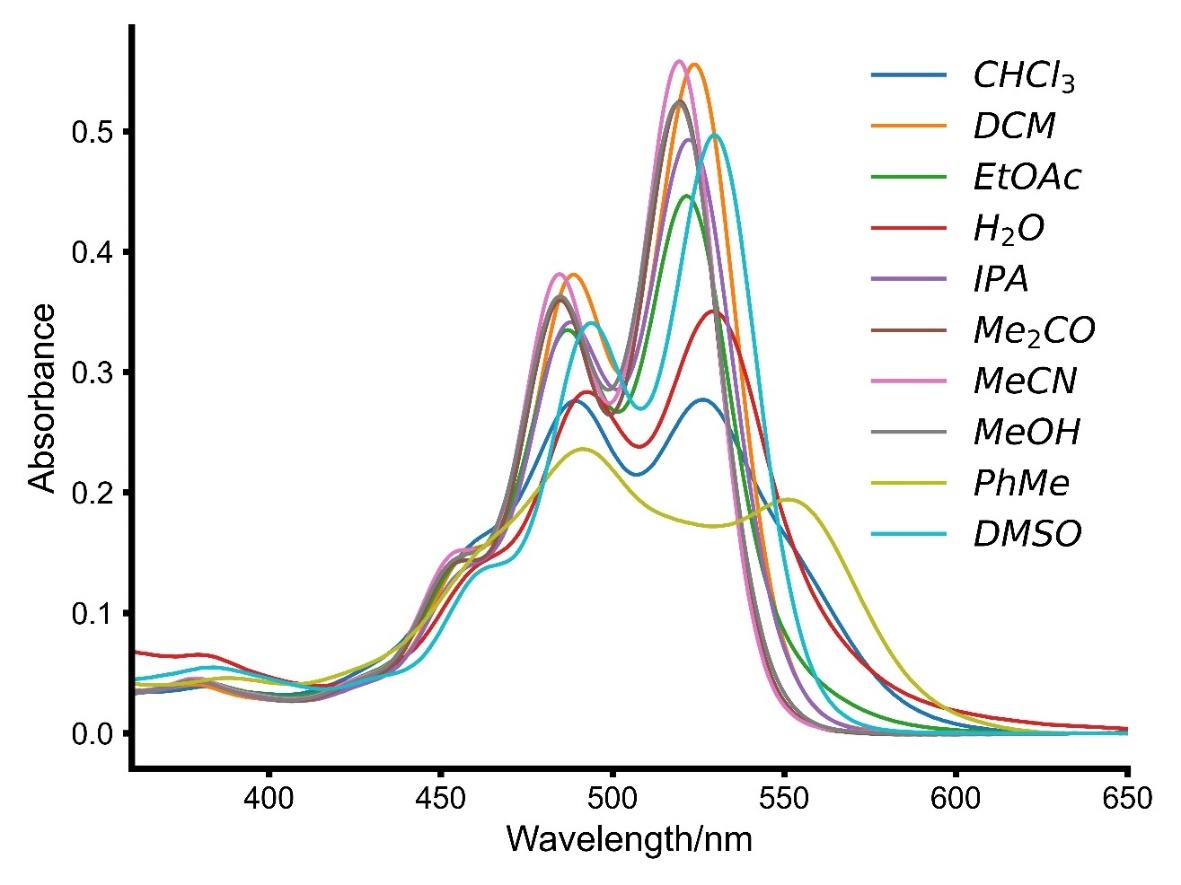


**Supplementary Figure 2‑1:** Absorbance Spectra of 1,7-ditriazolium PDI **4a** in various solvents (all with 0.2% DMSO) at 10 µM concentration.


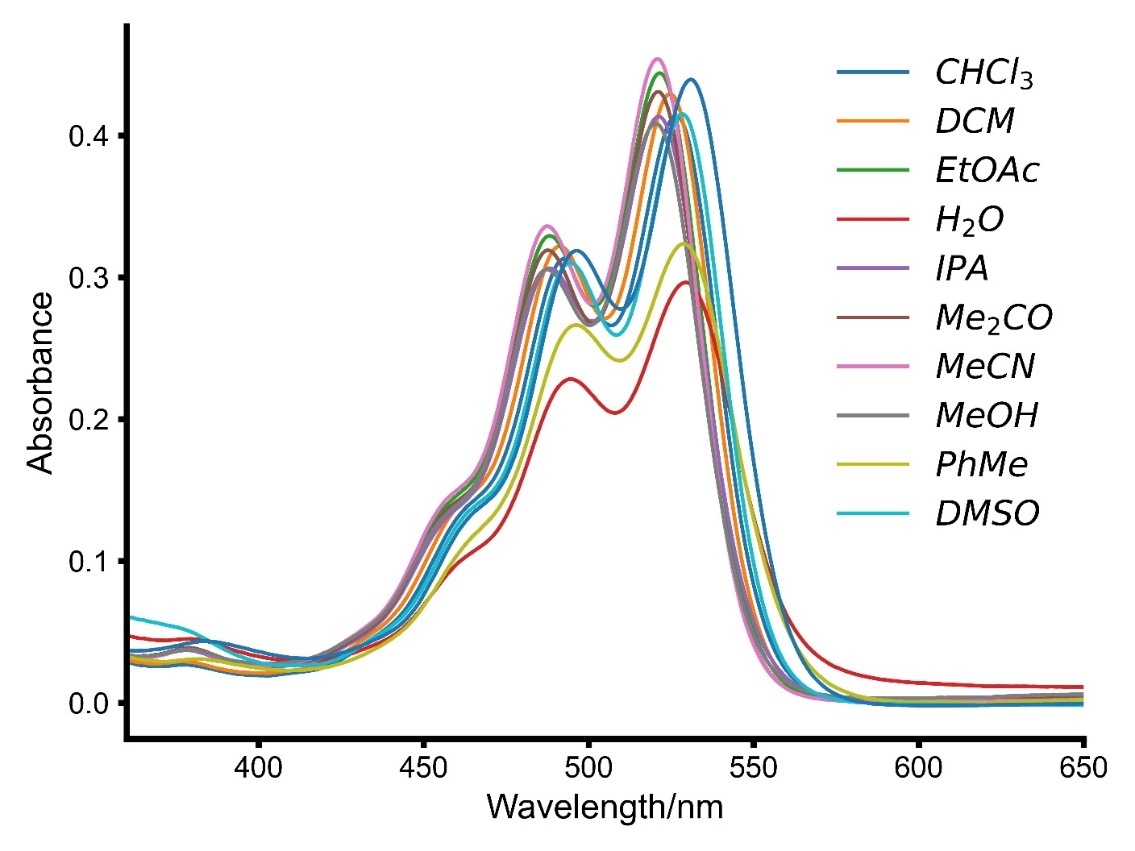


**Supplementary Figure 2‑2:** Absorbance Spectra of 1,6-ditriazolium PDI **4b** in various solvents (all with 0.2% DMSO) at 10 µM concentration. Note: 1,6-Isomer **4b** is essentially insoluble in neat PhMe.


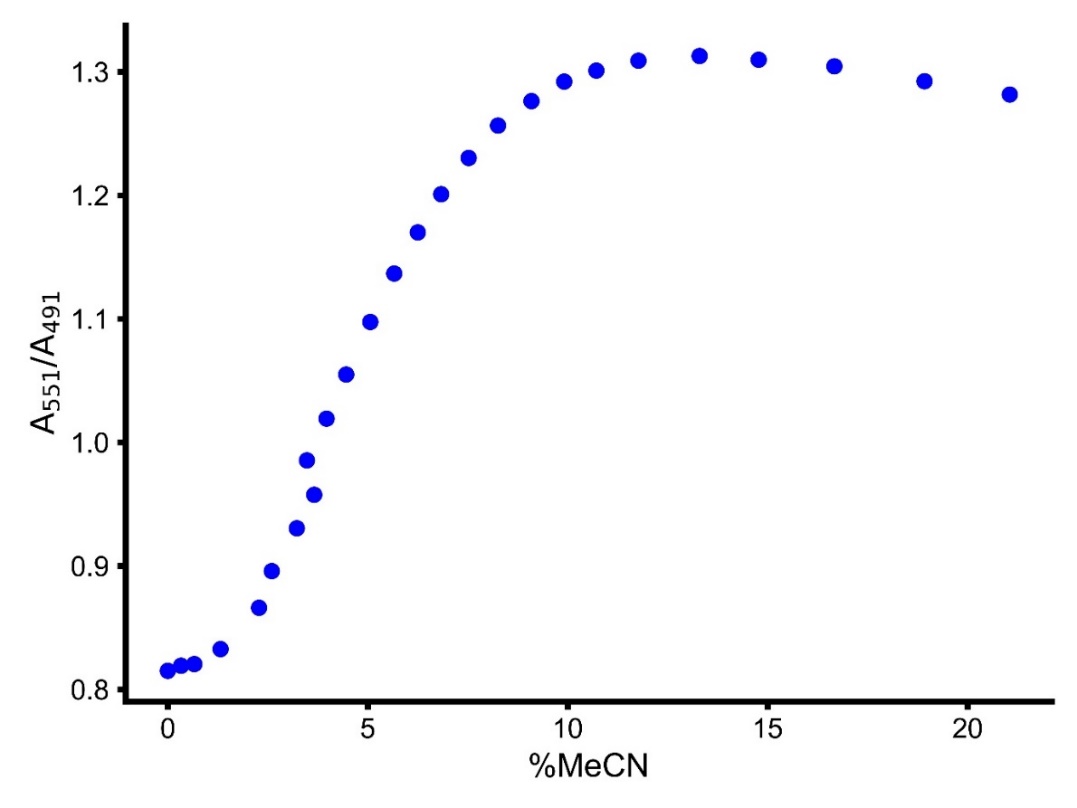


**Supplementary Figure 2‑3**: Ratio of A_0-0_/A_0-1_ transitions of **Figure 3**f. Complete disaggregation is observed at ~12% MeCN.


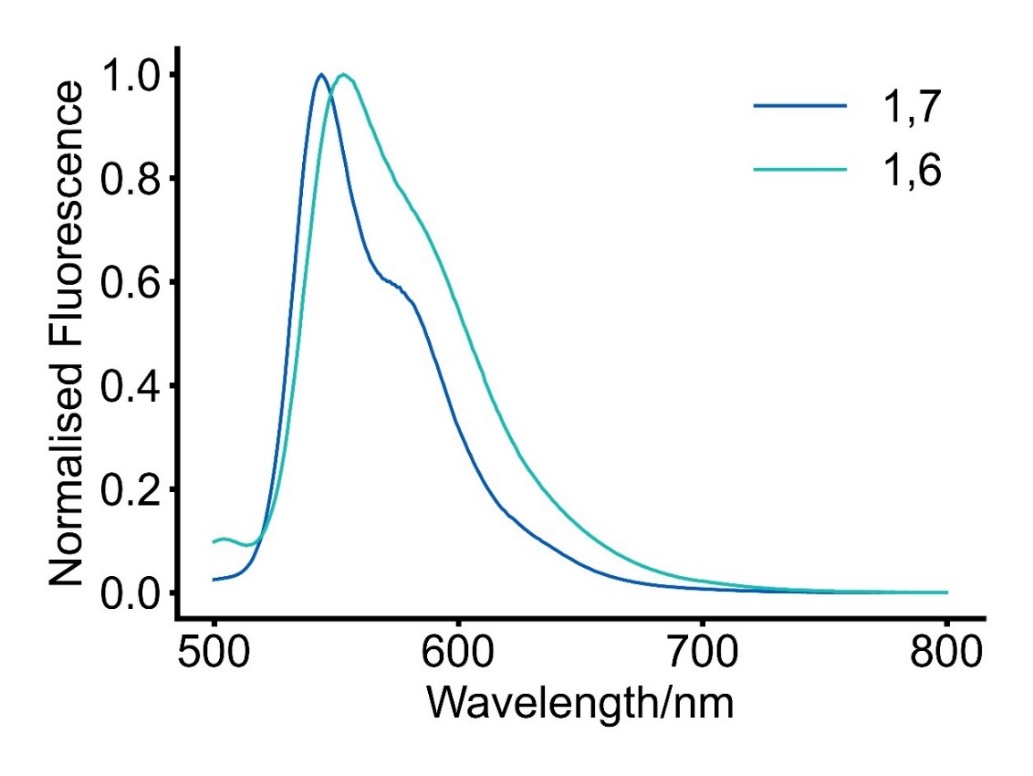


**Supplementary Figure 2‑4:** Difference in fluorescence spectra of the 1,7- and 1,6- ditriazolium PDIs **4a/b** in MeCN. Compounds were prepared at 5 µM concentration and excited at 470 nm at medium gain (600 V) and normalised.


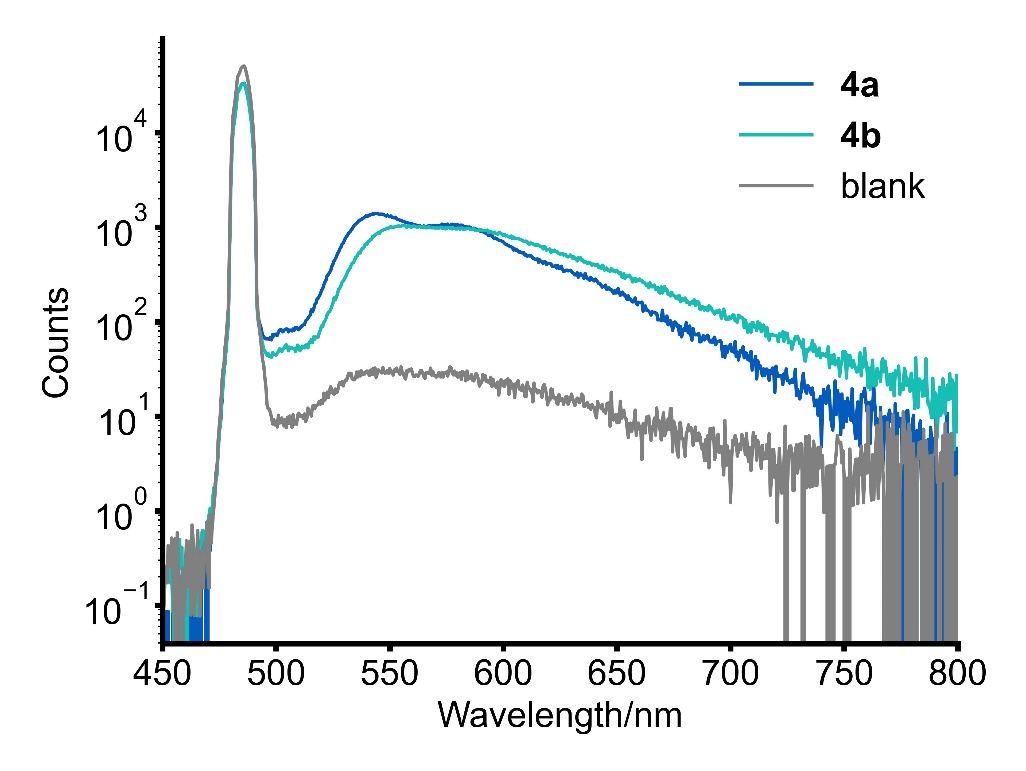


**Supplementary Figure 2‑5:** Quantum yield measurement of triazolium-PDIs **4a/4b** in MeCN, excited at 485 nm and recorded up to 800 nm. The quantum yields were determined as 95% and 92%, respectively.


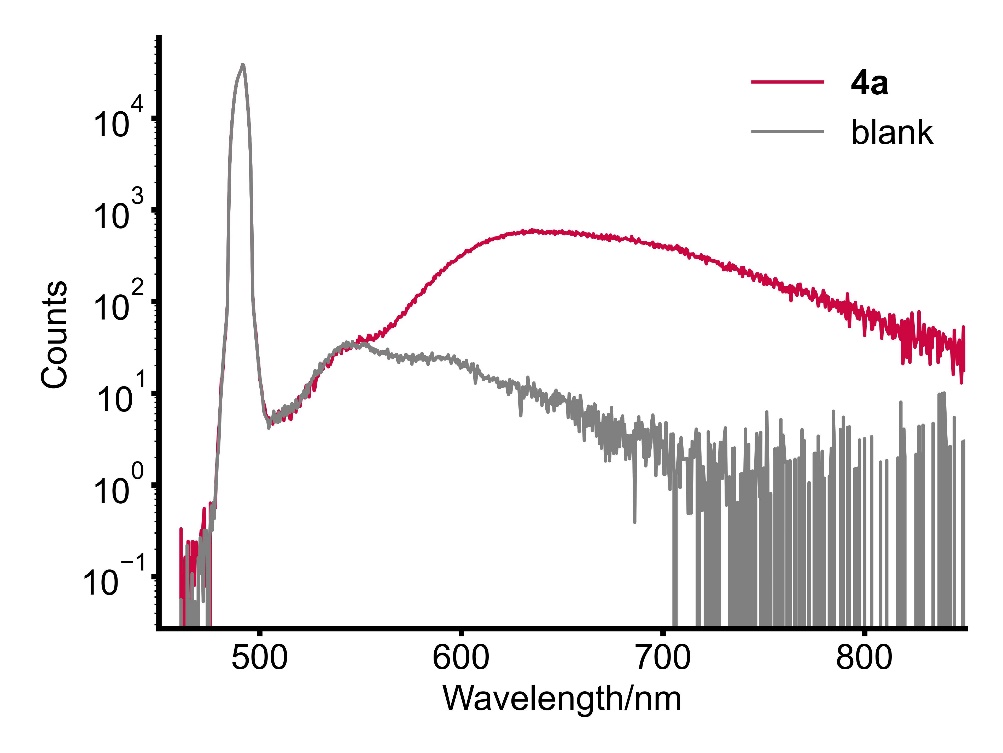


**Supplementary Figure 2‑6:** Quantum yield measurement of triazolium-PDI **4a** in PhMe, excited at
490 nm and recorded up to 850 nm. The quantum yield was determined as 72%.

**Supplementary Table 2‑1**: Observed quantum yields obtained for the 1,7- and 1,6-ditriazolium PDIs.

| **Index** | **Species** | **Solvent** | **λ_Ex_/nm** | **Quantum Yield Φ_obs_ (%)** |
| --- | --- | --- | --- | --- |
| 1 | **4a** | MeCN | 485 | 95 |
| 2 | **4b** | MeCN | 485 | 92 |
| 3 | **4a** | PhMe | 490 | 72 |

# Aggregation Behaviour


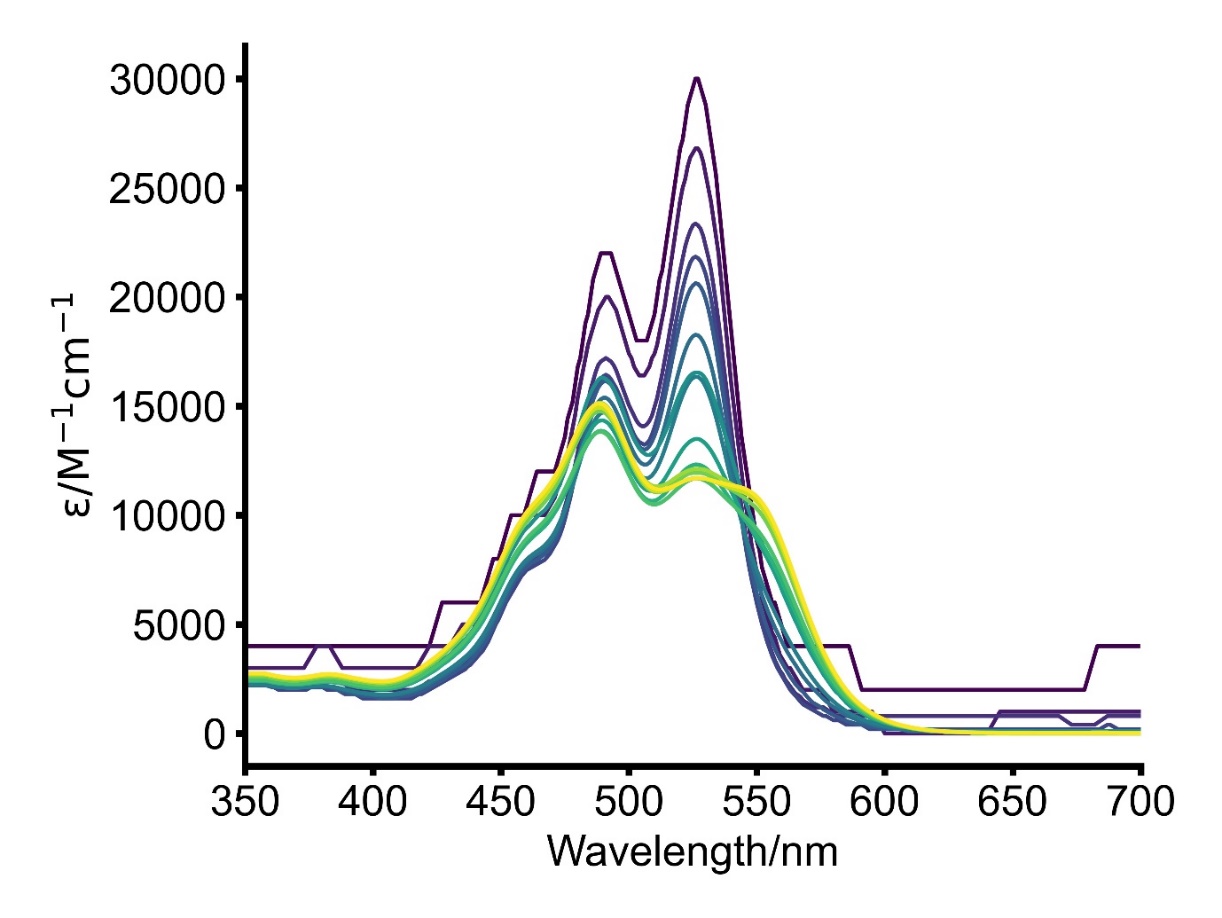


**Supplementary Figure 3‑1**: Aggregation of 1,7-ditriazolium PDI **4a** in CHCl_3_ over the concentration range of 50 nM (purple) to 805 µM (yellow).

**Supplementary Table 3‑1:** Parameters obtained from fitting the data from **Supplementary Figure 3-1** to a dimer model.

|  | **Dimer Model** |
| --- | --- |
| *K_dim_* | 125055.88625 ± 11059.73506 |
| A_m_ | 1.38529 ± 0.00748 |
| A_d_ | 0.72403 ± 0.00678 |
| Adj. R-Square | 0.99749 |


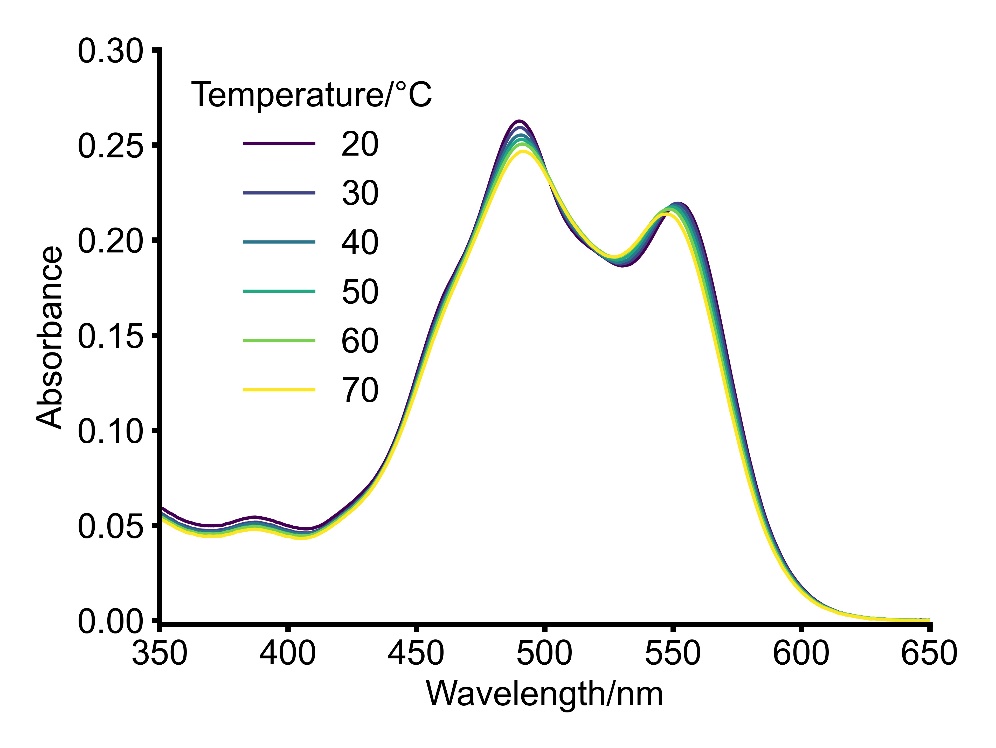


**Supplementary Figure 3‑2:** Absorbance spectrum of 1,7-ditriazolium **4a** in PhMe (10 µM) at various temperatures. Only minor variations are observed, indicating little disaggregation.


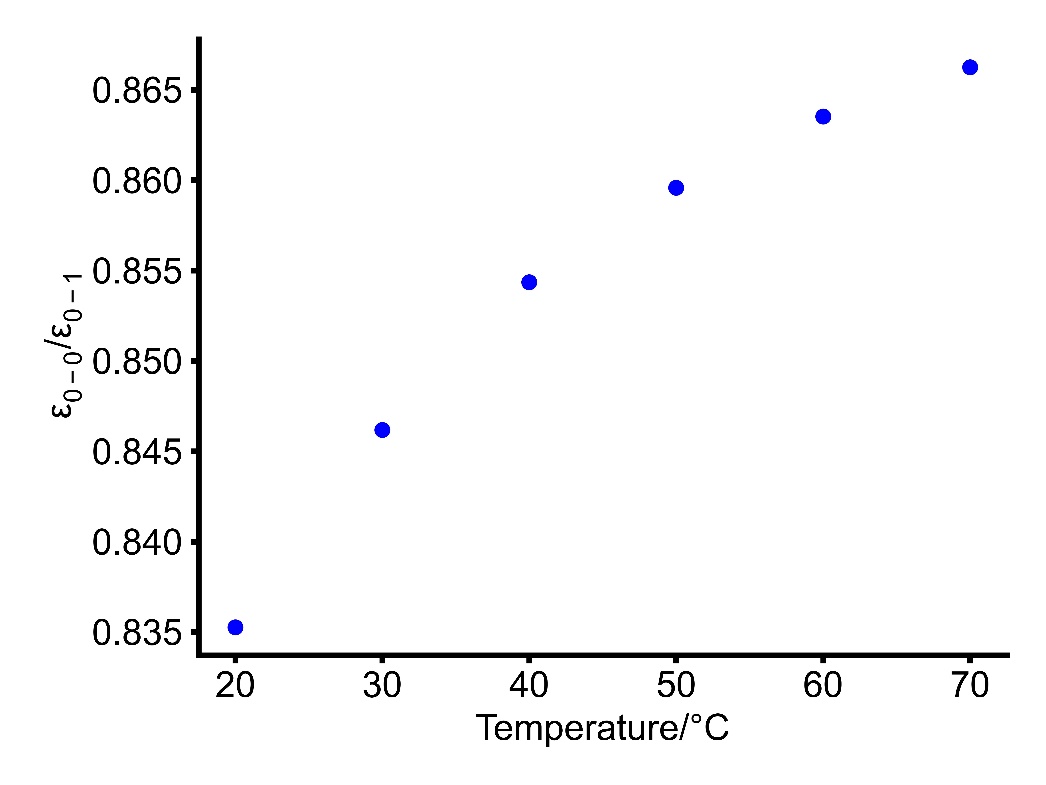


**Supplementary Figure 3‑3:** Plot of the ratio of the 0-0 and 0-1 transitions in **Supplementary Figure 3-2** in PhMe. Only minor disaggregation is observed over the range of temperatures measured.


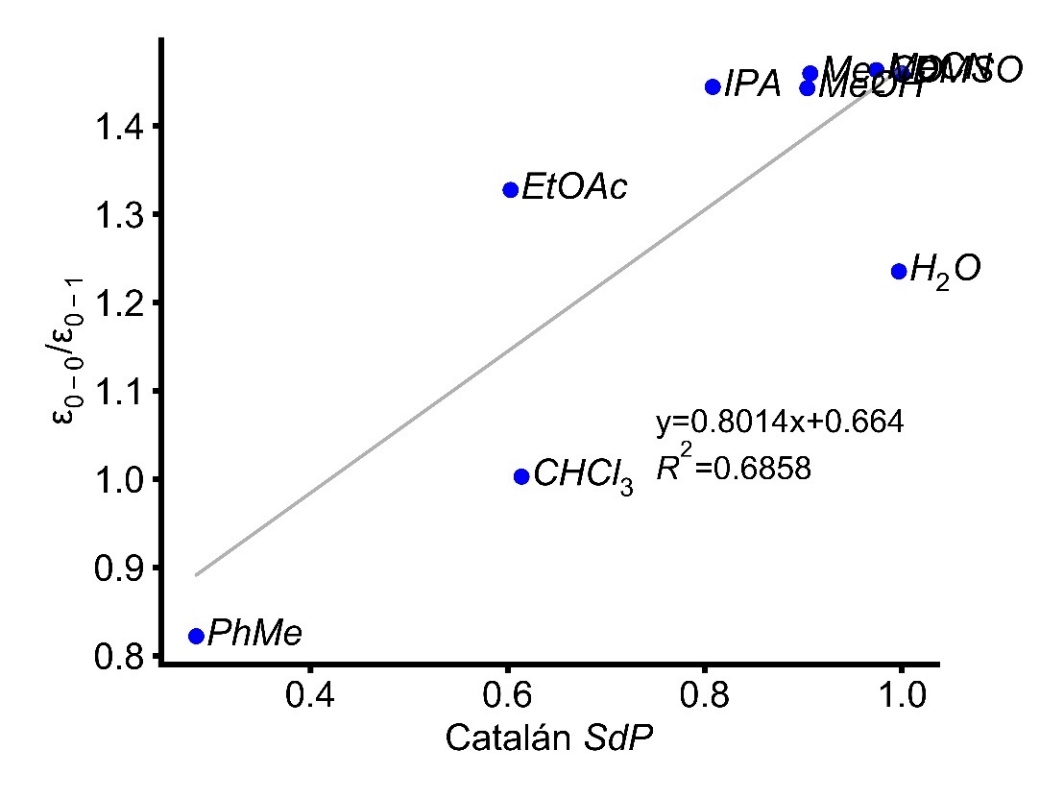


**Supplementary Figure 3‑4:** Ratio of extinction coefficients of the 0-0 and 0-1 vibronic transitions of **4a** in various solvents against the Catalán solvent dipolarity (SdP) scale.


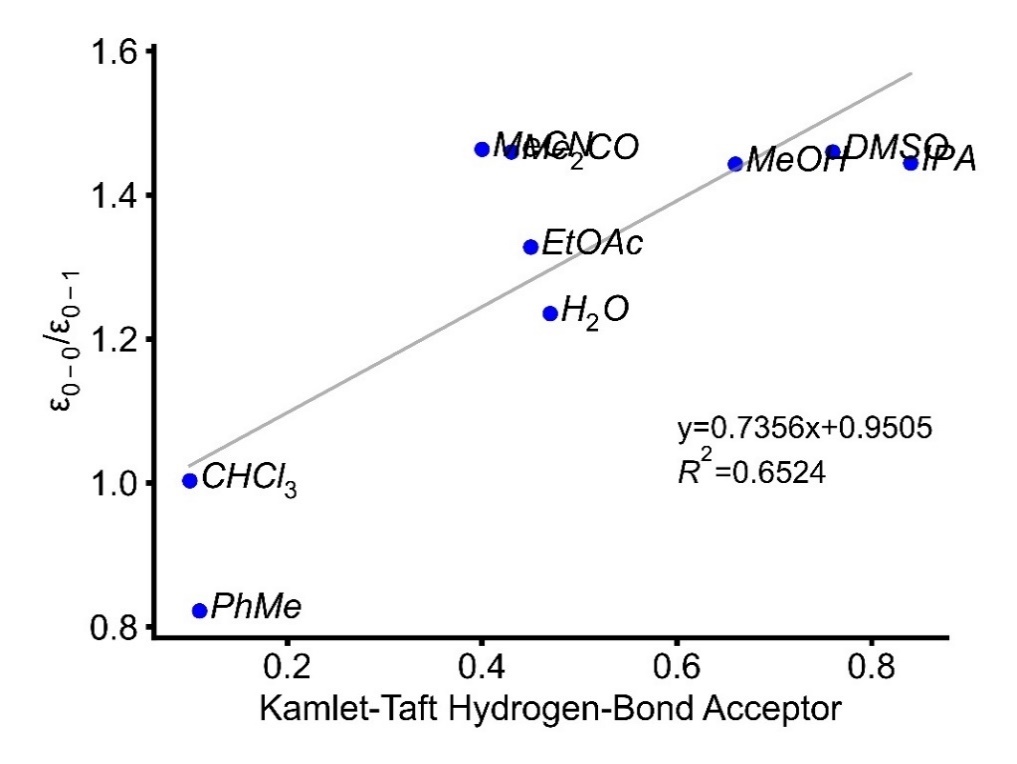


**Supplementary Figure 3‑5:** Ratio of extinction coefficients of the 0-0 and 0-1 vibronic transitions of **4a** in various solvents against the Kamlet-Taft scale of hydrogen-bond accepting ability.


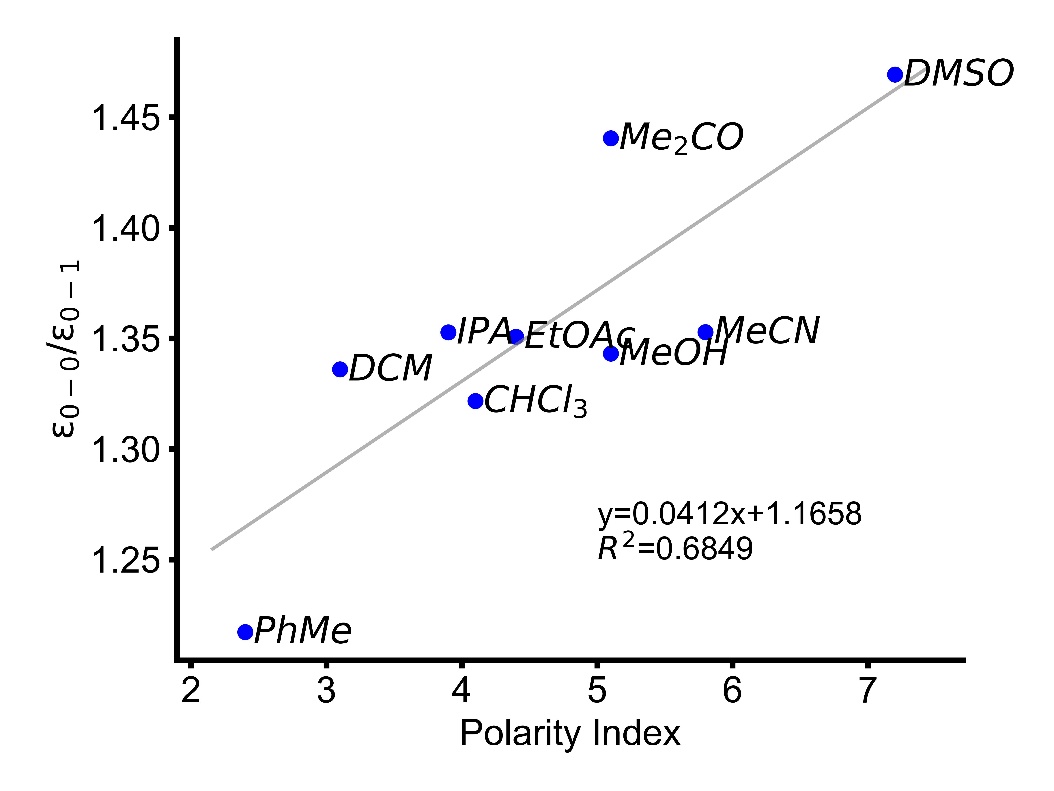


**Supplementary Figure 3‑6**: Ratio of extinction coefficients of the 0-0 and 0-1 vibronic transitions of **4b** in various solvents against polarity index.


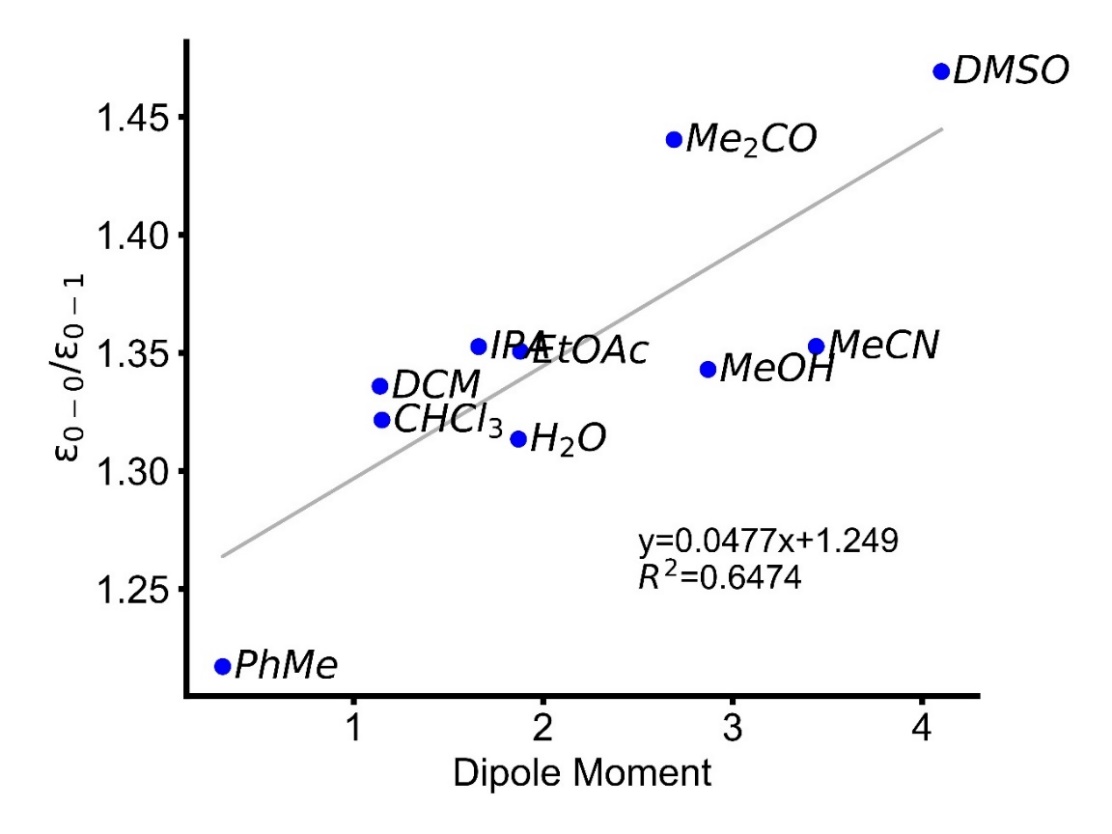


**Supplementary Figure 3‑7:** Ratio of extinction coefficients of the 0-0 and 0-1 vibronic transitions of **4b** in various solvents against dipole moment.


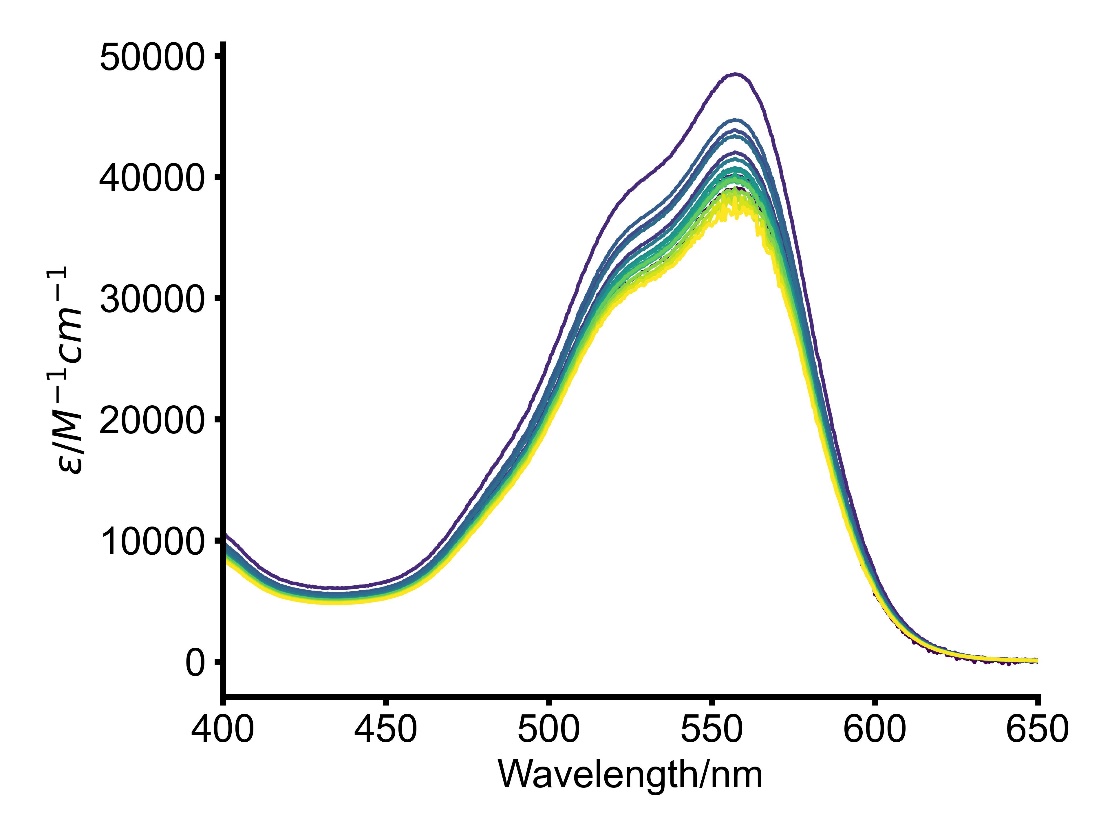


**Supplementary Figure 3‑8**: Change of absorbance profile of 1,7-ditriazole PDI **3a** in PhMe over a concentration range of 19 to 620 mM (purple to yellow).


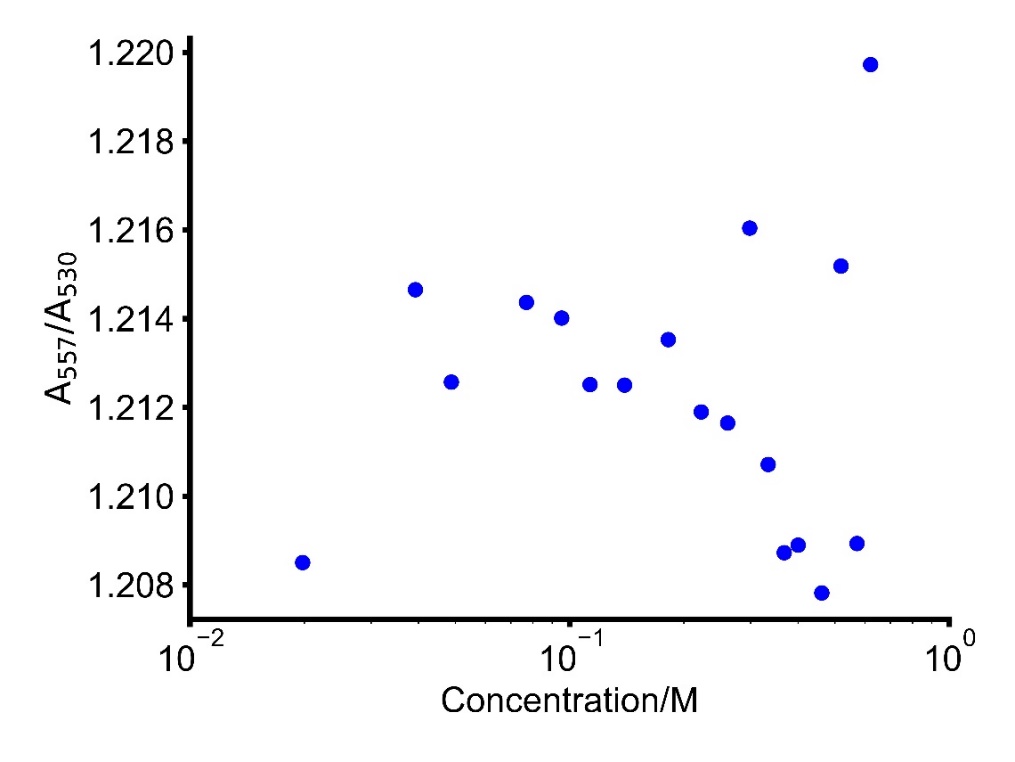


**Supplementary Figure 3‑9:** Ratio of absorbances at 557 nm and 530 nm, corresponding to the 0-0 and 0-1 transitions of PDI **3a** observed in **Supplementary Figure 3-8**. No clear trend is observed, and variations are all on a very small magnitude, meaning that no aggregation occurred over the measured concentration range.


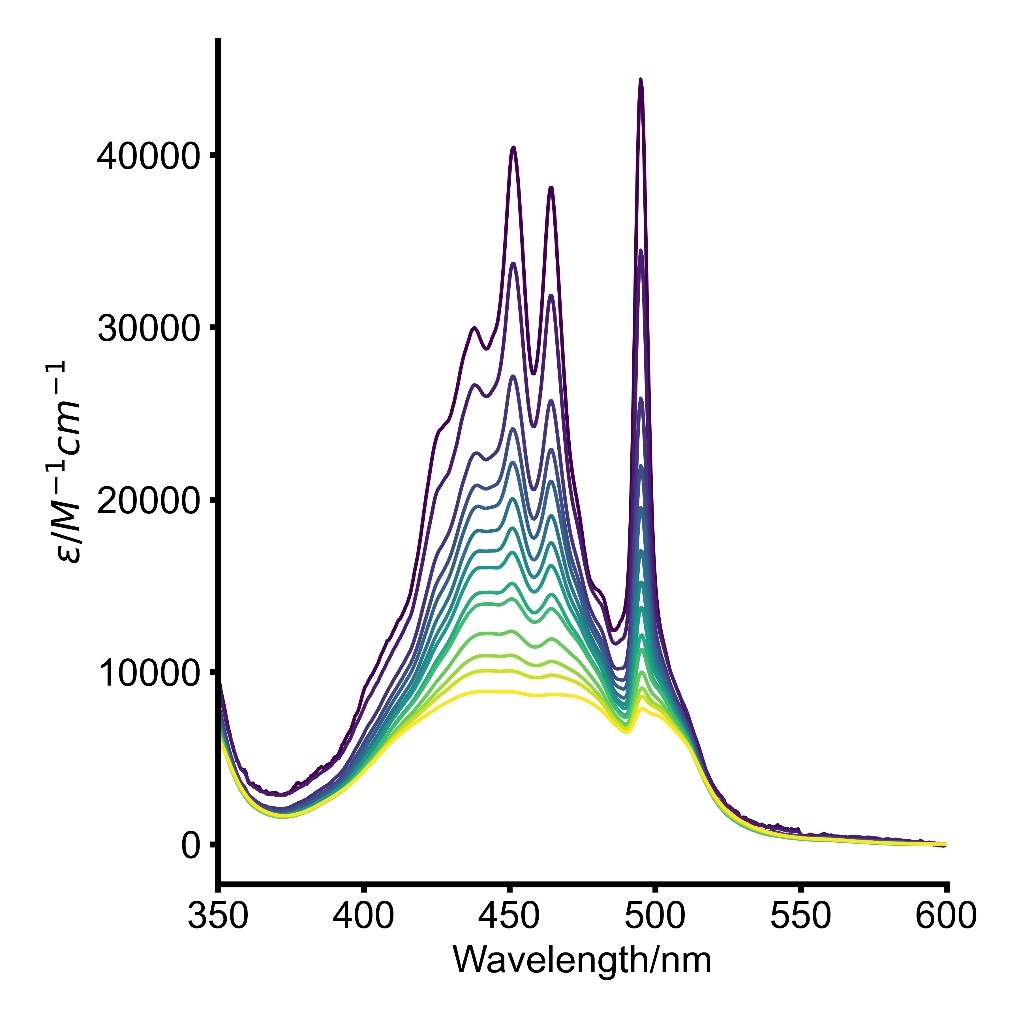


**Supplementary Figure 3‑10:** Change in absorbance profile of C11-CDI **6** in cyclohexane with varying concentration (1.6x10^-7^ M (purple) to 4.3x10^-5^ M (yellow)).


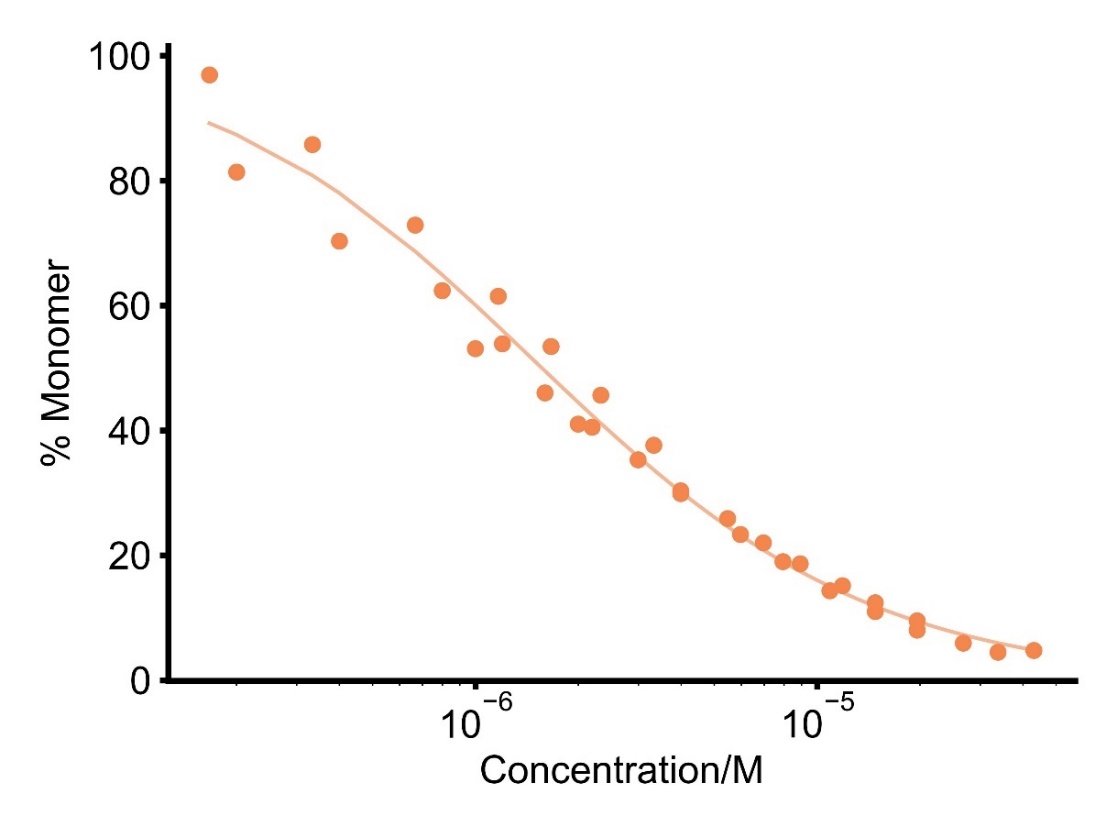


**Supplementary Figure 3‑11:** %Monomer against concentration (obtained from the A_0-0_/A_0-1_ ratio of peaks in **Supplementary Figure 3-10**) fitted to an isodesmic model (solid line).

**Supplementary Table 3‑2:** Parameters obtained from fitting the data from **Supplementary Figure 3-10** to an isodesmic model.

|  | **Isodesmic Model** |
| --- | --- |
| *K_a_* | 374427.1709 ± 46921.08764 |
| A_a_ | 0.80602 ± 0.01662 |
| A_m_ | 1.73066 ± 0.03069 |
| Adj. R^2^ | 0.98042 |

# Anion Binding


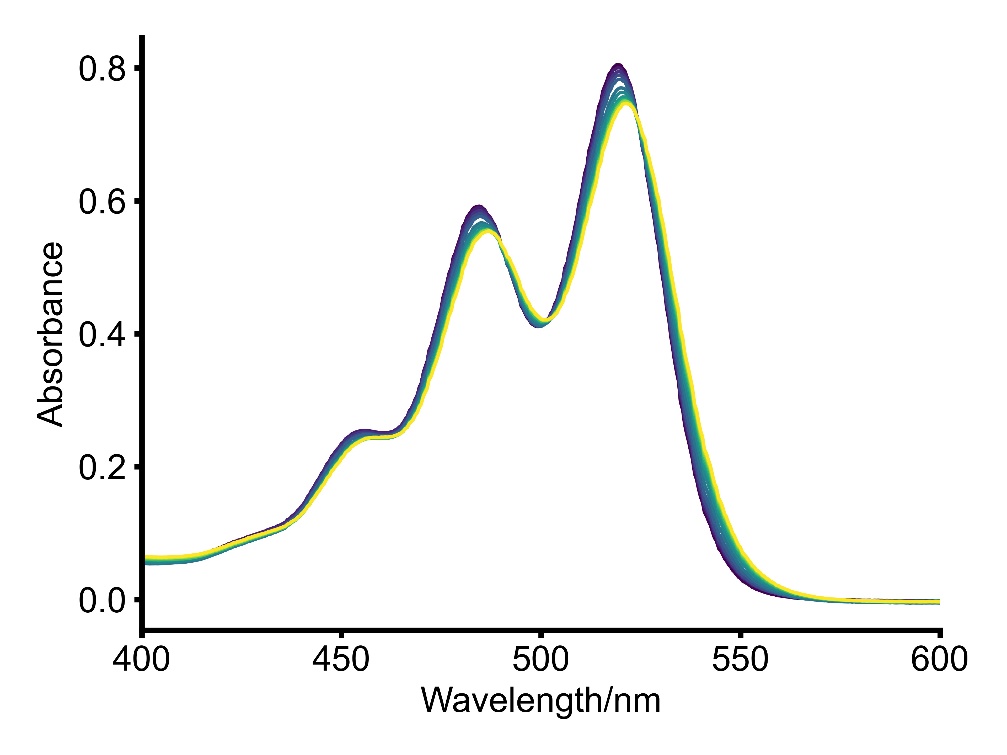


**Supplementary Figure 4‑1**: Change in absorbance spectrum of 1,7-ditriazolium PDI **4a** at 10 µM in MeCN upon addition of TBACl (0-31.9 mM). Fitting the data where the largest spectral changes are observed (460-555 nm) to a 1:2 binding model yielded a K_11_ of 1148 M⁻¹ (± 0.8%) and a K_12_ of 59 M⁻¹ (± 0.4%). <http://app.supramolecular.org/bindfit/view/68ba6ead-d260-45be-95c3-a47a62e4f9ca>.


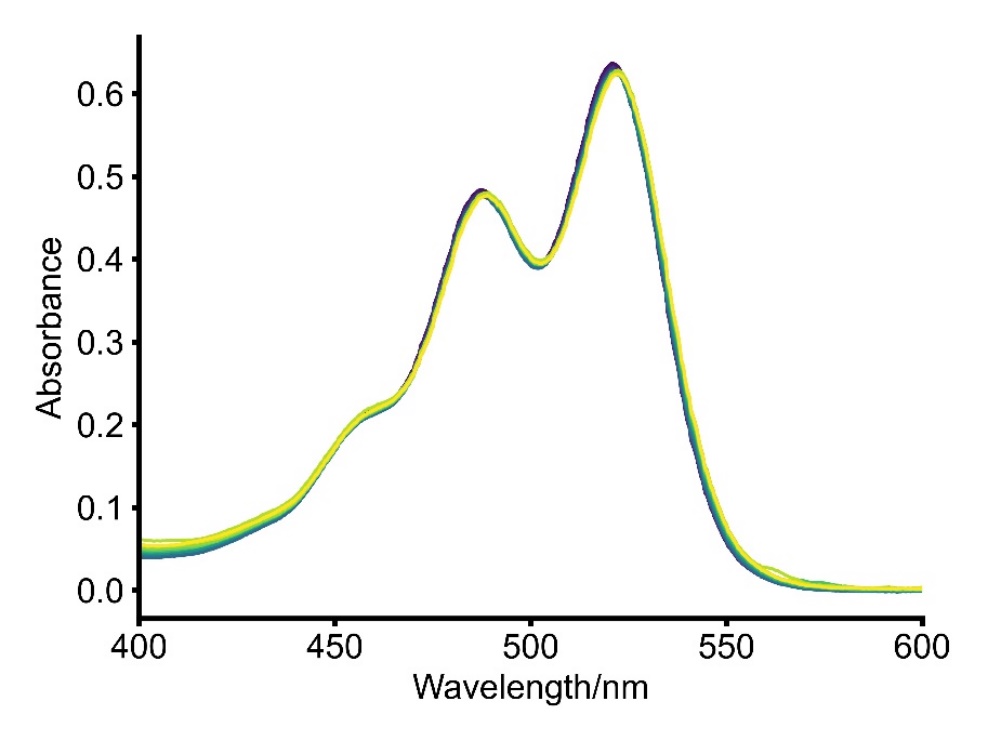


**Supplementary Figure 4‑2:** Change in absorbance spectrum of 1,6-ditriazolium PDI **4b** at 10 µM in MeCN upon addition of TBACl (0-37.4 mM). Fitting the data where the largest spectral changes are observed (470-550 nm) to a 1:2 binding model yielded a K_11_ of 2963 M⁻¹ (± 4%) and a K_12_ of 89 M⁻¹
(± 0.9%). <http://app.supramolecular.org/bindfit/view/64ceea67-db68-4fd6-9922-7de15be1d6e8>.


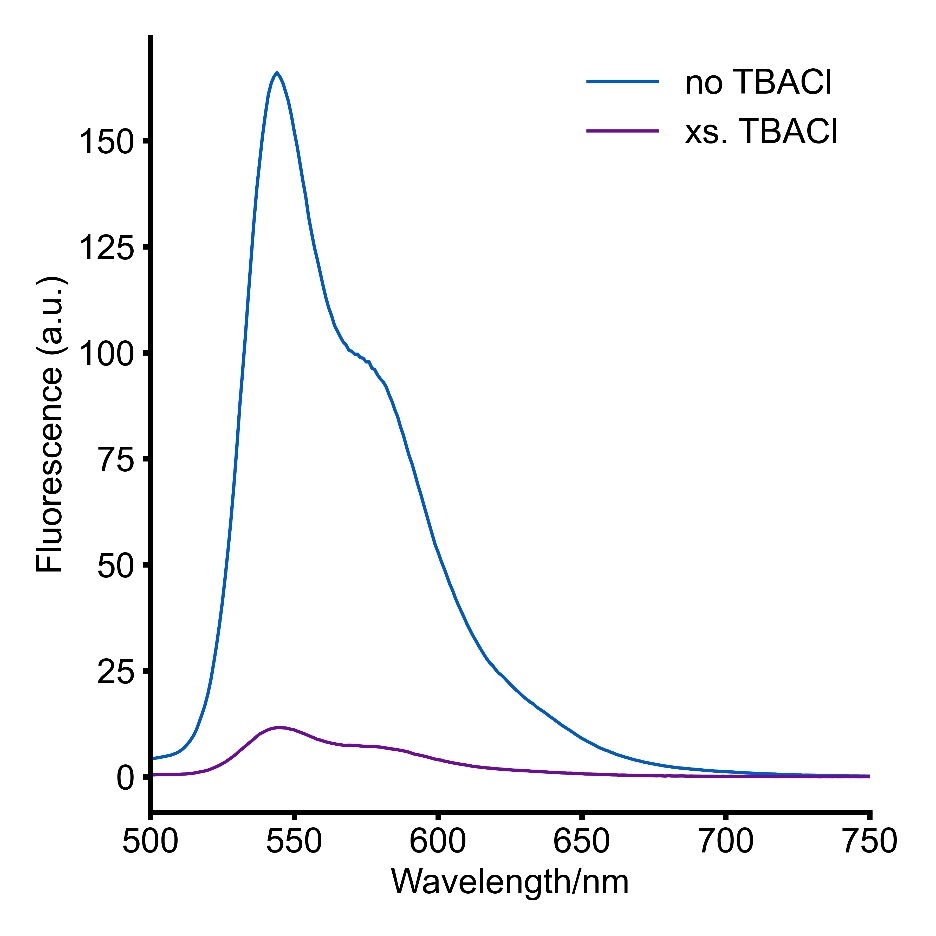


**Supplementary Figure 4‑3:** Change of Fluorescence intensity of a sample of 1,7-ditriazolium PDI **4a** at 5 µM in MeCN excited at 470 nm and recorded at low gain upon addition of a large excess of tetrabutylammonium chloride. Anion binding shows clear fluorescence quenching.

### Binding of TBACl in PhMe


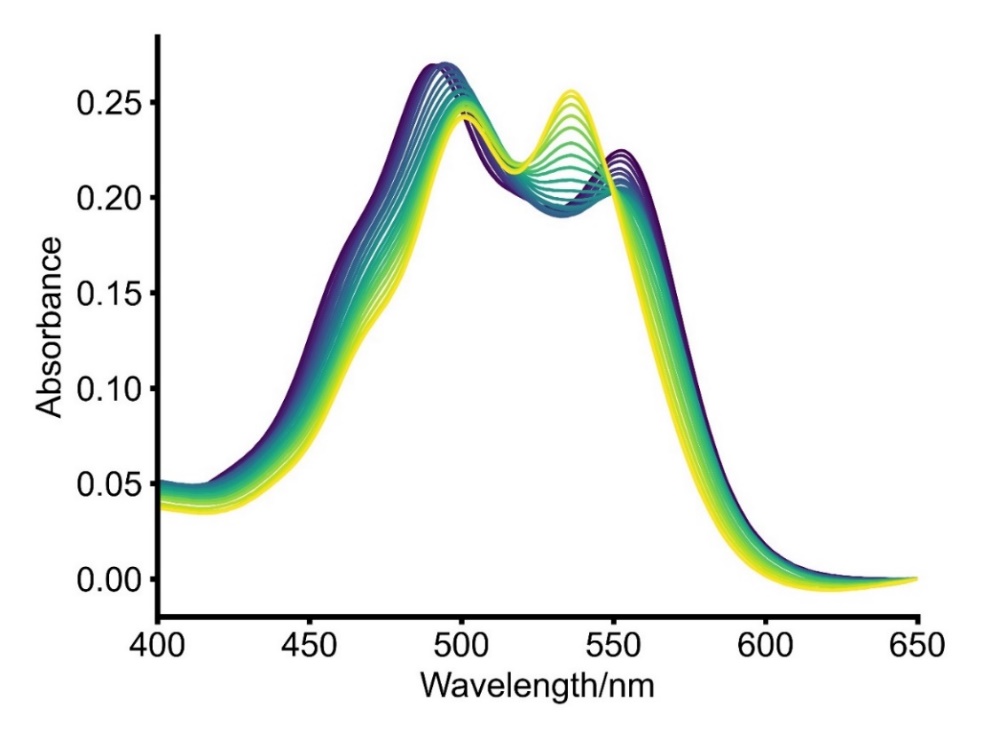


**Supplementary Figure 4‑4:** Change in absorbance spectrum of 1,7-ditriazolium PDI **4a** at 10 µM concentration upon addition of TBACl (0-0.65 mM). Fitting the data where the largest spectral changes are observed (450-545 nm) to a 1:2 binding model yielded a K_11_ of 77465 M⁻¹ (± 2.1%) and a K_12_ of 97 M⁻¹ (± 0.5%). <http://app.supramolecular.org/bindfit/view/5819a13c-0b1e-4d8e-ba99-7143aebcd909>.


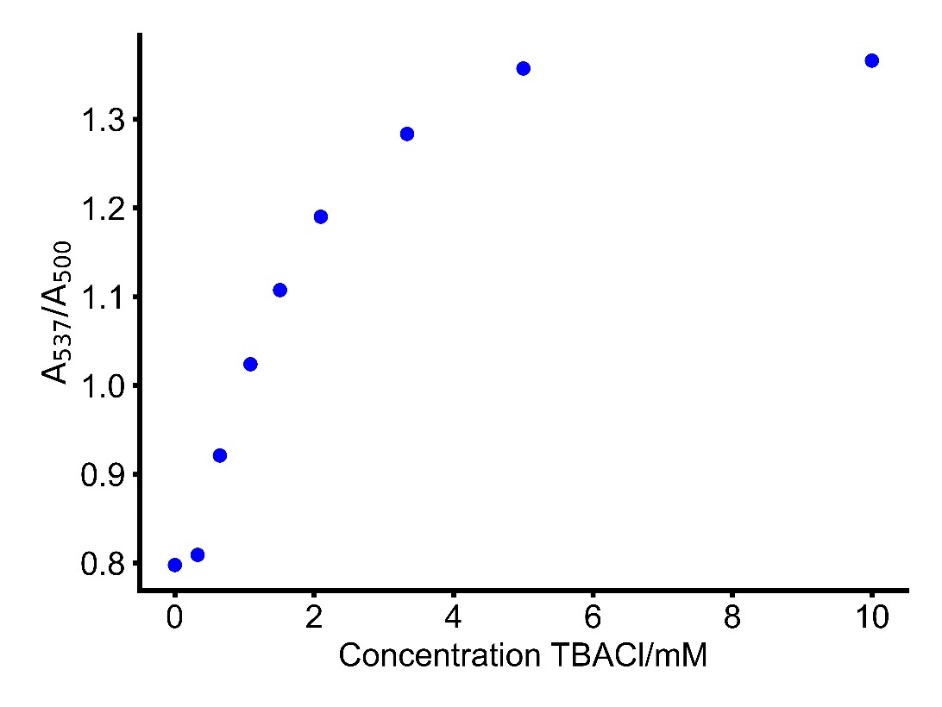


**Supplementary** **Figure 4‑5:** Ratio of A_0-0_/A_0-1_ of the disaggregation of 1,7-ditriazolium PDI **4a** (10 µM) in PhMe from **Figure 5**c at different concentrations of TBACl (0-10 mM).

### (+)-CSA Binding in PhMe


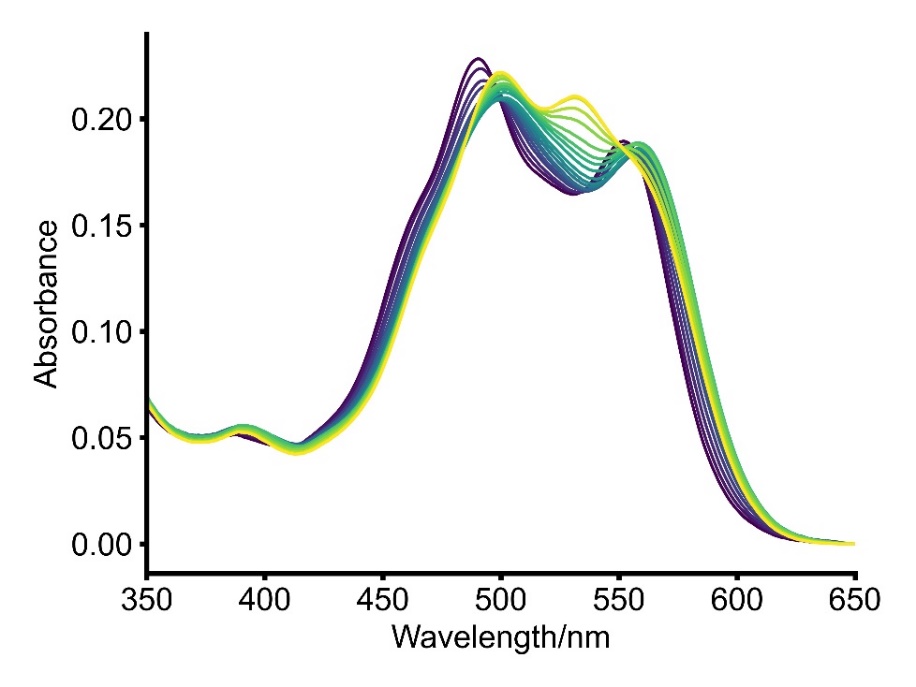


**Supplementary Figure 4‑6:** Change in absorbance spectrum of 1,7-bistriazolium PDI **4a** at 10 µM concentration upon addition of tetrabutylammonium (+)-camphorsulfonate (0-0.69 mM). Fitting the data where the largest spectral changes are observed (435-485 nm, 512-545 nm, 652-600 nm) to a 1:2 binding model yielded a K_11_ of 290790 M⁻¹ (±1%) and a K_12_ of 7966 M⁻¹ (±1%). <http://app.supramolecular.org/bindfit/view/3273eaa6-fb97-4564-9ade-555a42aa6170>


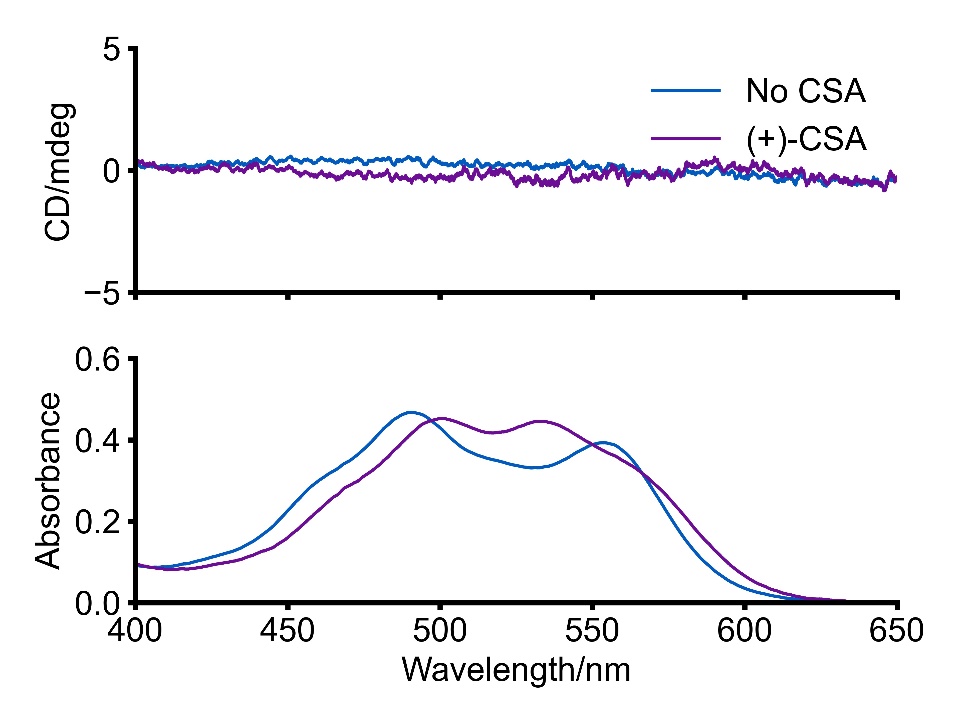


**Supplementary Figure 4‑7**: Change in CD and UV-Vis Spectra of a 25 µM solution of 1,7-ditriazolium PDI **4a** in PhMe (25 µM) upon addition of a large excess of tetrabutylammonium (+)-camphorsulfonate (~100 eq.). No clear CD signal was induced.

### Change in CD Spectrum upon binding of Δ-Trisphat in PhMe


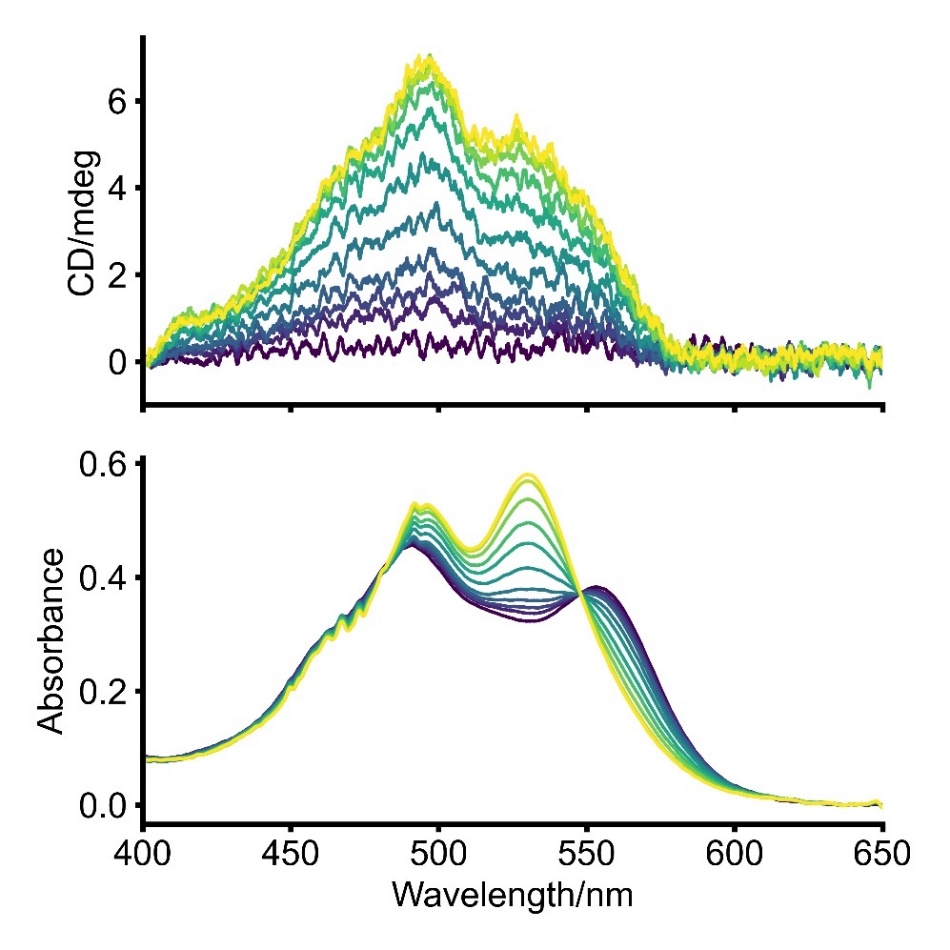


**Supplementary Figure 4‑8:** Change in CD and UV-Vis Spectra of a 25 µM solution of 1,7-ditriazolium PDI **4a** in PhMe upon addition of a solution of tetrabutylammonium Δ-Trisphat from 0 to 1.3 mM. Binding shows weak induction of CD and disaggregation of the dimeric species by UV-visible absorbance. Fitting the UV-vis data where a large spectral change is observed (550-600 nm) to a 1:2 binding model yields a K_11_ of 48464 M⁻¹ (± 1.4%) and a K_12_ of 258 M⁻¹ (± 0.1%). <http://app.supramolecular.org/bindfit/view/4a90a3db-5506-40f6-9321-ab05dc180266>

### Binding of tetrabutylammonium BINOL-phosphate in PhMe


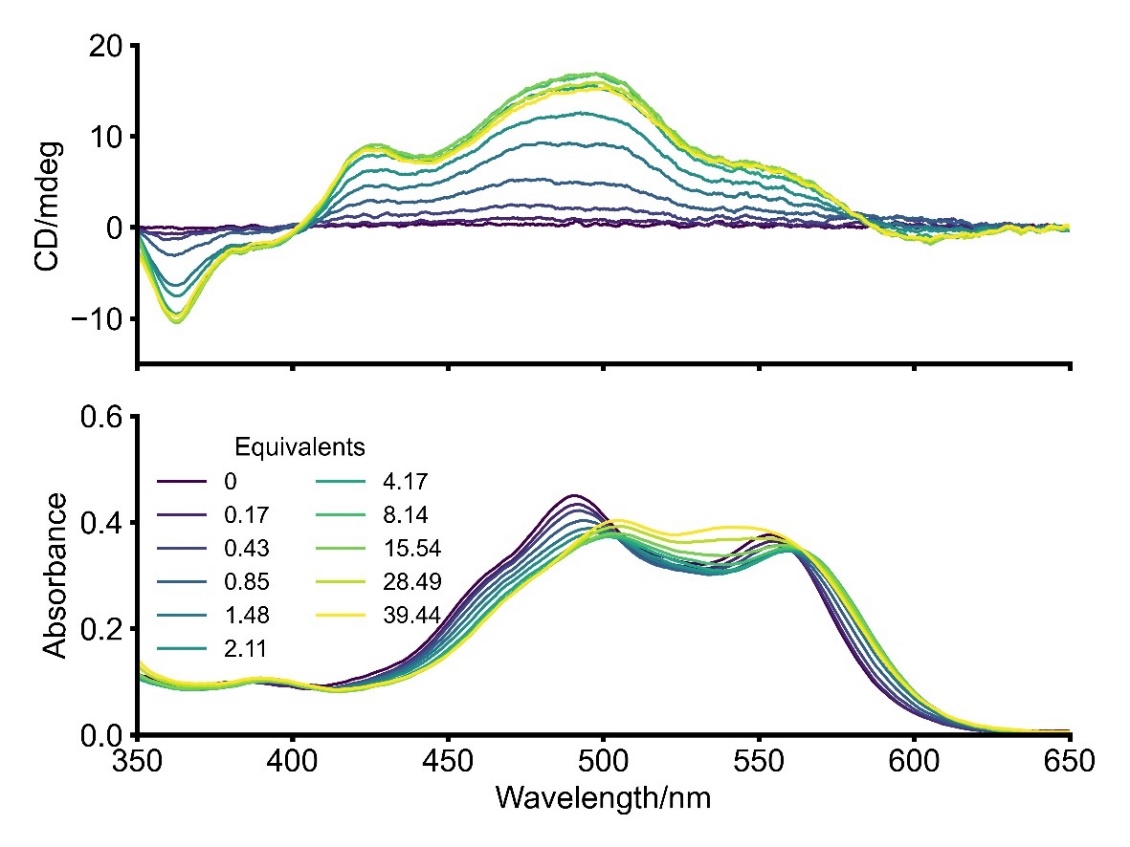


**Supplementary Figure 4‑9:** Change in CD and UV-vis spectra of 1,7-ditriazolium-PDI **4a** in PhMe at 25 µM concentration upon addition of tetrabutylammonium (+)-BINOL-phosphate **7a** (0-39 equivalents). Fitting the data where a significant spectral change is observed (550-600 nm) to a 1:2 binding model yielded a K_12_ of 45559 M⁻¹ (±1%) and a K_12_ of 2828 M⁻¹ (±1%). <http://app.supramolecular.org/bindfit/view/1bf4f746-5c3f-40ea-8f8c-a748ab966d43>


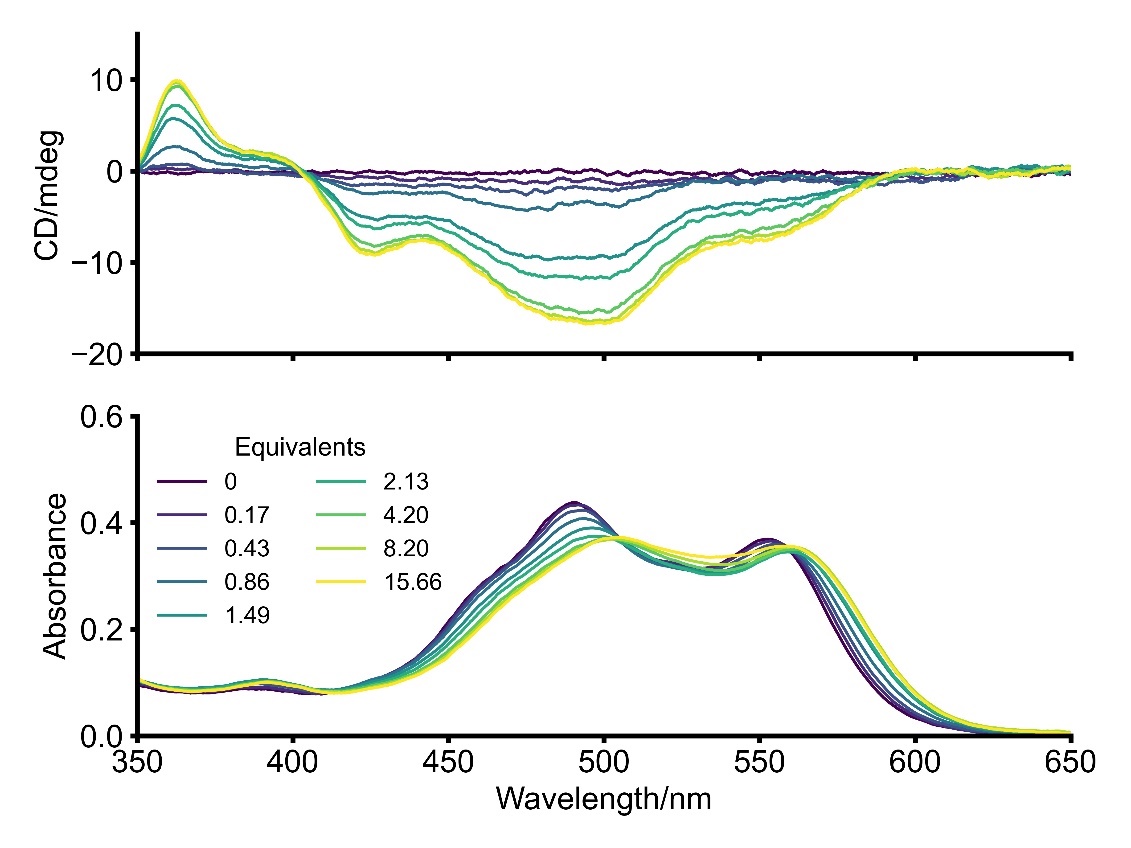


**Supplementary Figure 4‑10:** Change in CD and Absorbance Spectrum of 1,7-bistriazolium-PDI **4a** in PhMe at 25 µM concentration upon addition of tetrabutylammonium (−)-BINOL-phosphate **7b**
(0-15 equivalents).

Colour change of a solution of **4a** in PhMe upon addition of **7b**


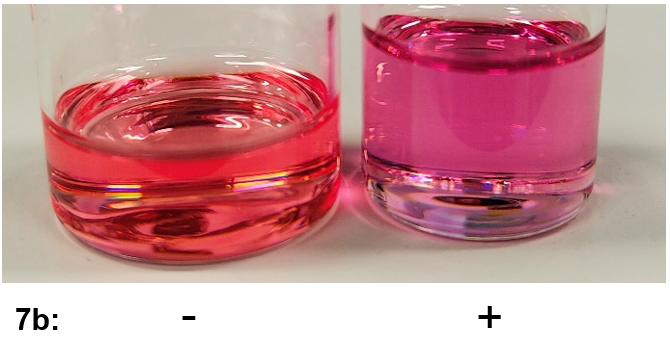


**Supplementary Figure 4‑11:** Change in colour of a solution of **4a** in PhMe (25 µM) upon addition of **7b** (1.25 mM).

# Computational Chemistry

### General

Alkyl chains (imide and C8-chains on triazole) have been simplified as Me groups for all calculations. Density functional theory (DFT) calculations were performed using ORCA 5.0.3. All functionals and basis sets were chosen following the best-practice DFT protocols published by Grimme.^[5]^

### Geometry Optimisation by DFT of ditriazole-PDI 3a

1,7-ditriazole **3a** was optimised by means of density functional theory using the B97-3c basis set by Grimme and co-workers,^[6]^ and the def2-TZVP functional.^[7]^


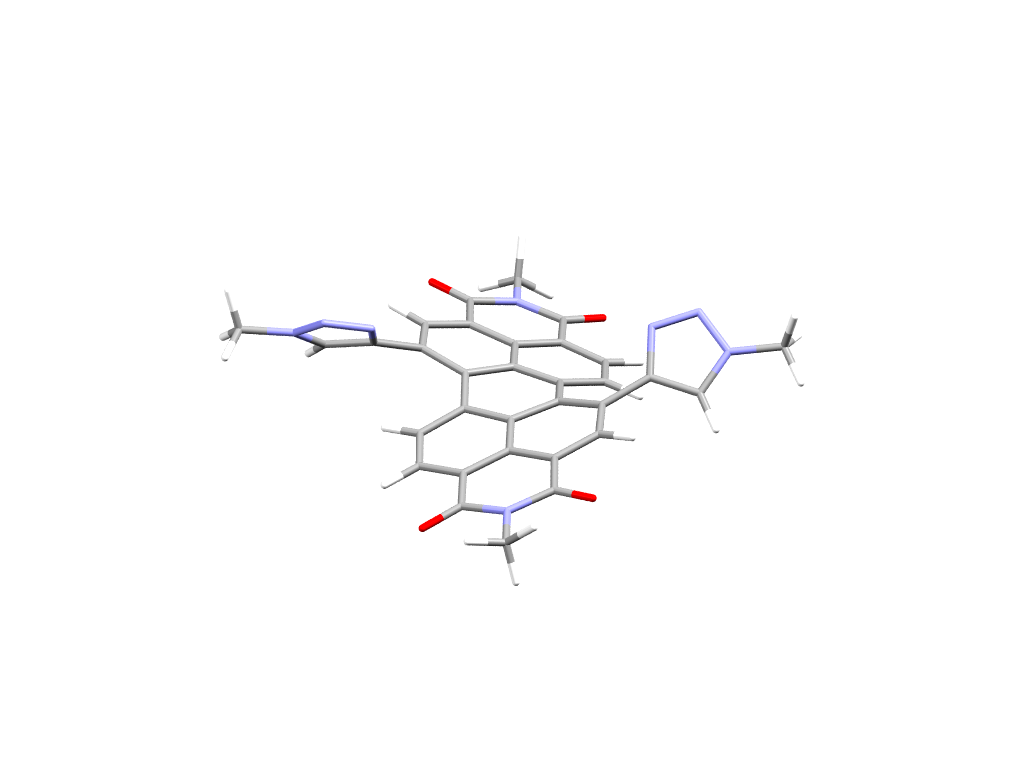


**Supplementary Figure 5‑1:** Optimised structure of 1,7-ditriazole PDI by means of density functional theory (B97-3c, def2-TZVP).

### Comparison of Torsion Angles between ditriazole and ditriazolium PDIs from optimised DFT


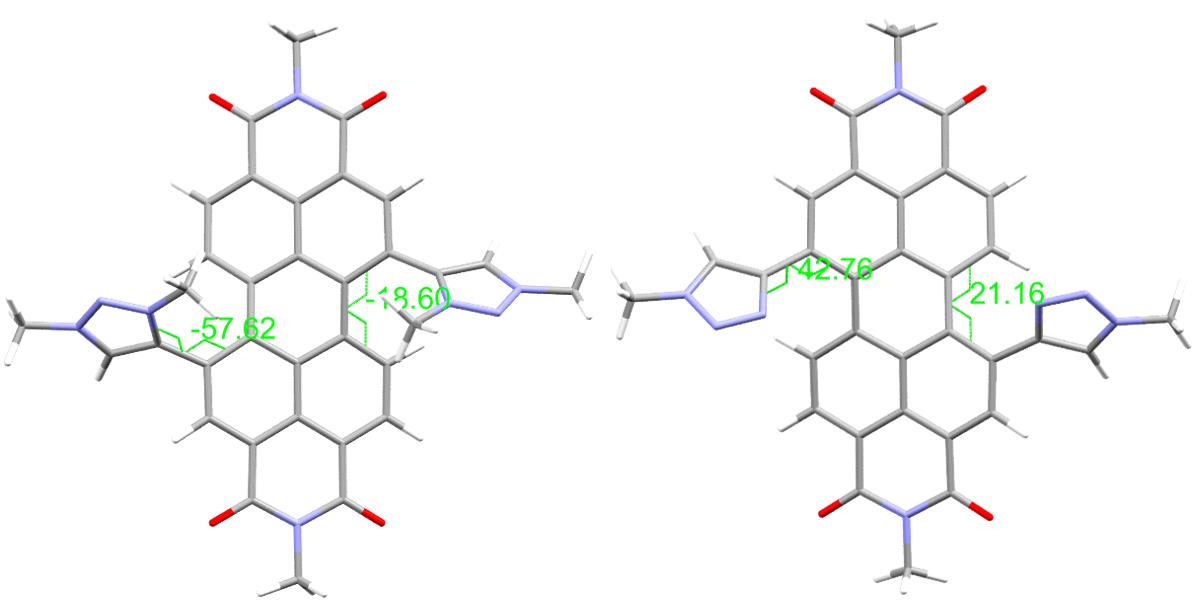


**Supplementary Figure 5‑2:** Torsion angles of structures of 1,7-ditriazolium (left) and 1,7-ditriazole (right), optimised by means of DFT using the B97-3c basis set and the def2-TZVP functional. Torsion angles were measured in Mercury.

### Conformational Landscape of 1,7-ditriazolium PDI 4a


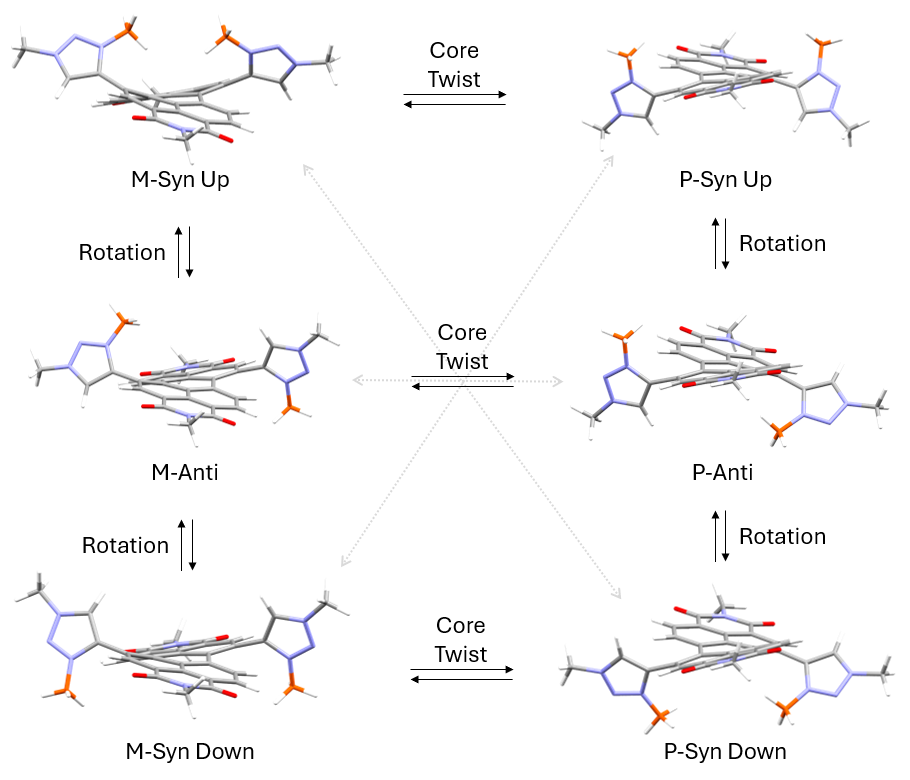


**Supplementary Figure 5‑3:** Conformational landscape of ditriazolium PDI **4a**. Conformers were obtained from the CREST conformer/rotamer search. Syn/anti refers to the relative positioning of the triazolium N-Me groups highlighted in orange. Dashed grey arrows indicate enantiomeric pairs.

### Conformational Landscape of 1,6-ditriazolium PDI 4b


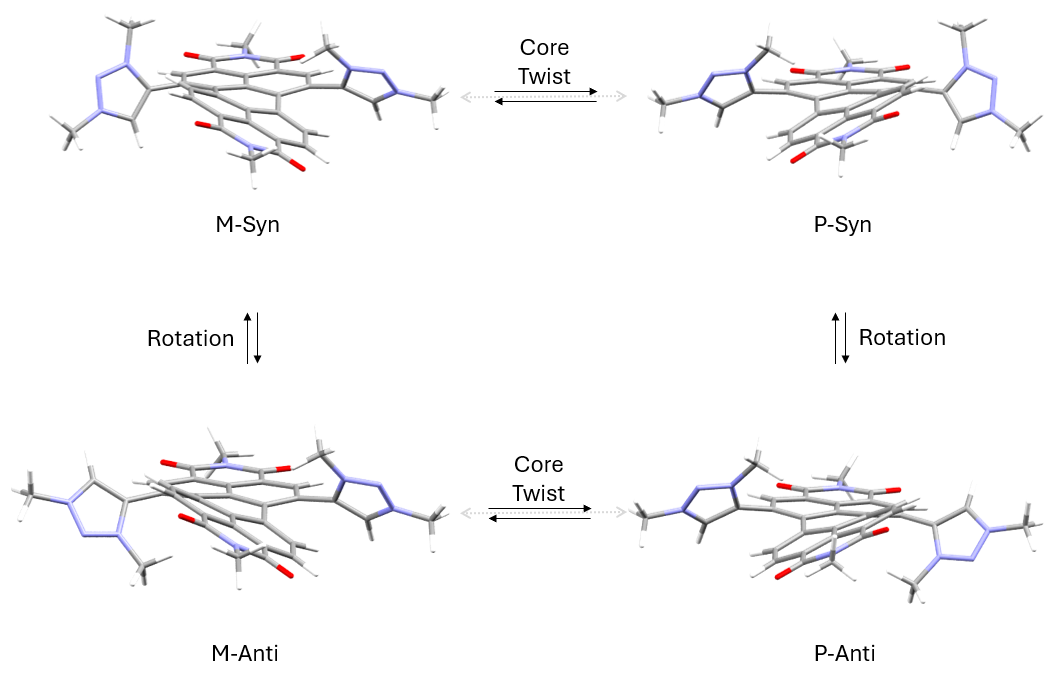


**Supplementary Figure 5‑4:** Conformational landscape of ditriazolium PDI **4b**. Conformers were obtained from the CREST conformer/rotamer search. Syn/anti refers to the relative positioning of the triazolium N-Me groups. Dashed grey arrows indicate enantiomeric pairs.

### Conformer Search using CREST

A conformer search for 1,7-ditriazolium-PDI **4a** was performed using the combination of the CREST code^[8]^ and the GFN2-xTB semiempirical tight-binding method,^[9,10]^ with implicit solvation in MeCN using the analytical linearized Poisson-Boltzmann (ALPB) model. This provided several conformers close in energy, with a *syn*-isomer with both methyl-groups pointing towards each other as the lowest predicted conformer.


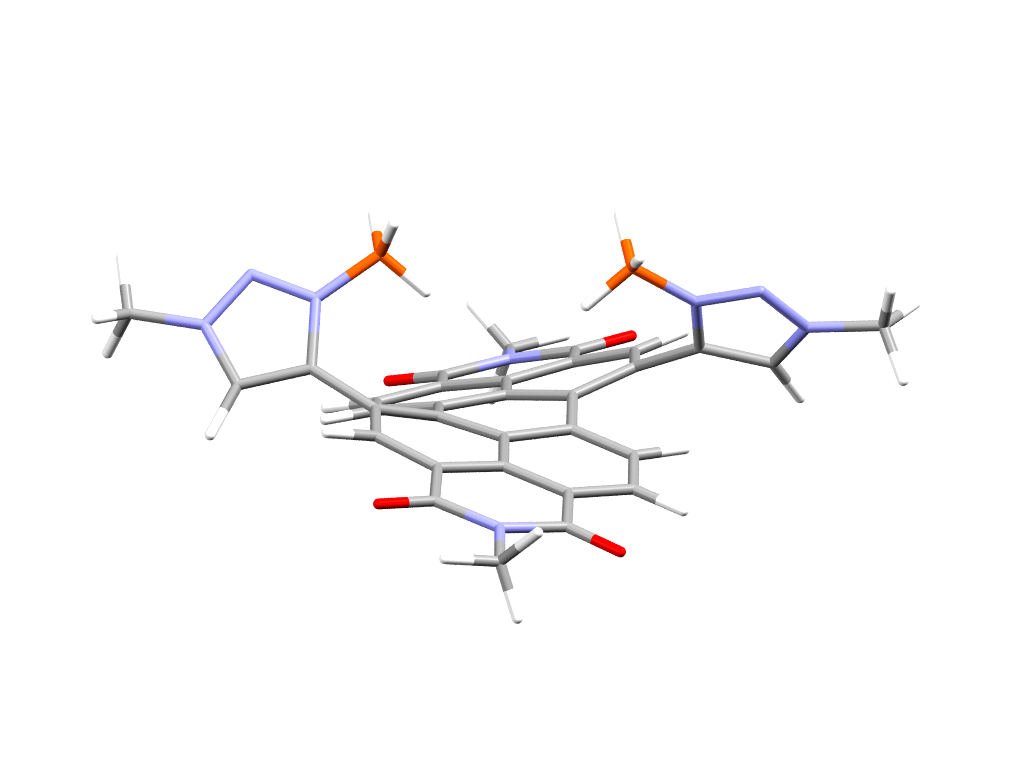


**Supplementary Figure 5‑5**: Lowest energy conformer for the 1,7-ditriazolium PDI **4a** as predicted by CREST. Triazolium N-Me groups are coloured in orange to distinguish them from the simplified C8 chains.

Similarly, for the 1,6-ditriazolium PDI **4b**, a conformer search was performed using the combination of the CREST code^[8]^ and the GFN2-xTB semiempirical tight-binding method,^[9,10]^ using implicit solvation in MeCN using the analytical linearized Poisson-Boltzmann (ALPB) model. This provided a lowest energy conformer showing a twisted PDI core with anti-pointing methyl groups.


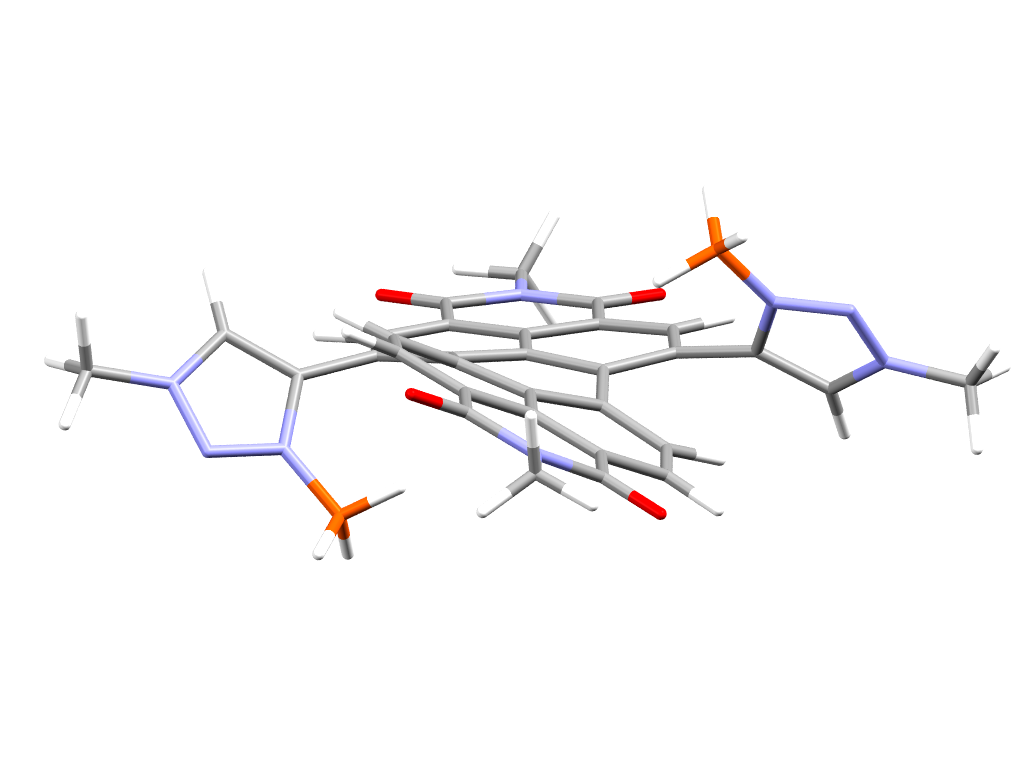


**Supplementary Figure 5‑6:** Lowest energy conformer for the 1,6-ditriazolium PDI **4b** as predicted by CREST. Triazolium N-Me groups are coloured in orange to distinguish them from the simplified C8 chains.

### Determination of energy difference between syn and anti-isomers by DFT

Representative conformers of a *syn/anti* isomer pair of the same helicity found using CREST for the 1,7-isomer **4a** were reoptimized by means of density functional theory using the B97-3c basis set by Grimme and co-workers,^[6]^ and the def2-TZVP functional.^[7]^ This yielded an energy difference of ΔG = 1.3 kJ/mol in favour of the *syn*-conformer. Their energies were then calculated at a higher level of theory (B97-3c, def2-QZVP), with the D3BJ dispersion correction^[11]^ with implicit solvation in MeCN using the conductor-like polarizable continuum model (CPCM).^[12]^ From this, the Gibbs free energy difference between *syn*- and *anti*-conformers was calculated to be ΔG = 3.1 kJ/mol with the *syn*-isomer lower in energy, matching prediction by CREST.

Analogously, the *syn*- and *anti*-isomers found using CREST for the 1,6-isomer **4b** were optimised by DFT (B97-3c, def2-TZVP) and their energies calculated at a higher level of theory (B97-3c, def2-QZVP, D3BJ, CPCM solvation in MeCN) to yield an energy difference of
ΔG = 3.5 kJ/mol in favour of the anti-isomer, again matching prediction by CREST.

DFT optimised geometries are provided as .xyz files in the supporting files.


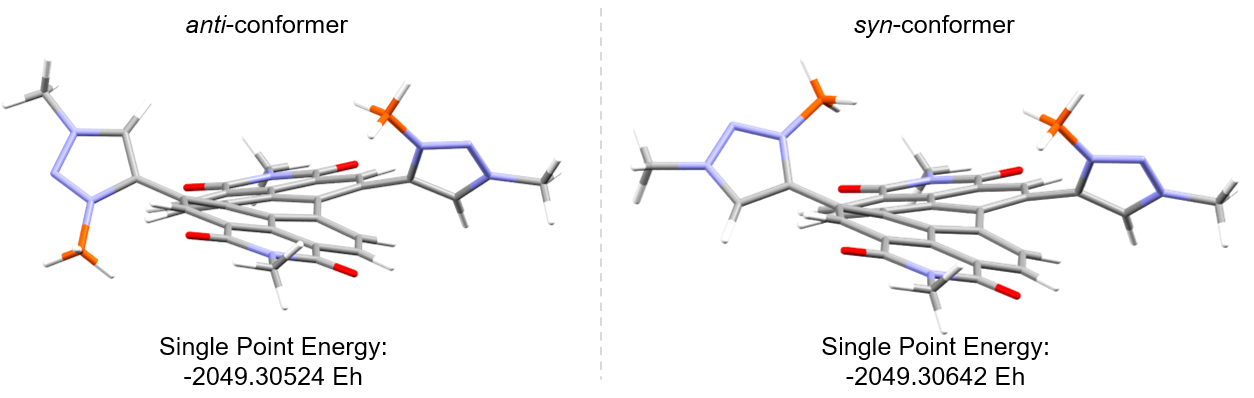


**Supplementary Figure 5‑7:** DFT-optimised structures of anti- and syn-conformers of 1,7-ditriazolium PDI and their respective single point energies. Triazolium N-Me groups are coloured in orange to distinguish them from the simplified C8 chains.


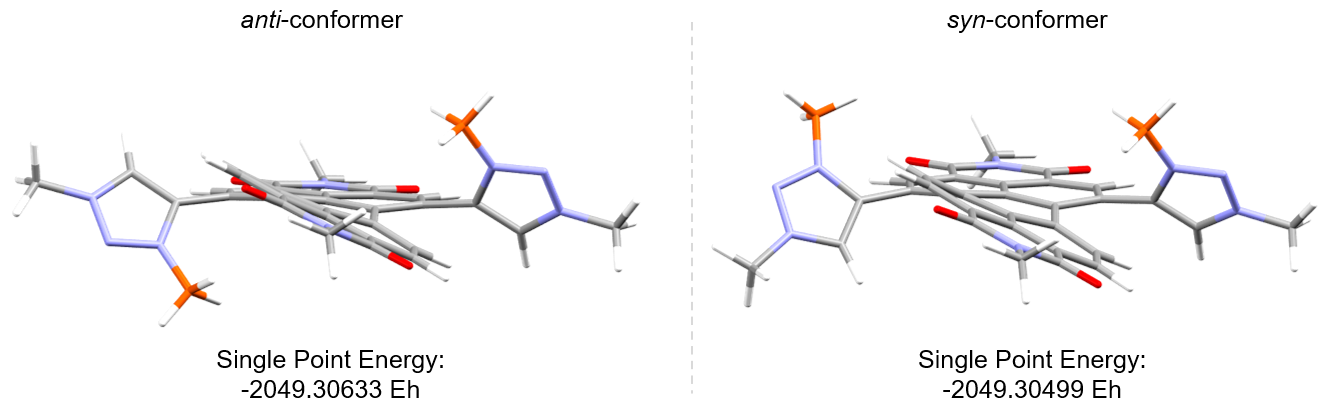


**Supplementary Figure 5‑8:** DFT-optimised structures of anti- and syn-conformers of 1,6-ditriazolium PDI and their respective single point energies. Triazolium N-Me groups are coloured in orange to distinguish them from the simplified C8 chains.

### Transition state calculation of M/P interconversion using NEB-CI

The transition state (TS) for M/P-isomer interconversion was computed using the r^2^SCAN-3C composite method,^[13]^ the def2-TZVP basis set,^[7]^ and the conductor-like polarizable continuum model (CPCM)^[12]^ for implicit solvation in MeCN. A Nudged Elastic Band-Climbing Image (NEB-CI)^[14,15]^ model was employed for TS determination of helical interconversion between the *M*- and *P*-isomers with methyl groups pointing syn (as it has been found to be lower in energy), using representative examples of *M*- and *P*-helical isomers obtained from CREST, which were subsequently reoptimized by DFT (B97-3c functional and def2-TZVP basis set) as starting points. The results from this calculation made evident that there is an energy difference between the two syn-isomers and yielded the expected planar high-energy state. The trajectory is shown below (**Supplementary Figure 5-9**) and the optimised geometries of the two syn-isomers as well as the final TS from NEB-CI are available as .xyz files in the supporting files.


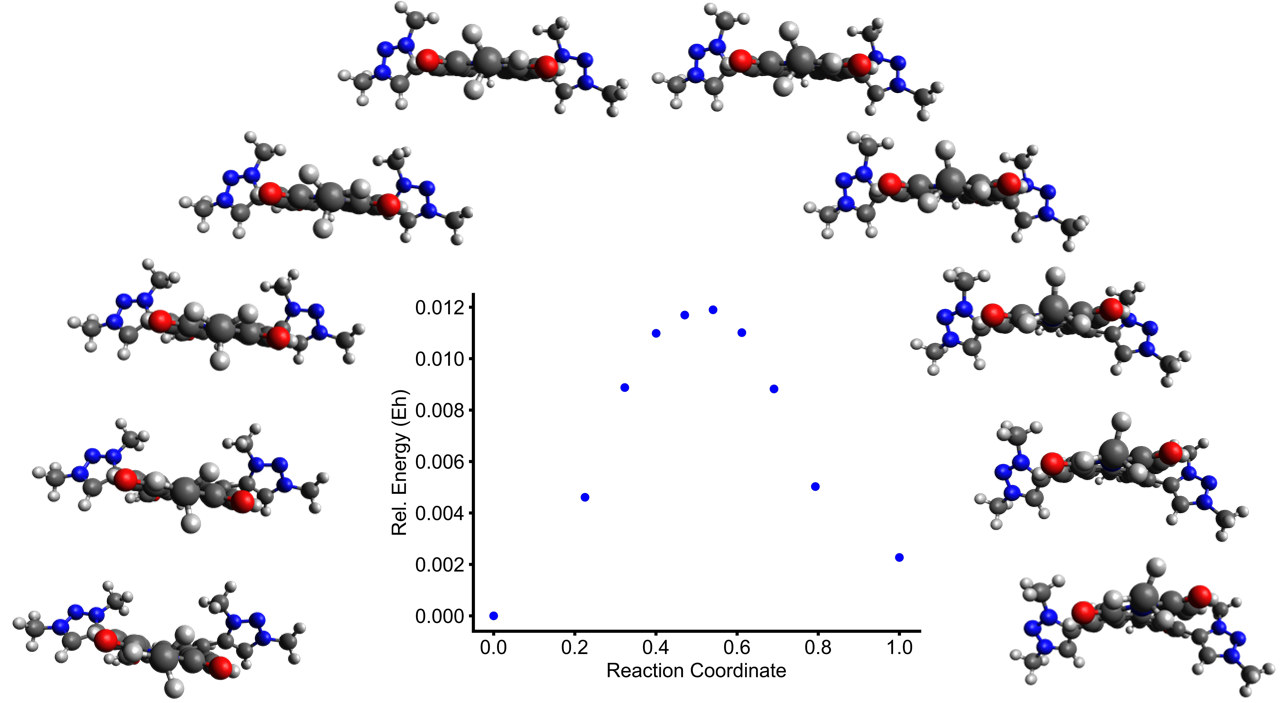


**Supplementary Figure 5‑9:** Reaction coordinate as predicted by NEB-CI with several steps along the coordinate, including the highest-energy state, and their relative energies.

### Calculation of Energy Barrier of ditriazolium interconversion

Energy barriers were calculated through calculation of single point energies of B97-3c/def2-TZVP geometry-optimised structures of the two syn-isomers, as well as the single-point energy of the TS as predicted by NEB-CI at various levels of theory^[5]^ using the CPCM model of implicit solvation for MeCN, the RI-JK approximation with a def2/JK auxiliary basis set and the D3BJ dispersion correction.^[11]^ As observed in NEB-CI, the *syn*-isomers showed small differences in energy at all levels of theory, as they are not enantiomers. The energy barrier for interconversion was therefore calculated from the average energy of the two *syn*-isomers to the TS. The calculations yielded a barrier height of ~35-36 kJ/mol between the *syn*-isomer average and the TS at the PWPB95 double-hybrid-meta-GGA density functional^[16]^ level of theory with different basis sets.

**Supplementary Table 5‑1:** Energies of M/P-interconversion at different levels of theory.

| **Functional** | **B3LYP** | **B97-3c** | **wB97X-D3** | **PWPB95** | **PWPB95** | **PWPB95** |
| --- | --- | --- | --- | --- | --- | --- |
| **Basis Set** | **ZORA-def2-TZVP** | **def2-QZVP** | **def2-TZVP** | **def2-TZVP** | **ZORA-def2-TZVP** | **def2-QZVP** |
| **ΔE (Syn/Syn)**  **kJ/mol** | 1.24 | 6.25 | 6.60 | 6.64 | 6.66 | 6.82 |
| **ΔE (Syn/TS)**  **kJ/mol** | 23.34 | 33.21 | 30.56 | 34.97 | 35.18 | 36.47 |


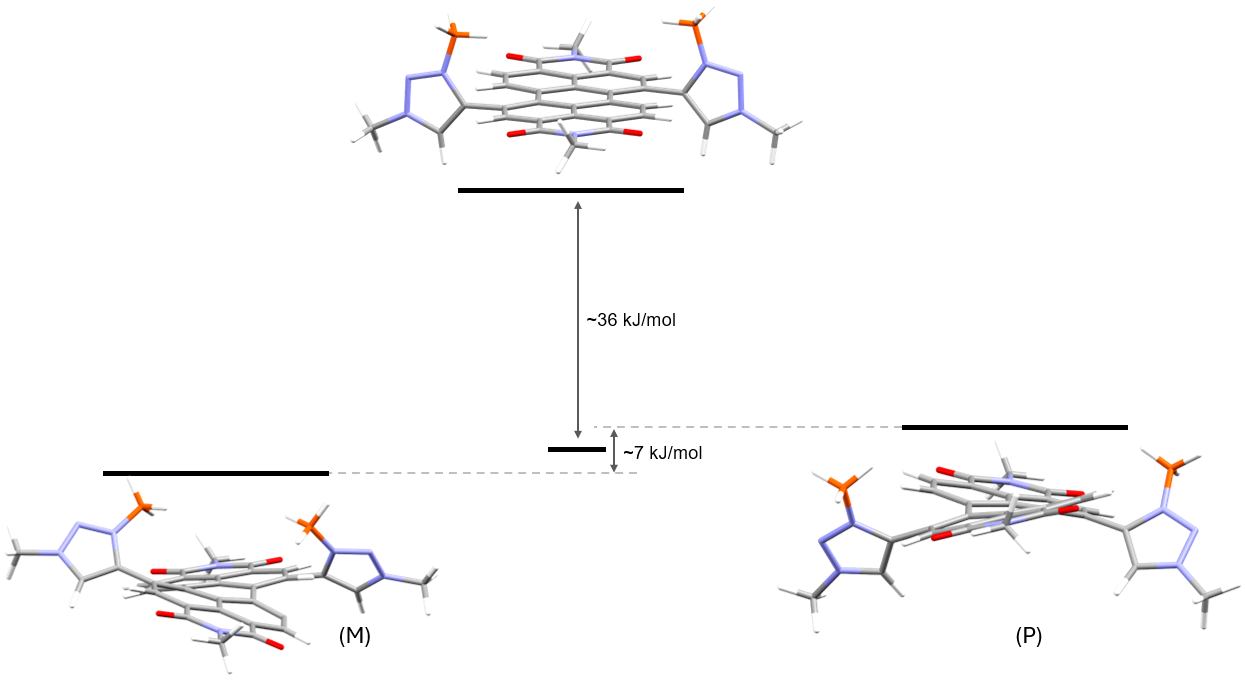


**Supplementary Figure 5‑10**: Energy differences between syn-isomers and their average energy difference to the TS. Triazolium N-Me groups are coloured in orange to distinguish them from the simplified C8 chains.

### Determination of aggregation types of 1,7-bistriazolium PDIs

Possible aggregate geometries were predicted via a conformer search for 1,7-ditriazolium-PDI **4a** using the combination of the CREST code^[8]^ and the GFN2-xTB semiempirical tight-binding method,^[9,10]^ using implicit solvation in PhMe using the ALPB model.^[10]^ The conformer search predicted an H-type aggregate of two *syn*-facing PDIs as the lowest sensible structure. The geometry of this, as well as *syn/anti* and *anti/anti* pairs were then optimised by means of DFT using the B97-3c functional with a def2-TZVP basis set and implicit solvation in PhMe *via* the CPCM model. The resulting single point energies yield an energetic ordering of *syn/syn* < *syn/anti* < *anti/anti*, as is anticipated by the loss of hydrogen bonds. The results are summarised in **Supplementary Table 5-2**.


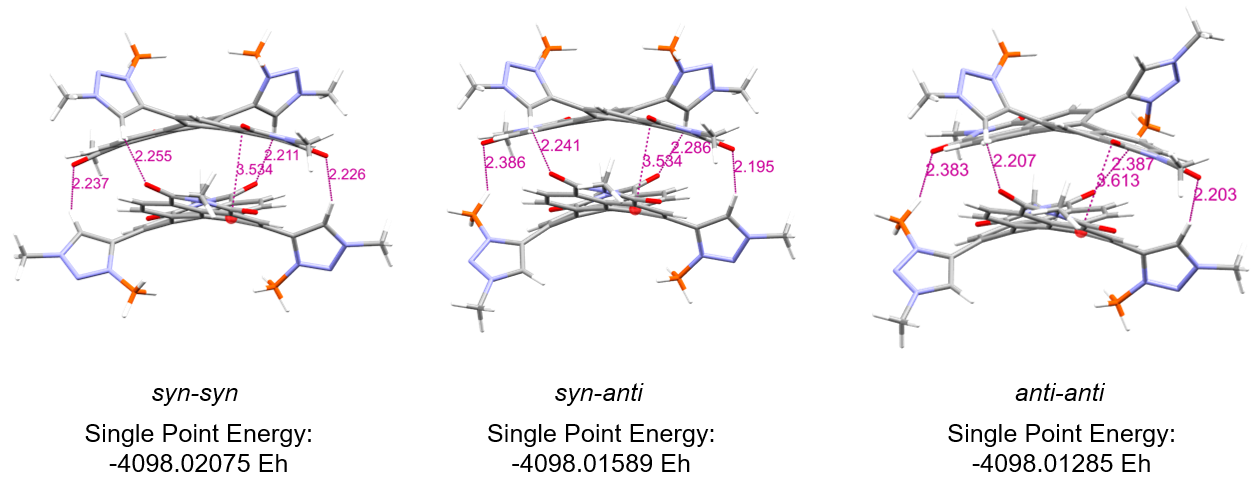


**Supplementary Figure 5‑11:** Conformers of dimers of compound **4a** in PhMe optimised by DFT. Triazolium N-Me groups are coloured in orange to distinguish them from the simplified C8 chains.

**Supplementary Table 5‑2:** Energies of H-type dimer structures as predicted by DFT (B97-3c/def2-TZVP) and their energy differences to the lowest energy conformer (kJ/mol).

| Index | Dimer  Species | Energy/Eh | Difference to *syn-syn* (kJ/mol) | Triazolium Hydrogen Bonds |
| --- | --- | --- | --- | --- |
| 1 | *syn/syn* | -4098.02075 | 0 | 4 |
| 2 | *syn/anti* | -4098.01589 | +12.75 | 3 |
| 3 | *anti/anti* | -4098.01285 | +20.74 | 2 |

### Prediction of possible interactions of (−)-BINOL-phosphate anion with ditriazolium PDI by CREST

Possible aggregate geometries for a 2:1 complex of (−)-BINOL-phosphate (the structure of which was taken from the crystal structure) with 1,7-ditriazolium PDI (DFT-optimised structure) were predicted using the combination of the CREST code^[8]^ and the GFN2-xTB semiempirical tight-binding method,^[9,10]^ using implicit solvation in PhMe using the ALPB model.^[10]^ This yielded a homochiral aggregate between the chiral anion and the PDI as the lowest energy conformer. This lowest energy conformer was subsequently optimised by DFT (B97-3c, def2-TZVP).


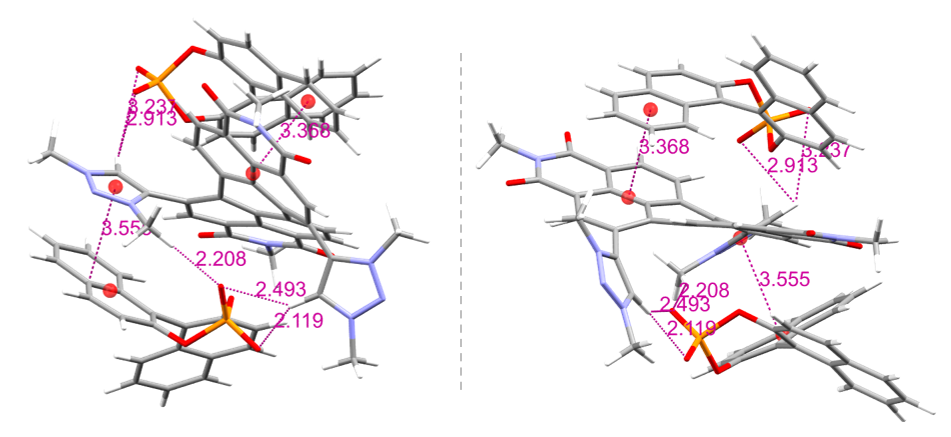


**Supplementary Figure 5‑12:** Front and side view of the lowest energy conformer predicted by CREST after DFT optimisation. Clear π-stacking interactions and hydrogen bonds are predicted.

### Prediction of circular dichroism of the (−)-BINOL-phosphate-PDI complex by TD-DFT

Vertical excitation and circular dichroism spectra of the (−)-BINOL-phosphate complex were predicted using TD-DFT in PhMe. These calculations employed the TD-ωB97x^[17]^ functional, the def2-SVP basis-set,^[7]^ and the CPCM solvation model and were performed on top of the B97-3c optimised structure. TD-DFT calculations were performed in ORCA 6.0.0.

**Supplementary Table 5‑3:** Predicted UV-vis absorption spectrum and rotary strengths in the CD spectrum of the 1,7-ditriazolium PDI-(−)-BINOLphosphate complex in PhMe by time dependent-DFT.

| **Transition** | **Wavelength/nm** | **Oscillator Strength** | **Rotary Strength** |
| --- | --- | --- | --- |
| 1 | 484.7 | 0.6265 | -57.225 |
| 2 | 414 | 0.0213 | -3.7305 |
| 3 | 356.9 | 0.0091 | 21.2195 |
| 4 | 346.8 | 0.0031 | -1.7110 |
| 5 | 334.2 | 0.0139 | -91.3329 |
| 6 | 321.8 | 0.1153 | 27.0417 |
| 7 | 316.4 | 0.0274 | 83.4559 |
| 8 | 307.4 | 0.0089 | 44.7223 |
| 9 | 300.6 | 0.0289 | 21.7590 |
| 10 | 299.1 | 0.0106 | -18.6937 |


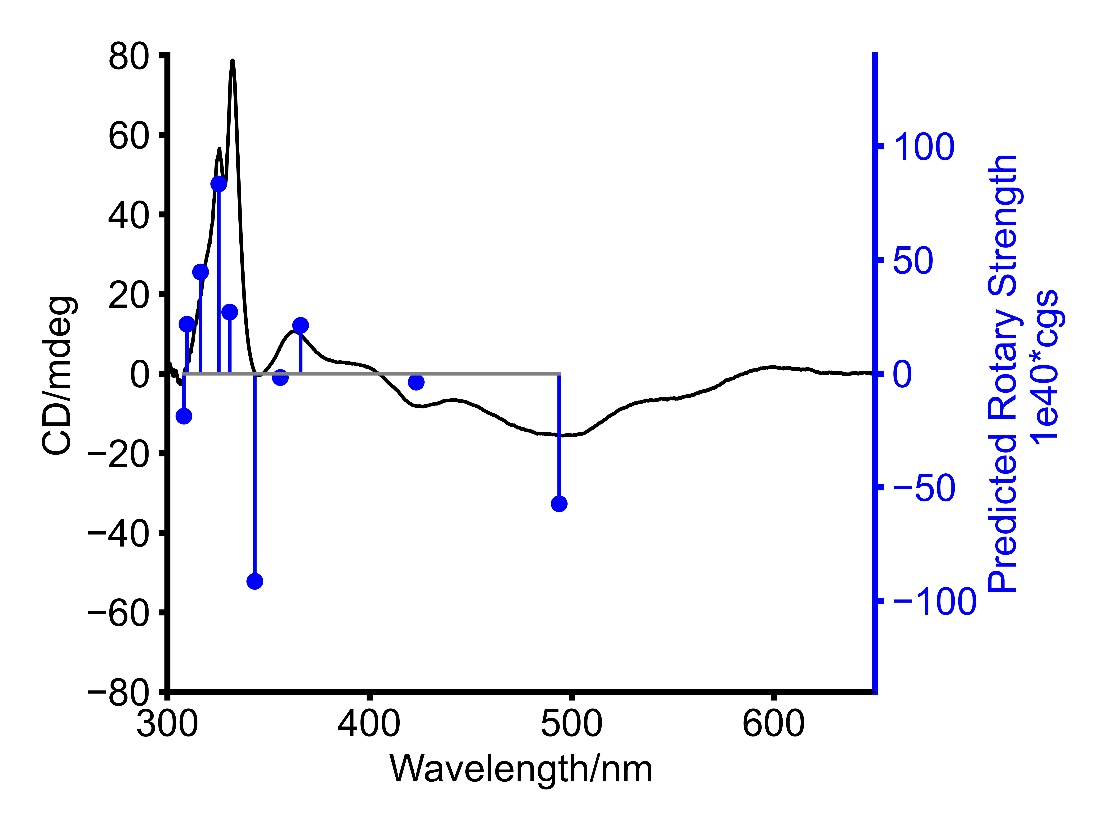


**Supplementary Figure 5‑13:** Predicted CD Spectrum of the complex of 1,7-ditriazolium-PDI with (−)-BINOLphosphate in PhMe against recorded data. Predicted vertical transitions were shifted by +9 nm, since predicted CD spectra are blue-shifted, which is a side-effect of the range-separated functional used.

# NMR Spectra

### 1,7-Br_2_-C5-PDI 1a

(400 MHz, CDCl_3_)


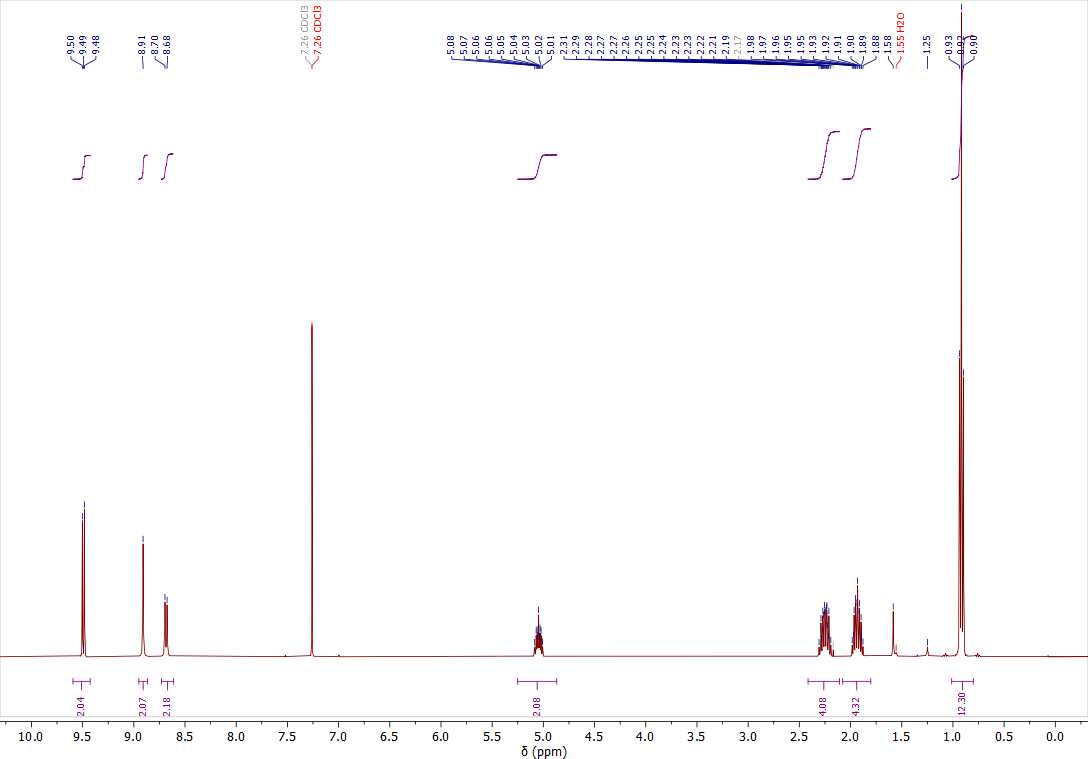


### 1,6-Br_2_-C5-PDI 1b

(400 MHz, CDCl_3_)


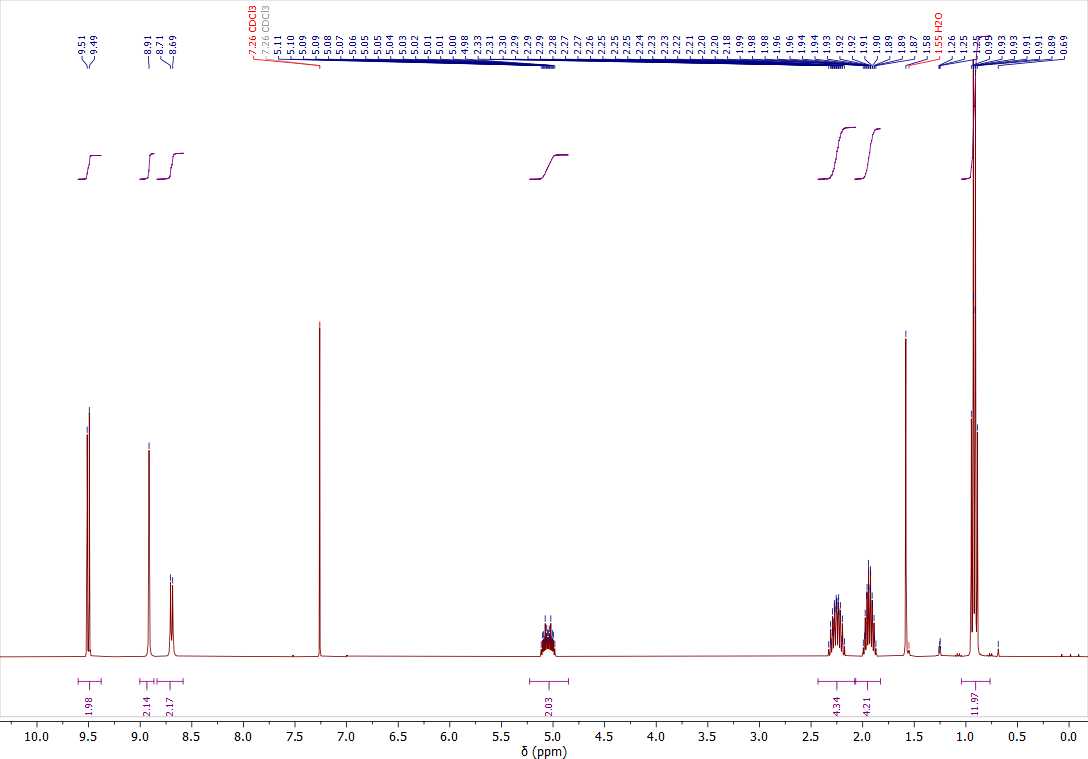


### 1,7-di(Trimethylsilylacetylene)-C5-PDI 2a

(400 MHz, CDCl_3_)

### 1,6-di(Trimethylsilylacetylene)-C5-PDI 2b

^1^H NMR (400 MHz, CDCl_3_)

^13^C NMR (101 MHz, CDCl_3_)

### 1,7-di(Octyltriazole)-C5-PDI 3a

^1^H NMR (400 MHz, CDCl_3_)

^13^C NMR (101 MHz, CDCl_3_)

### 1,6-di(Octyltriazole)-C5-PDI 3b

^1^H NMR (400 MHz, CDCl_3_)

^13^C NMR (101 MHz, CDCl_3_)

### 1,7-di(Octylmethyltriazolium)-C5-PDI Iodide S1

^1^H NMR (MeCN-*d_3_*, 298 K, 300 MHz)

^1^H NMR (MeCN-*d_3_*, 101 MHz)

### 1,7-di(Octylmethyltriazolium)-C5-PDI PF_6_ 4a

1H NMR (400 MHz, 348 K, CDCl_3_)

^13^C NMR (101 MHz, 298 K, CDCl_3_)

^19^F NMR (377 MHz, 298 K, MeCN-*d_3_*)

### 1,6-di(Octylmethyltriazolium)-C5-PDI PF_6_ 4b

1H NMR (400 MHz, 348 K, CDCl_3_)

^13^C NMR (101 MHz, 298 K, CDCl_3_)

### 1,7-di(Octyltriazole)-C5-CDI S2

^1^H NMR (400 MHz, TCE-*d_2_*)

^13^C NMR (400 MHz, TCE-*d_2_*, 373 K)

### 1,7-Br_2_-C11-PDI S3

^1^H NMR (300 MHz, CDCl_3_)

### 1,7-di(trimethylsilylacetylene)-C11-PDI S4

^1^H NMR (400 MHz, CDCl_3_)

### 1,7-di(Octyltriazole)-C11-PDI 5

^1^H NMR (400 MHz, CDCl_3_)

^13^C NMR (101 MHz, CDCl_3_)

### 1,7-di(Octyltriazole)-C11-CDI 6

^1^H NMR (400 MHz, CDCl_3_)

^13^C NMR (126 MHz, CDCl_3_)

### Tetrabutylammonium (+)-BINOL-phosphate 7a

^1^H NMR (300 MHz, CDCl_3_)

### Tetrabutylammonium (−)-BINOL-phosphate 7b

^1^H NMR (300 MHz, CDCl_3_)

### Variable Temperature NMR Spectra of Compound 4a in MeCN-*d_3_*

348 K

338 K

328 K

318 K

308 K

298 K

**Supplementary Figure 6‑1:** Variable Temperature NMR of compound **4a** in MeCN-d_3_

### Variable Temperature NMR Spectra of Compound 4b in MeCN-*d_3_*

348 K

298 K

308 K

318 K

328 K

338 K

**Supplementary Figure 6‑2**: Variable Temperature NMR of compound **4b** in MeCN-d_3_

### Comparison of 1,7-bistriazolium-PDI 4a in various NMR solvents

MeOH-*d_4_*

CDCl_3_

PhMe-*d_8_*

MeCN-*d_3_*

**Supplementary Figure 6‑3**: NMR spectra of 1,7-ditriazolium PDI **4a** in various solvents at room temperature.

**Supplementary Figure 6‑4:** NOESY/EXSY Spectrum of 1,7-ditriazolium PDI **4a** in PhMe at 25 °C.

**Supplementary Figure 6‑5:** NMR of 1,7-ditriazolium PDI **4a** in PhMe-d_8_ at 25 °C (bottom) and 80 °C (top).

353 K

343 K

333 K

323 K

313 K

303 K

298 K

**Supplementary Figure 6‑6:** Variable temperature NMR of 1,7-ditriazolium-C5 PDI **4a** in PhMe-d_8_ after deuterium exchange in MeOH-d_4_.

**Supplementary Figure 6‑7:** Zoom of the aromatic region of 1,7-bistriazolium PDI in PhMe-d_8_ at room temperature, and integration of major and minor species.

**Supplementary Figure 6‑8:** Comparison of Aromatic Region of 1,7-ditriazolium PDI **4a** in PhMe-d_8_ before and after deuterium exchange. The change in integration and change in shape for the peak at 9.05 ppm is indicative of a hidden triazolium proton peak.

### Additions of TBACl to 4a in MeCN-*d_3_*


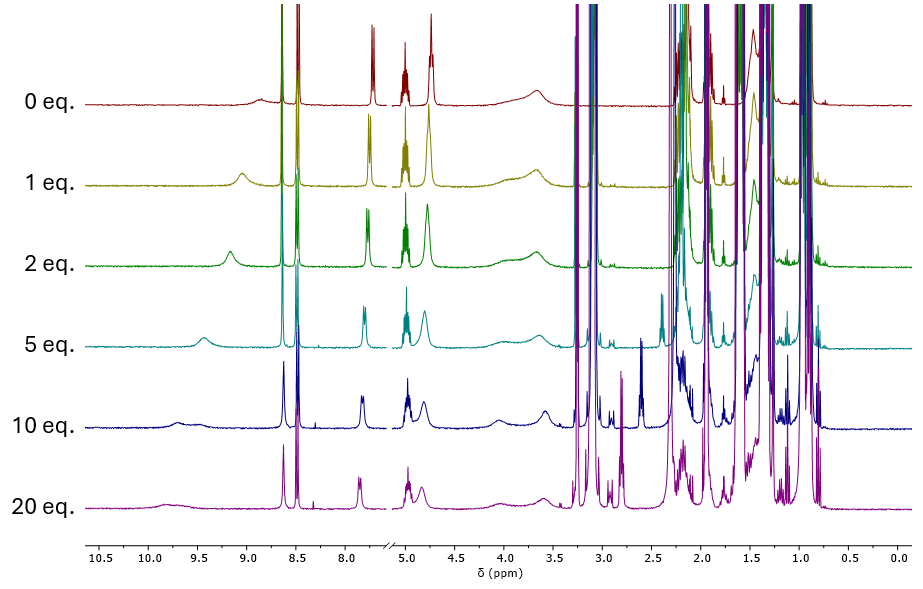


**Supplementary Figure 6‑9**: Changes in NMR spectrum of compound **4a** in MeCN-d_3_ upon addition of TBACl (up to 20 eq.)

### Variable Temperature NMR of compound 4a with 20 equivalents of TBACl in MeCN-*d_3_*

348 K

298 K

308 K

318 K

328 K

338 K

**Supplementary Figure 6‑10**: Variable Temperature NMR of 1,7-ditriazolium PDI **4a** with 20 equivalents of TBACl in MeCN-d_3_.

5 Eq.

2 Eq.

1 Eq.

0 Eq.

**Supplementary Figure 6‑11:** NMR titration of **4a** with (−)-BINOL-phosphate in PhMe-d_8_ at 353 K**.**

5 Eq.

2 Eq.

1 Eq.

0 Eq.

**Supplementary Figure 6‑12:** NMR titration of **4a** with (−)-BINOL-phosphate in PhMe-d_8_ at 298 K**.**

**Supplementary Figure 6‑13:** NMR spectrum of 1,7-ditriazolium-PDI **4a** with 5 eq. (−)-BINOL-phosphate after deuterium exchange in MeOD-d_4_ (teal trace), compared to the protonated spectrum (maroon trace).

**Supplementary Figure 6‑14:** Estimation of %de from NMR integration of signals corresponding to proton H_b_ after ablative baseline correction. From the integral ratio, a de of 19% was calculated.

### Calculation of ∆𝐺^‡^ of interconversion from variable temperature NMR data

Barrier heights of interconversion between syn- and anti-Me rotamers of 1,7- and 1,6-PDIs **4a/b** as well as for **4a**+20 eq. TBACl were calculated using the Eyring equation as follows:

$${\Delta G}_{eT_{c}}^{\boldsymbol{\ddagger}}=RT_{c}\left[ \ln\left( \frac{k_{B}T_{c}}{h} \right)-ln(k_{eT_{c}}) \right]$$

With $T_{c}$ being the temperature of coalescence (in K), and the rate constant of interconversion at the coalescence point $k_{eT_{c}}$ being defined as

$$k_{eT_{c}}=\frac{\pi\Delta\nu}{\sqrt{2}}$$

With $\Delta\nu$ being the difference in chemical shifts of the two methyl groups on the triazolium in Hz (chemical shifts ~ 4 ppm).

| **Index** | **Species** | $\boldsymbol{T}_{\boldsymbol{c}}$ **/ K** | $\boldsymbol{\Delta\nu}$ **/ ppm** | ${\boldsymbol{\Delta}\boldsymbol{G}}_{\boldsymbol{e}\boldsymbol{T}_{\boldsymbol{c}}}^{\boldsymbol{\ddagger}}$ **/ kJ/mol** |
| --- | --- | --- | --- | --- |
| 1 | **4a** | 308 | 0.22 | 62 |
| 2 | **4b** | 308 | 0.36 | 61 |
| 3 | **4a**  + 20 eq. TBACl | 308 | 0.44 | 60 |

Similarly, barriers for the interconversion of *syn* and *anti*, as well as the *M/M* and *P/P* isomers were calculated from the NMR in PhMe-*d_8_* (**Supplementary Figure 6-6**):

| **Index** | **Environment** | $\boldsymbol{T}_{\boldsymbol{c}}$ **/ K** | $\boldsymbol{\Delta\nu}$ **/ ppm** | ${\boldsymbol{\Delta}\boldsymbol{G}}_{\boldsymbol{e}\boldsymbol{T}_{\boldsymbol{c}}}^{\boldsymbol{\ddagger}}$ **/ kJ/mol** |
| --- | --- | --- | --- | --- |
| 1 | H_a_ (8 ppm) | 323 | 0.176 | 66 |
| 2 | H_g_ (3.16 ppm)_ | 323 | 0.18 | 66 |
| 3 | H_e_ (4.35-4.8 ppm) | 343 | 0.25 | 69 |

# Single Crystal X-Ray Diffraction

Crystals for tetrabutylammonium (+)-BINOL*-*phosphate **7a** and tetrabutylammonium (−)-BINOL*-*phosphate **7b** were prepared by slow evaporation of a solution of **7a/b** in toluene. The obtained crystals were measured on an Agilent SuperNova single crystal X-ray diffractometer with dual wavelength microfocus X-ray source and an Atlas CCD detector. Data were collected at
100 K through the use of an Oxford Cryosystems cryostream device using Cu K_α_ radiation. Data were collected and processed using CrysAlisPro 1.171.43. The structure was solved by direct methods using ShelXT^[18]^ and refined with ShelXL^[19]^ using a least squares method. Olex2 was used as the solution, refinement and analysis program.^[20]^ Hydrogen atoms were placed geometrically and refined using a riding model. A small amount of disorder was found in tetrabutylammonium (+)-BINOL-phosphate, which could not be accurately modelled.

**Tetrabutylammonium (+)-BINOL*-*phosphate 7a**

**Crystal Data** for C_36_H_50_NO_5_P (*M*=607.74 g/mol): monoclinic, space group P2_1_ (no. 4), *a* = 10.9554(2) Å, *b* = 18.2134(3) Å, *c* = 17.2069(3) Å, *β* = 104.582(2)°, *V*= 3322.78(10) Å^3^, *Z* = 4, *T* = 100.15 K, μ(CuKα) = 1.064 mm^-1^, *Dcalc* = 1.215 g/cm^3^, 62013 reflections measured (7.192° ≤ 2Θ ≤ 136.49°), 12107 unique (*R*_int_ = 0.0438, R_sigma_ = 0.0303) which were used in all calculations. The final *R*_1_ was 0.0528 (I > 2σ(I)) and *wR*_2_ was 0.1493 (all data).
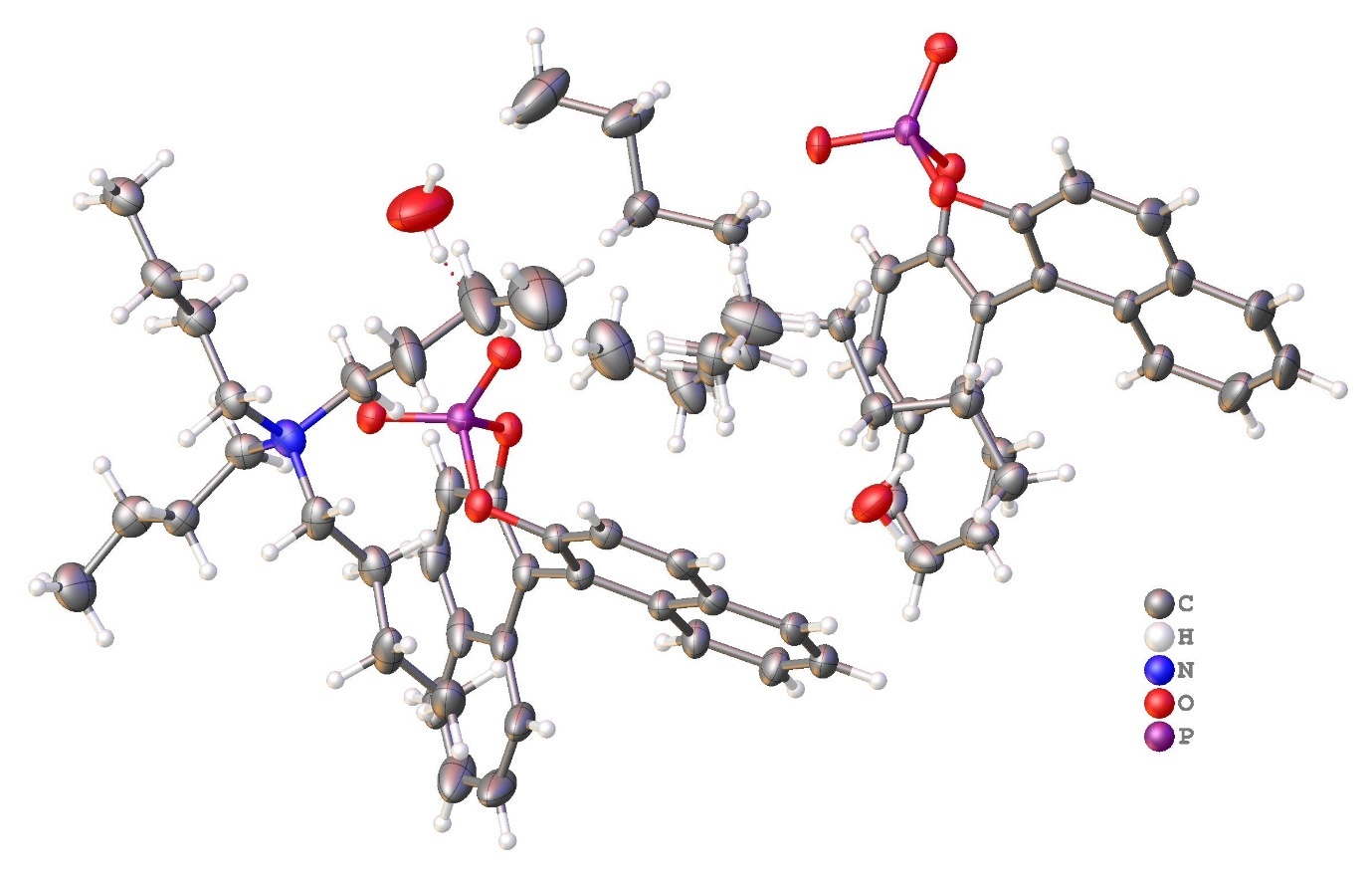


**Supplementary Figure 7‑1**: Asymmetric unit of the crystals of tetrabutylammonium (+)-BINOL-phosphate. Thermal ellipsoids show 50% probability.

**Tetrabutylammonium (−)-BINOL*-*phosphate 7b**

**Crystal Data** for C_36_H_50_NO_5_P (*M*=607.74 g/mol): orthorhombic, space group P2_1_2_1_2_1_ (no. 19), *a* = 20.1735(2) Å, *b* = 20.1881(2) Å, *c* = 31.9661(6) Å, *V*= 13018.7(3) Å^3^, *Z* = 16,
*T* = 100.00(10) K, μ(Cu Kα) = 1.087 mm^-1^, *Dcalc* = 1.240 g/cm^3^, 72742 reflections measured (7.054° ≤ 2Θ ≤ 144.254°), 24138 unique (*R*_int_ = 0.0500, R_sigma_ = 0.0546) which were used in all calculations. The final *R*_1_ was 0.0431 (I > 2σ(I)) and *wR*_2_ was 0.1003 (all data).
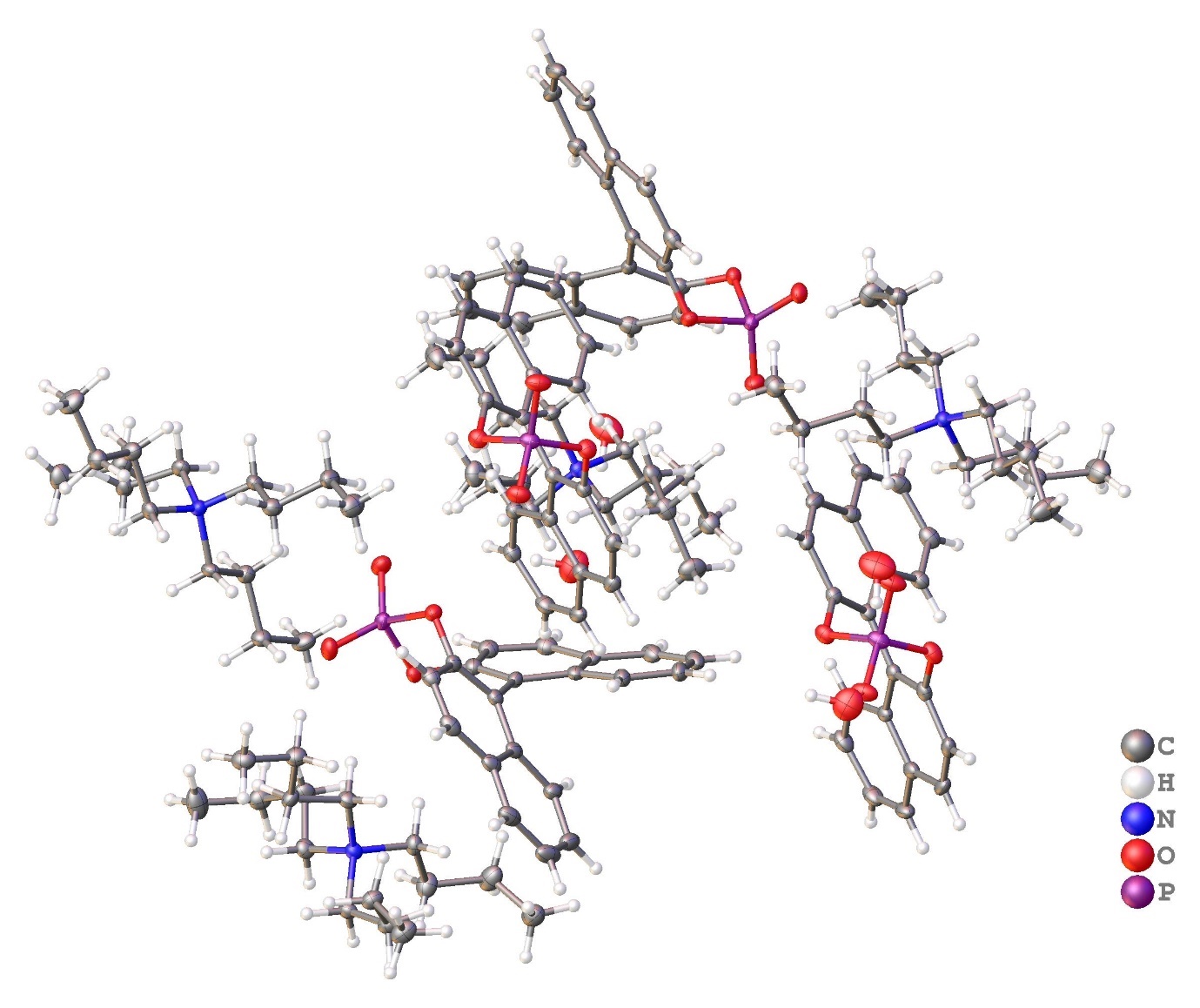


**Supplementary Figure 7‑2:** Asymmetric unit of the crystals of tetrabutylammonium (−)-BINOL-phosphate. Thermal ellipsoids show 50% probability.

### 1,7-di(Octyltriazole)-C5-PDI 3a

Single crystals of 1,7-bis(octyltriazole)-C5-PDI **3a** were prepared by slow evaporation of a solution of **3a** in toluene. Single crystal X-ray diffraction experiments for those crystals were performed by the UK EPSRC National Crystallography Service at the University of Southampton^[21]^ on a Rigaku 007HF diffractometer with HF Varimax confocal mirrors, an UG2 goniometer and HyPix 6000HE detector at 100 K using Cu K_α_ radiation and processed using CrysAlisPro 1.171.43. The molecule is located on an inversion centre located in the centre of the fused ring system such that only half is crystallographically unique. The crystal was a non-merohedral twin with the two domains related by -179.7604° about the [1.00 0.00 -0.00] direct direction at a refined percentage ratio of 59.89 (19) : 40.11 (19). The resulting data was processed with the HKLF 5 program.^[22]^ The hydrogen atoms were fixed as riding models and the isotropic thermal parameters (Uiso) based on the Ueq of the parent atom. The structure was solved by direct methods using ShelXT^[18]^ and refined with ShelXL^[19]^ using a least squares method. Olex2 software was used as the solution, refinement and analysis program.^[20]^

**Crystal Data** for C_54_H_64_N_8_O_4_ (*M*= 889.13 g/mol): triclinic, space group P-1 (no. 2),
*a* = 5.46410(10) Å, *b* = 10.5953(2) Å, *c* = 20.3880(4) Å, *α* = 88.853(2)°, *β* = 83.483(2)°,
*γ* = 81.039(2)°, *V*= 1158.39(4) Å^3^, *Z* = 1, *T* = 100.00(10) K, μ(Cu Kα) = 0.647 mm^-1^,
*Dcalc* = 1.275 g/cm^3^, 8618 reflections measured (9.522° ≤ 2Θ ≤ 146.898°), 8618 unique
(R_sigma_ = 0.0303) which were used in all calculations. The final *R*_1_ was 0.0978 (I > 2σ(I)) and *wR*_2_ was 0.2343 (all data).


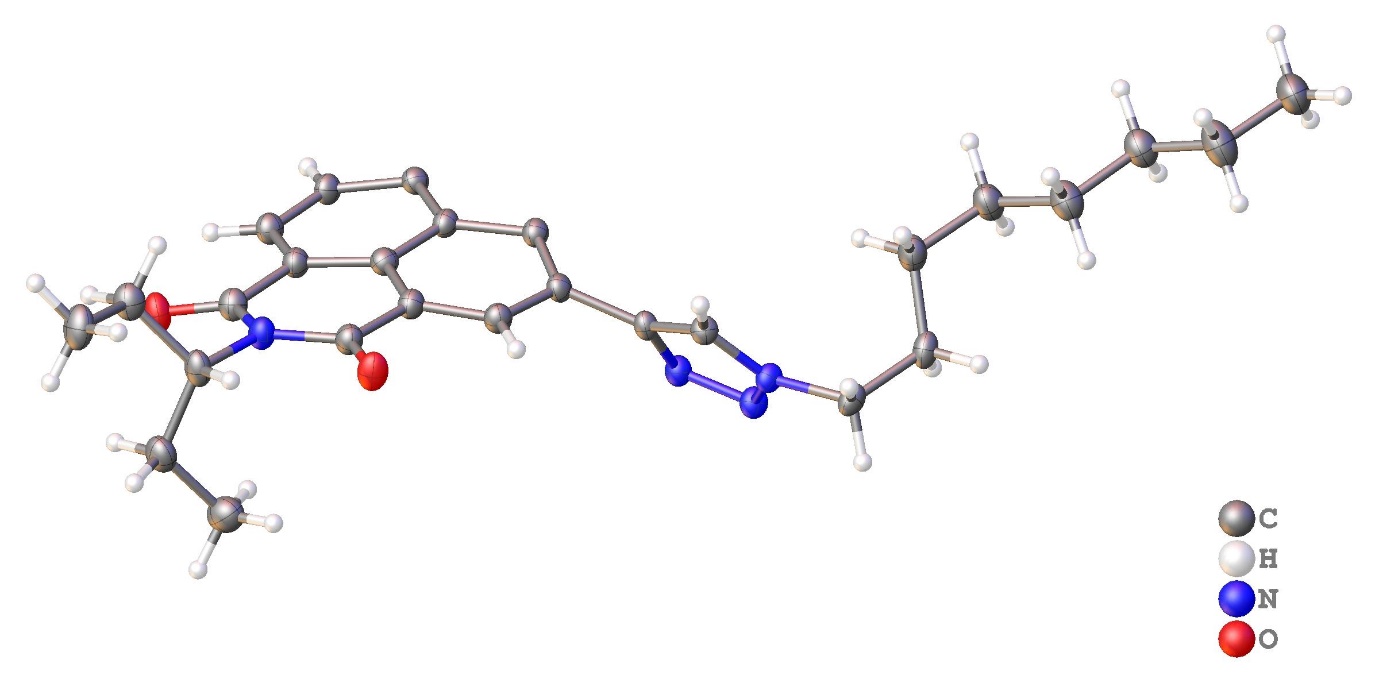


**Supplementary Figure 7‑3:** Asymmetric unit of single crystals of PDI **3a**. Thermal ellipsoids indicate 50% probability.


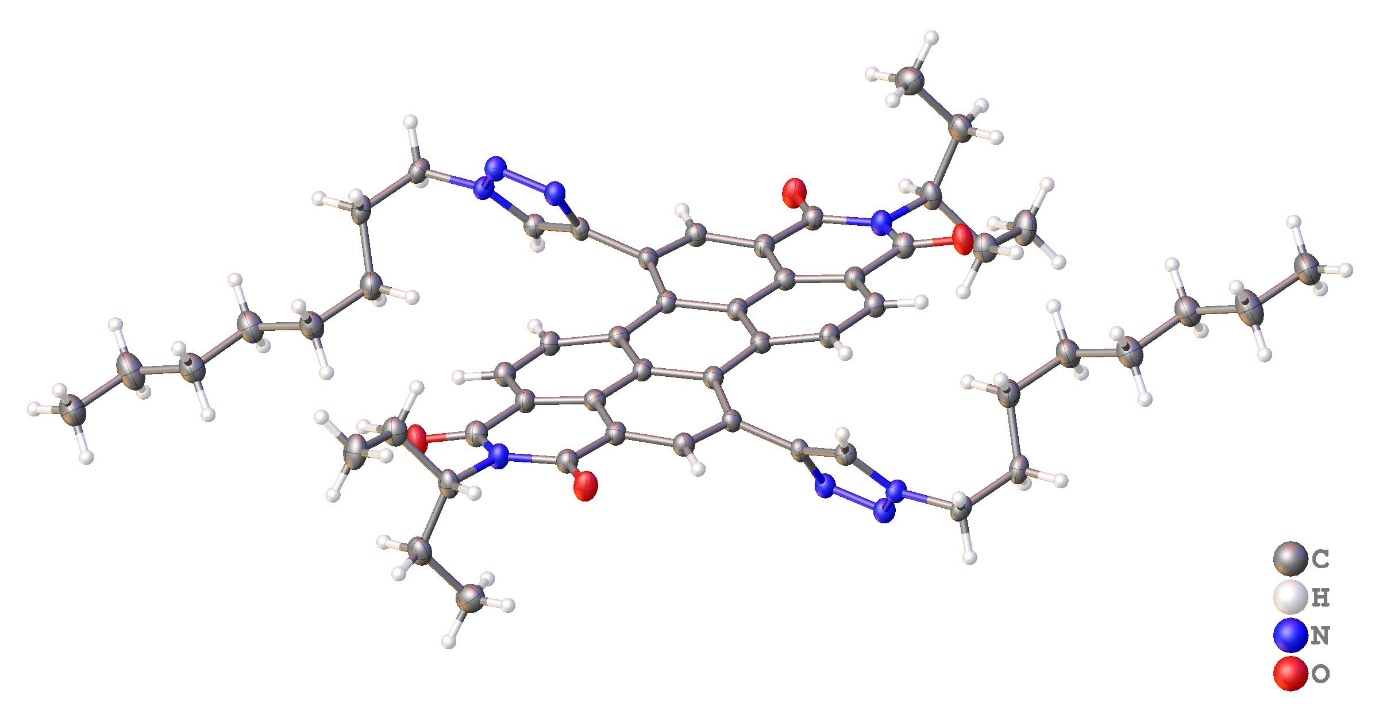


**Supplementary Figure 7‑4:** Fully grown structure of single crystals of compound **3a**, with thermal ellipsoids indicating 50% probability.


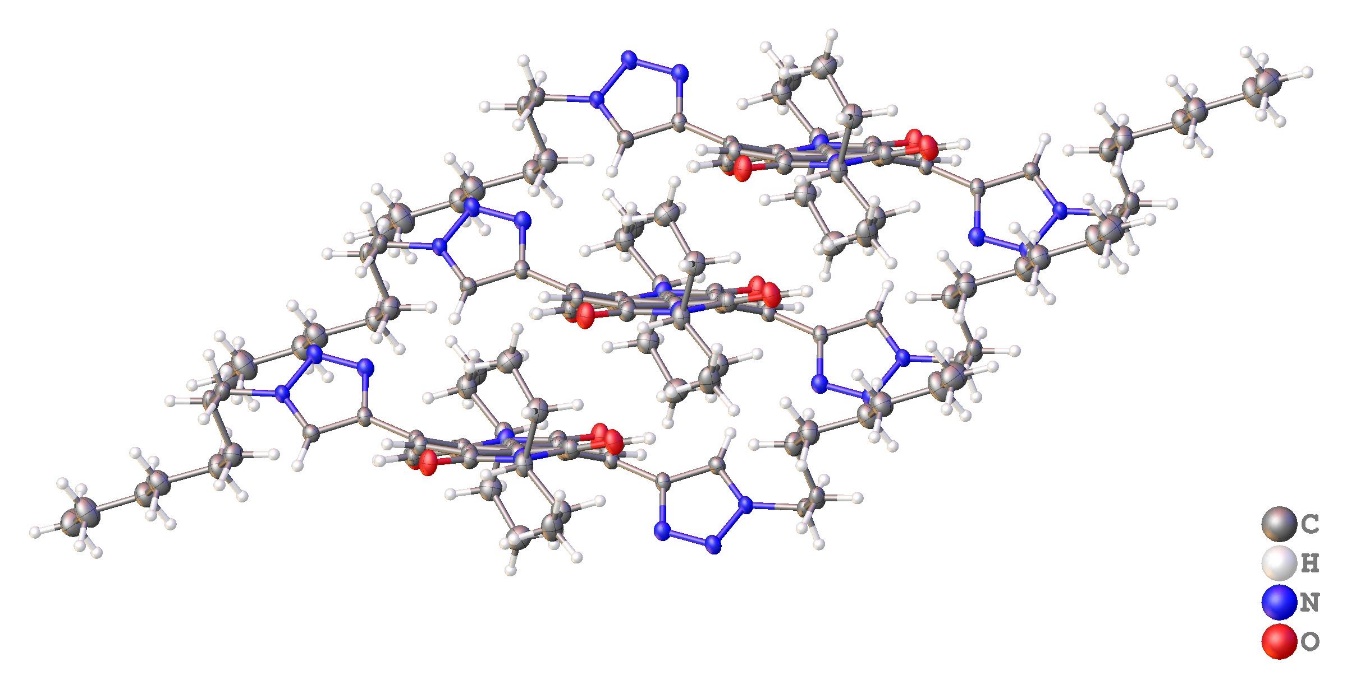


**Supplementary Figure 7‑5:** Packing of PDI units found in single crystals of **3a**.

### 1,7-di(Octyl-methyltriazolium)-C5-PDI 4a

Single crystals of 1,7-bis(octyl-methyltriazolium)-C5-PDI **4a** were prepared by vapour diffusion from a solution of **4a** in MeOH with hexane as the antisolvent. The resulting crystals were found to be thin plates, the majority of which were twinned, and the quality of the crystals could not be improved, even after multiple crystallisation attempts.

Single crystal X-ray diffraction experiments for those crystals were performed by the UK EPSRC National Crystallography Service at the University of Southampton^[21]^ on a Rigaku 007HF diffractometer with HF Varimax confocal mirrors, an UG2 goniometer and HyPix 6000HE detector at 100 K using Cu K_α_ radiation and processed using CrysAlisPro 1.171.43. The crystals were also tested at the I19 beamline at the UK Diamond Light Source, but the crystals did not survive the synchrotron radiation.

The resulting crystals showed very little diffraction at high angles, resulting from the poor crystal quality, and related to the large degree of disorder found within the structure arising from the C8-alkyl chains on the molecule. The disorder of these alkyl chains could not be accurately modelled, due to a lack of electron density. Chains were modelled along residual electron density as accurately as possible, however three chains appear truncated as a result of the lack of electron density. Constraints and restraints were used as seen fit. The residual void space in the structure is likely also occupied by solvent molecules, which also could not be modelled due to the lack of electron density. No solvent mask was applied, as that negatively affected the modelling of the alkyl chains.

The hydrogen atoms were fixed using a riding model and the isotropic thermal parameters (Uiso) based on the Ueq of the parent atom. The structure was solved by direct methods using ShelXT^[18]^ and refined with ShelXL^[19]^ using a least squares method. Olex2 software was used as the solution, refinement and analysis program.^[20]^

**Crystal Data** for C_56_H_70_F_12_N_8_O_4_P_2_ (*M*=1209.14 g/mol): triclinic, space group P-1 (no. 2),
*a* = 19.8677(6) Å, *b* = 25.9830(5) Å, *c* = 26.3984(4) Å, *α* = 105.956(2)°, *β* = 99.704(2)°,
*γ* = 106.372(2)°, *V*= 12115.7(5) Å^3^, *Z* = 8, *T* = 100.01(11) K, μ(Cu Kα) = 1.414 mm^-1^,
*Dcalc* = 1.326 g/cm^3^, 154141 reflections measured (4.81° ≤ 2Θ ≤ 117.86°), 34667 unique
(*R*_int_ = 0.1188, R_sigma_ = 0.1091) which were used in all calculations. The final *R*_1_ was 0.1438
(I > 2σ(I)) and *wR*_2_ was 0.4288 (all data).


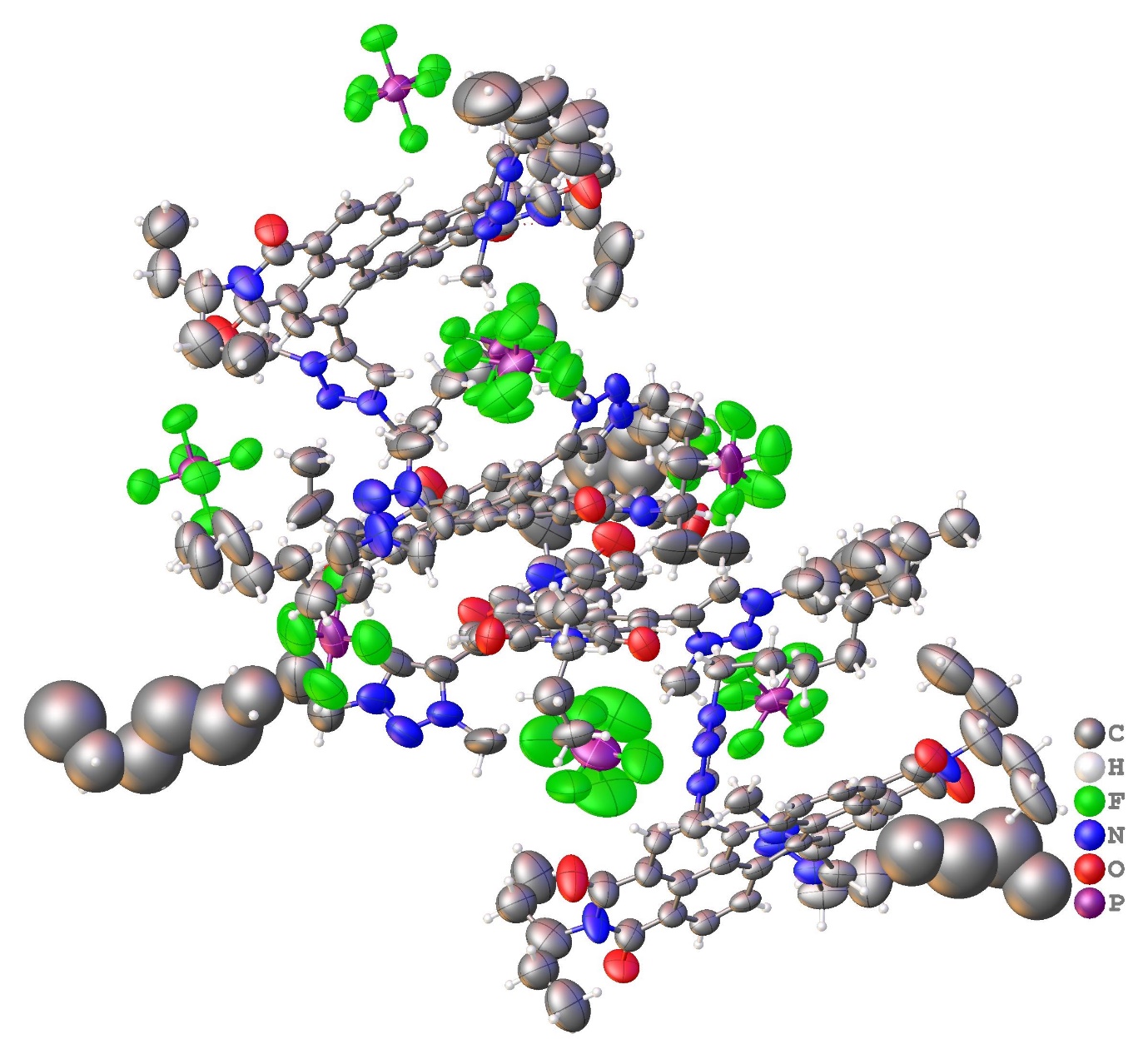


**Supplementary Figure 7‑6:** Asymmetric unit of crystals of **4a** grown from MeOH/hexane. Thermal ellipsoids show 50% probability. Large ellipsoids for alkyl chains arises from positional disorder of the C8 chains that could not be accurately modelled.


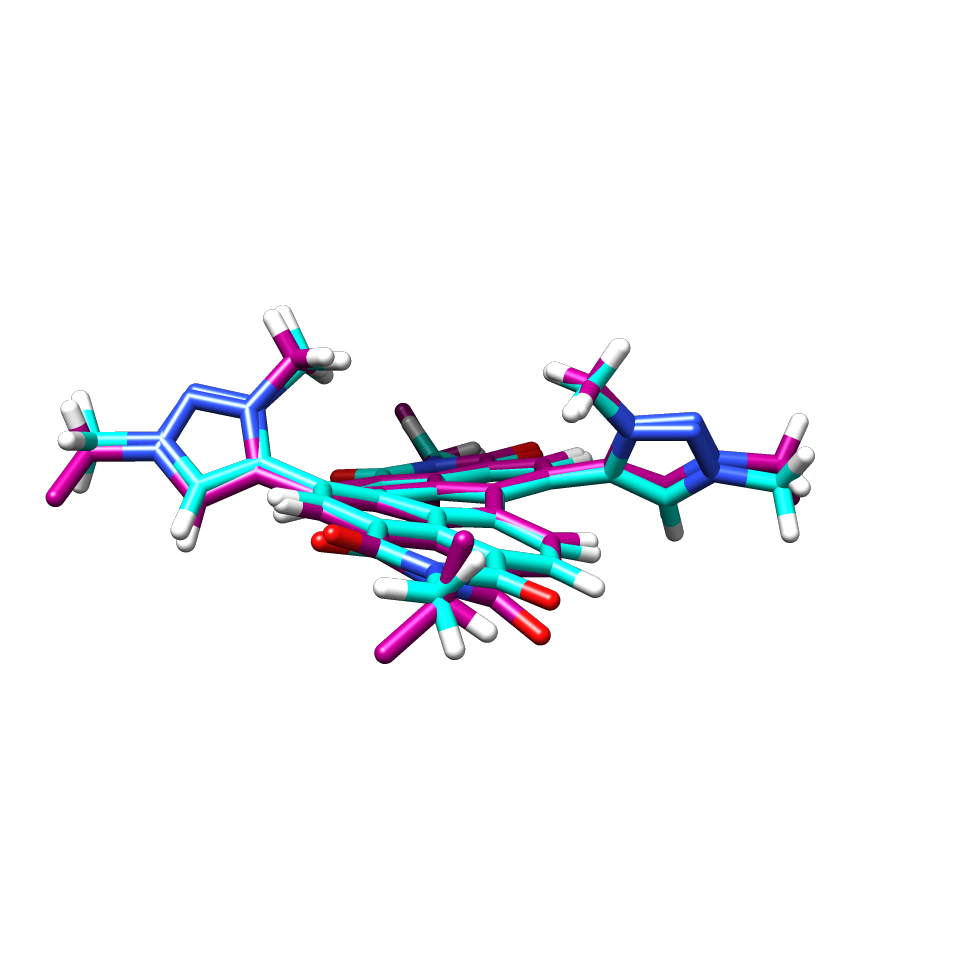


**Supplementary Figure 7‑7:** Overlay of the syn-isomer found in the crystal structure (magenta) with the DFT structure (blue). Only small differences are observed. Alkyl-chains were removed for clarity.


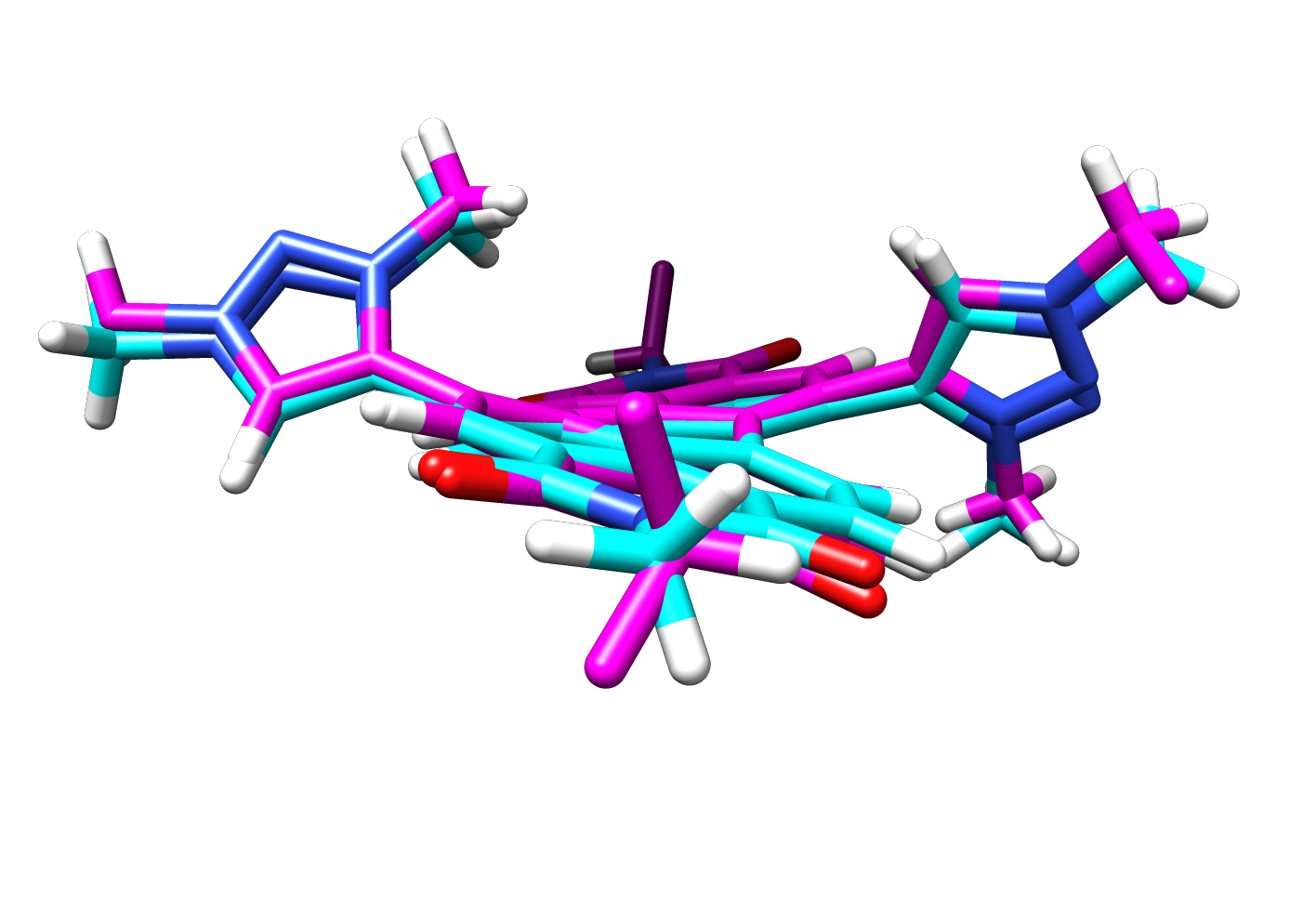


**Supplementary Figure 7‑8:** Overlay of the anti-isomer found in the crystal structure (magenta) with the DFT structure (blue). Only small differences are observed. Alkyl-chains were removed for clarity.


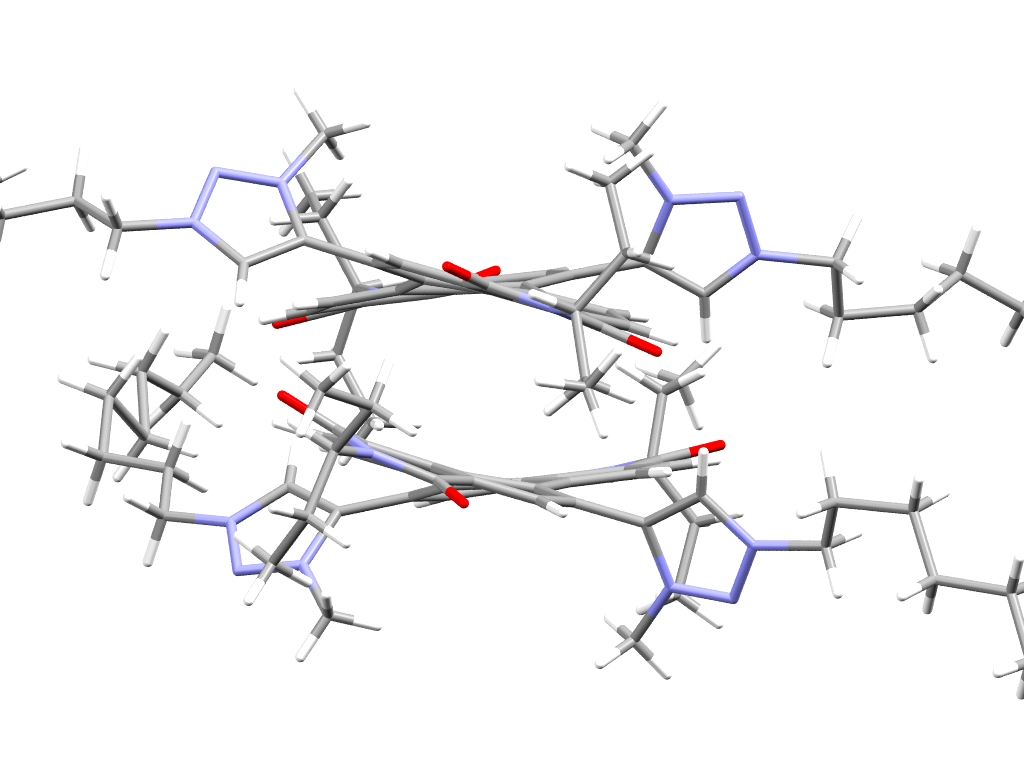


**Supplementary Figure 7‑9:** syn-syn (M-homochiral) dimer found in the single crystal structure.


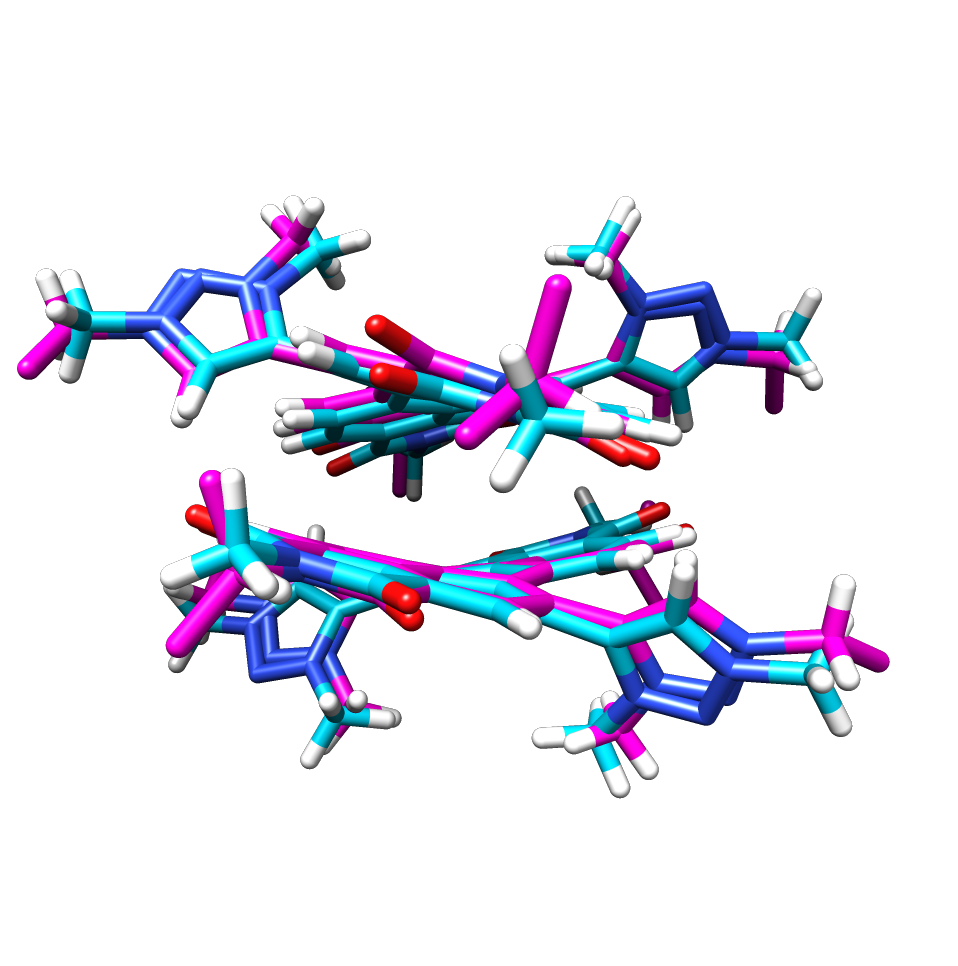


**Supplementary Figure 7‑10:** Overlay of the syn-syn dimer found in the crystal structure (magenta) with the dimer structure predicted by DFT (blue). Alkyl-chains were removed for clarity.


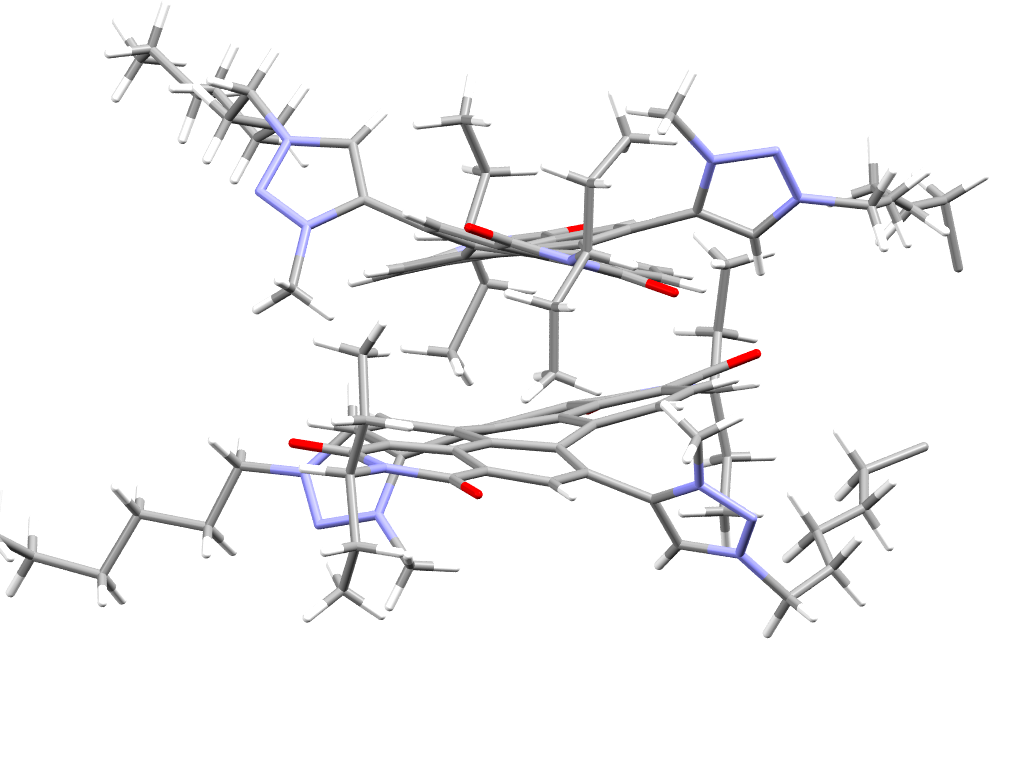


**Supplementary Figure 7‑11:** Anti-anti (M-homochiral) dimer observed in the single crystal structure.

# Mass Spectra

### 1,7- and 1,6-Br_2_-C5-PDI 1a/b


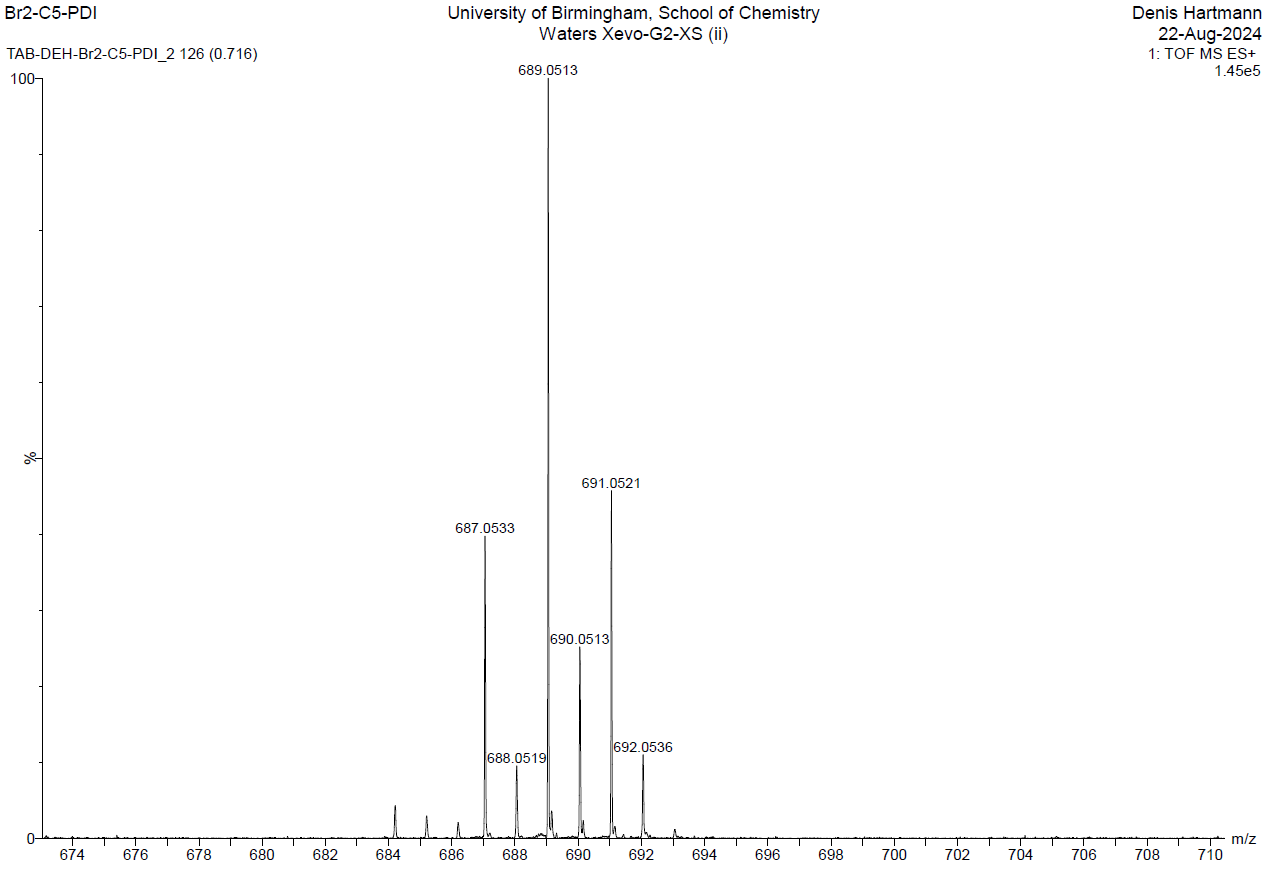


### 1,7-di(Trimethylsilylacetylene)-C5-PDI 2a


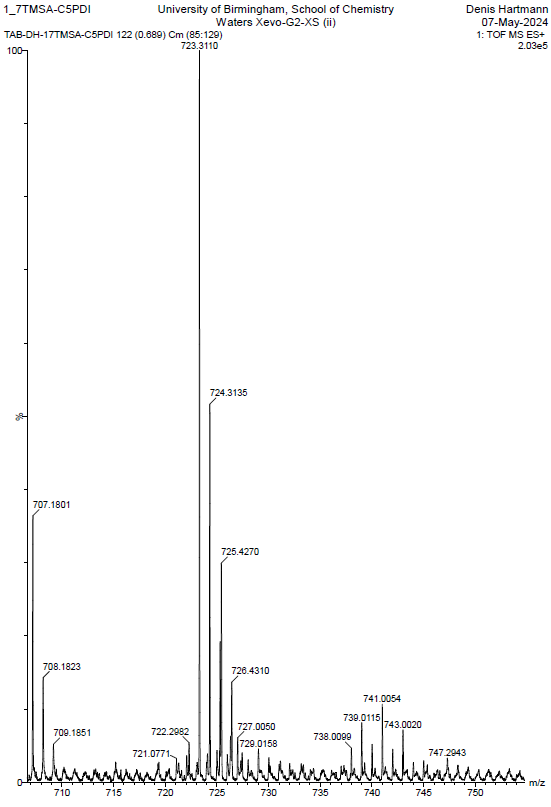


### 1,6-di(Trimethylsilylacetylene)-C5-PDI 2b


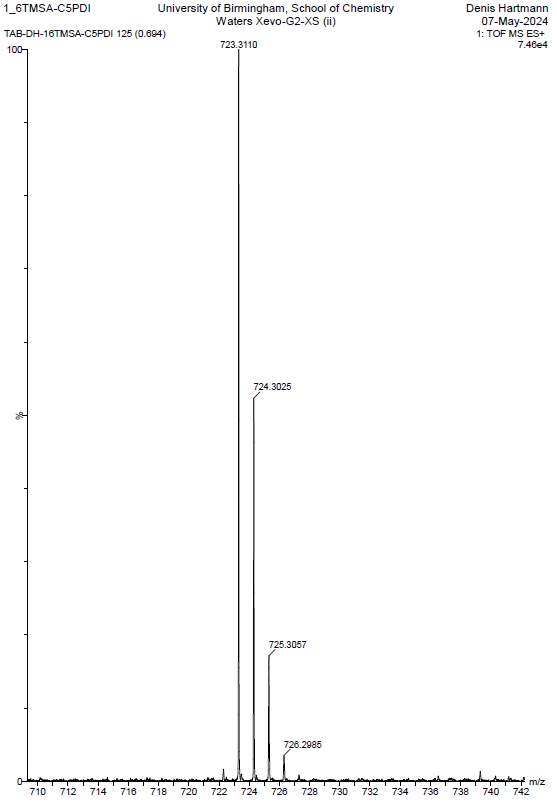


### 1,7-di(Octyltriazole)-C5-PDI 3a


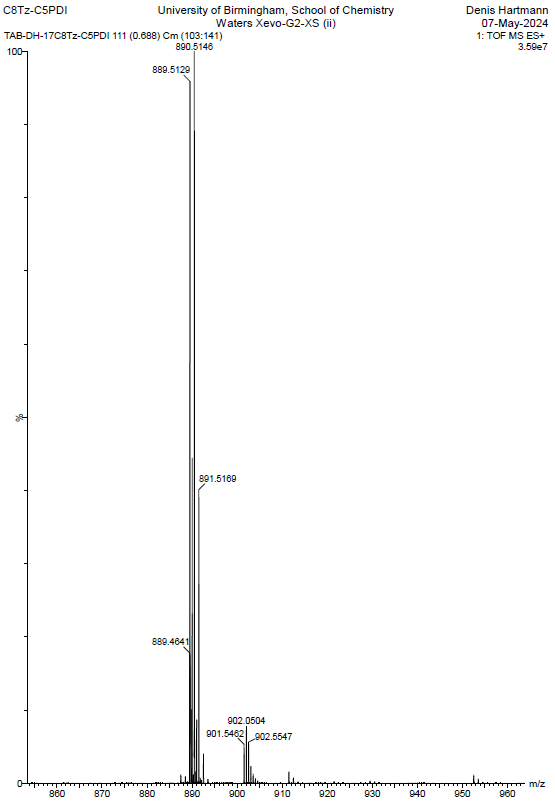


### 1,6-di(Octyltriazole)-C5-PDI 3b


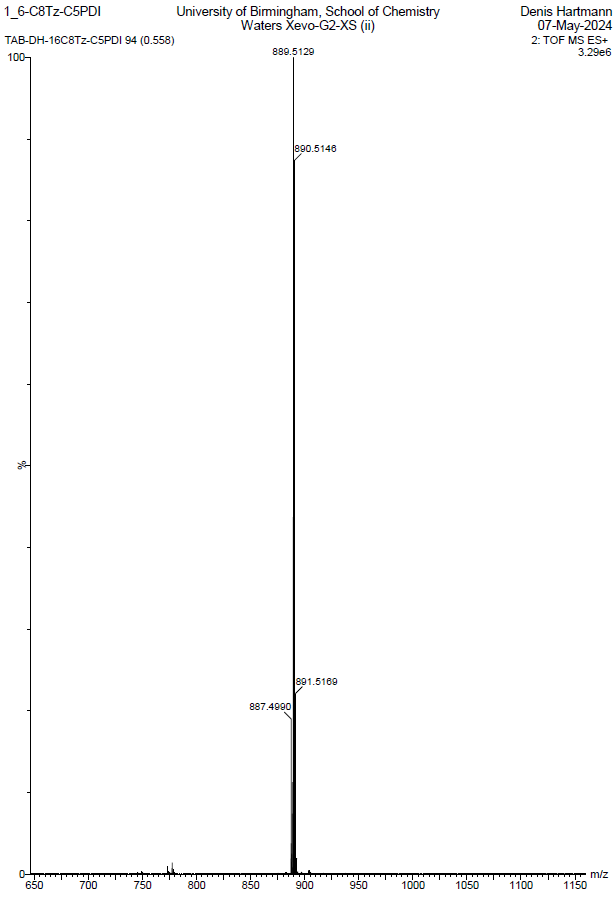


### 1,7-di(Octyl-methyltriazolium)-C5-PDI Iodide S1


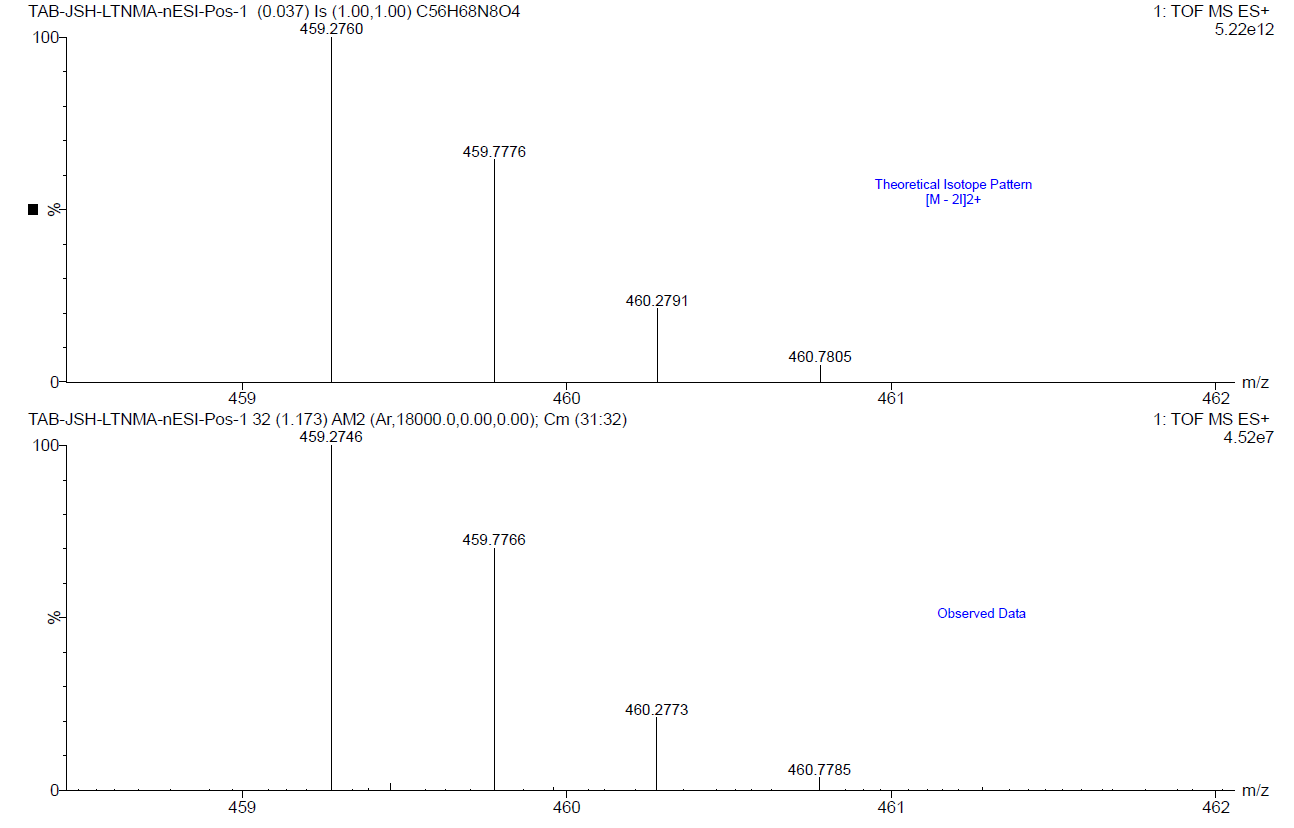


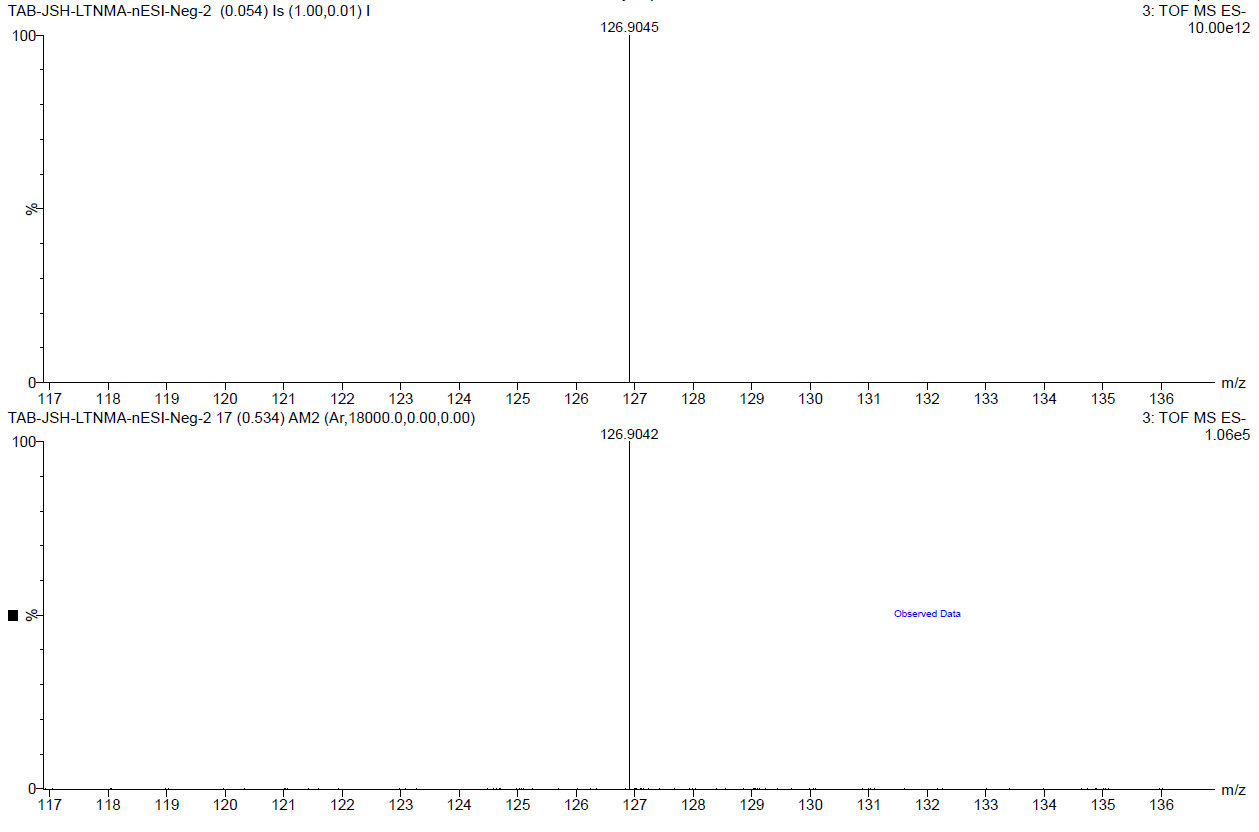


### 1,7-di(Octyl-methyltriazolium)-C5-PDI PF_6_ 4a


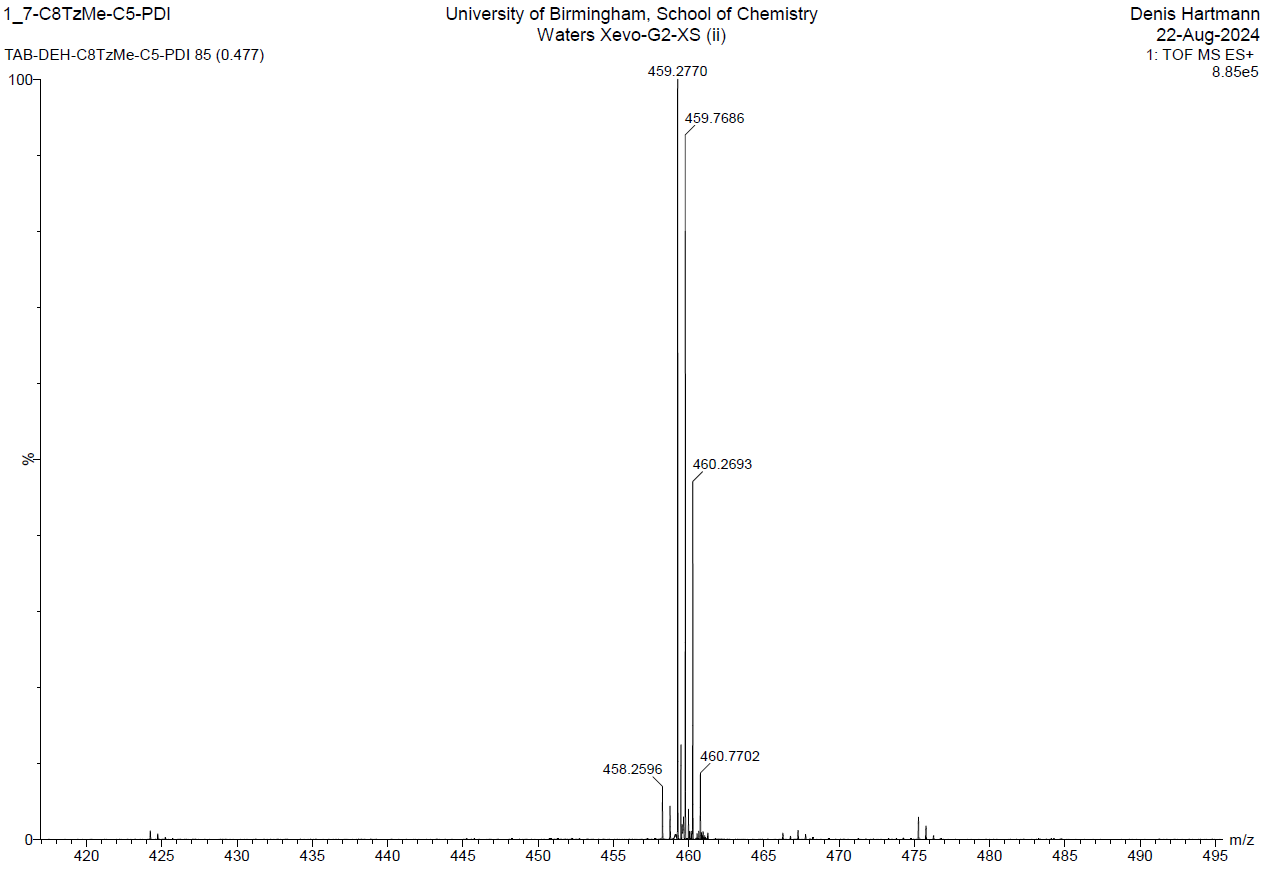


### 1,6-di(Octylmethyltriazolium)-C5-PDI PF_6_ 4b


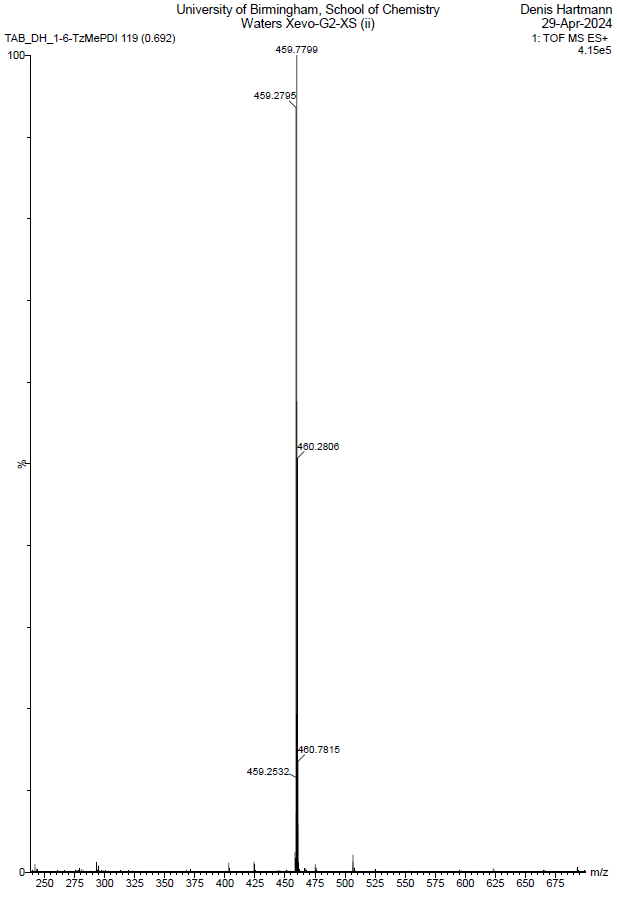


### 1,7-di(Octyltriazole)-C5-CDI S2


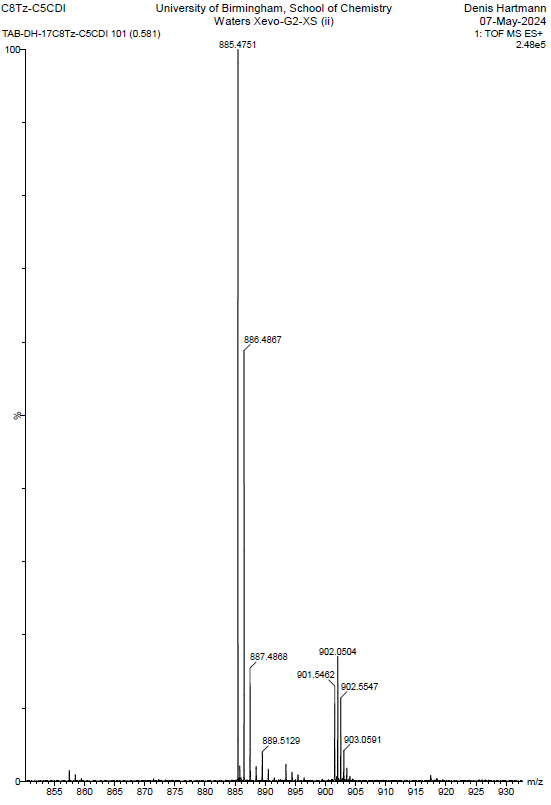


### 1,7-Br_2_ -C11-PDI S3


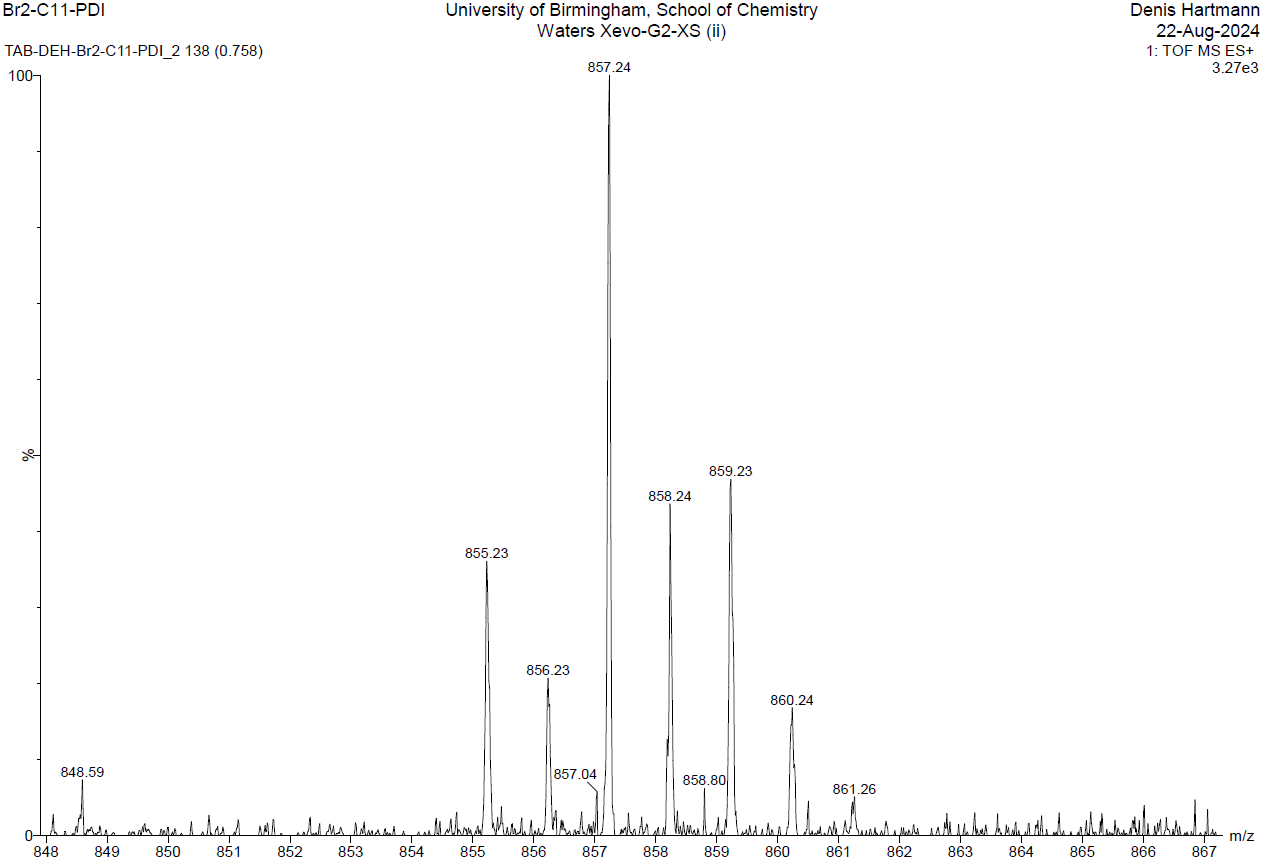


### 1,7-di(Trimethylsilylacetylene)-C11-PDI S4


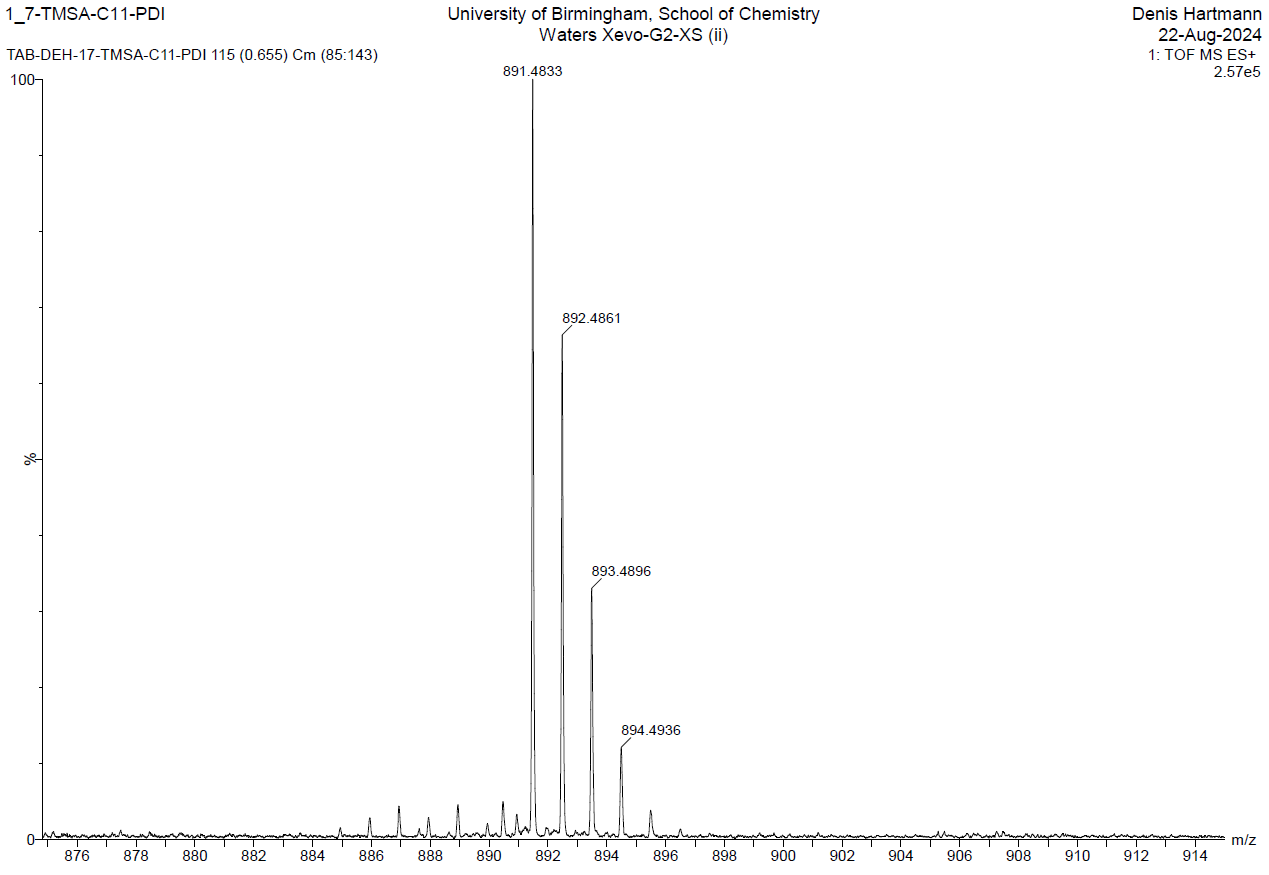


### 1,7-di(Octyltriazole)-C11-PDI 5


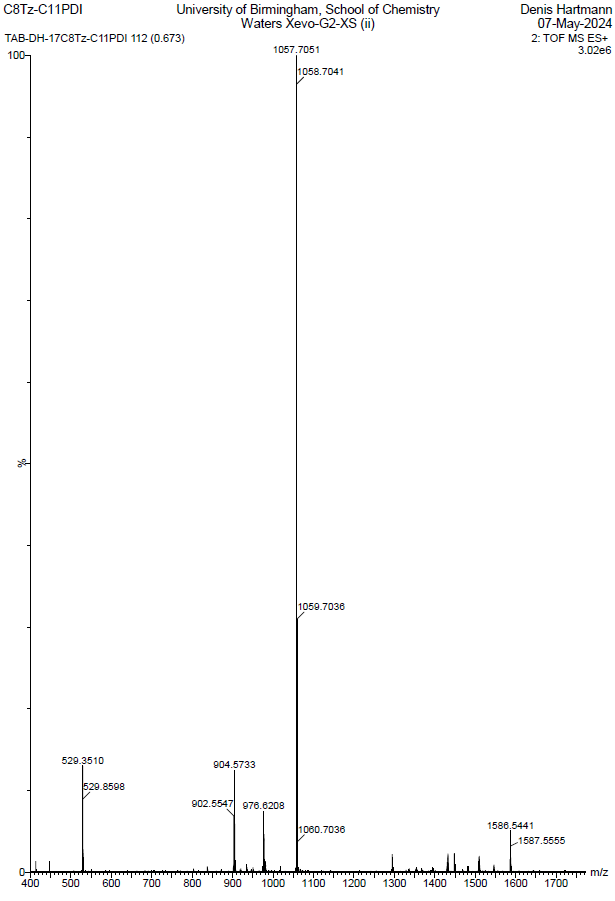


### 1,7-di(Octyltriazole)-C11-CDI 6


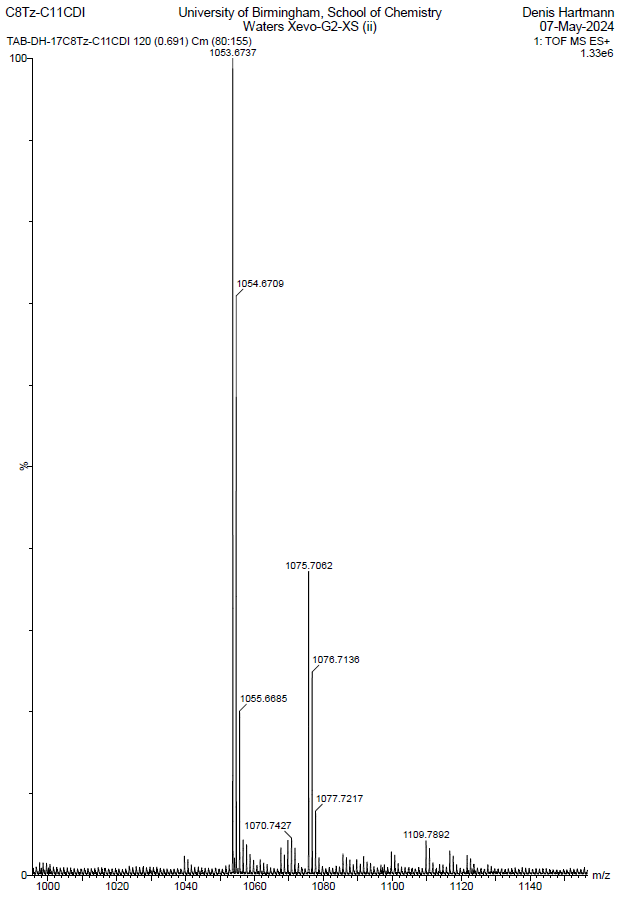


# References

[1] N. V. Handa, K. D. Mendoza, L. D. Shirtcliff, *Org. Lett.* **2011**, *13*, 4724–4727.

[2] S. E. Penty, M. A. Zwijnenburg, G. R. F. Orton, P. Stachelek, R. Pal, Y. Xie, S. L. Griffin, T. A. Barendt, *J. Am. Chem. Soc.* **2022**, *144*, 12290–12298.

[3] C. D. Schmidt, N. Lang, N. Jux, A. Hirsch, *Chemistry – A European Journal* **2011**, *17*, 5289–5299.

[4] J. Y. C. Lim, I. Marques, V. Félix, P. D. Beer, *J. Am. Chem. Soc.* **2017**, *139*, 12228–12239.

[5] M. Bursch, J.-M. Mewes, A. Hansen, S. Grimme, *Angewandte Chemie International Edition* **2022**, *61*, e202205735.

[6] J. G. Brandenburg, C. Bannwarth, A. Hansen, S. Grimme, *The Journal of Chemical Physics* **2018**, *148*, 064104.

[7] F. Weigend, R. Ahlrichs, *Phys. Chem. Chem. Phys.* **2005**, *7*, 3297–3305.

[8] P. Pracht, F. Bohle, S. Grimme, *Phys. Chem. Chem. Phys.* **2020**, *22*, 7169–7192.

[9] C. Bannwarth, S. Ehlert, S. Grimme, *J. Chem. Theory Comput.* **2019**, *15*, 1652–1671.

[10] S. Ehlert, M. Stahn, S. Spicher, S. Grimme, *J. Chem. Theory Comput.* **2021**, *17*, 4250–4261.

[11] S. Grimme, J. Antony, S. Ehrlich, H. Krieg, *The Journal of Chemical Physics* **2010**, *132*, 154104.

[12] V. Barone, M. Cossi, *J. Phys. Chem. A* **1998**, *102*, 1995–2001.

[13] S. Grimme, A. Hansen, S. Ehlert, J.-M. Mewes, *The Journal of Chemical Physics* **2021**, *154*, 064103.

[14] G. Henkelman, B. P. Uberuaga, H. Jónsson, *The Journal of Chemical Physics* **2000**, *113*, 9901–9904.

[15] G. Henkelman, H. Jónsson, *The Journal of Chemical Physics* **2000**, *113*, 9978–9985.

[16] L. Goerigk, S. Grimme, *J. Chem. Theory Comput.* **2011**, *7*, 291–309.

[17] J.-D. Chai, M. Head-Gordon, *The Journal of Chemical Physics* **2008**, *128*, 084106.

[18] G. M. Sheldrick, *Acta Cryst A* **2015**, *71*, 3–8.

[19] G. M. Sheldrick, *Acta Cryst C* **2015**, *71*, 3–8.

[20] O. V. Dolomanov, L. J. Bourhis, R. J. Gildea, J. a. K. Howard, H. Puschmann, *J Appl Cryst* **2009**, *42*, 339–341.

[21] S. J. Coles, D. R. Allan, C. M. Beavers, S. J. Teat, S. J. W. Holgate, C. A. Tovee, in *21st Century Challenges in Chemical Crystallography I: History and Technical Developments* (Eds.: D.M.P. Mingos, P.R. Raithby), Springer International Publishing, Cham, **2020**, pp. 69–140.

[22] M. Bolte, *J Appl Cryst* **2004**, *37*, 162–165.
